# The cervical transcriptome changes during the menstrual cycle but does not predict the window of implantation

**DOI:** 10.3389/frph.2023.1224919

**Published:** 2023-07-14

**Authors:** Amruta D. S. Pathare, Merli Saare, Alvin Meltsov, Ankita Lawarde, Vijayachitra Modhukur, Aive Kalinina, Aire Sekavin, Viktorija Kukushkina, Helle Karro, Andres Salumets, Maire Peters

**Affiliations:** ^1^ Department of Obstetrics and Gynecology, Institute of Clinical Medicine, University of Tartu, Tartu, Estonia; ^2^ Competence Centre on Health Technologies, Tartu, Estonia; ^3^ Department of Genetics and Cell Biology, GROW School for Oncology and Developmental Biology, Maastricht University, Maastricht, Netherlands; ^4^ Institute of Clinical Medicine, University of Tartu, Tartu, Estonia; ^5^ South Estonia Hospital, Võru, Estonia; ^6^ Women’s Clinic, Tartu University Hospital, Tartu, Estonia; ^7^ Institute of Genomics, University of Tartu, Tartu, Estonia; ^8^ Division of Obstetrics and Gynecology, Department of Clinical Science, Intervention and Technology (CLINTEC), Karolinska Institutet, and Karolinska University Hospital, Stockholm, Sweden

**Keywords:** cervix uteri, endometrium, menstrual cycle, molecular diagnostic techniques, transcriptome

## Abstract

**Introduction:**

The expression of genes in female reproductive organs is influenced by the cyclic changes in hormone levels during the menstrual cycle. While the molecular changes in the endometrium that facilitate embryo implantation have been extensively studied, there is limited knowledge about the impact of the menstrual cycle on cervical cells. Cervical cells can be easily and routinely collected using a cytobrush during gynecological examination, offering a standardized approach for diagnostic testing. In this study we investigated how the transcriptome of cervical cells changes during the menstrual cycle and assessed the utility of these cells to determine endometrial receptivity.

**Methods:**

Endocervical cells were collected with cytobrushes from 16 healthy women at different menstrual cycle phases in natural cycles and from four women undergoing hormonal replacement cycles. RNA sequencing was applied to gain insight into the transcriptome of cervical cells.

**Results:**

Transcriptome analysis identified four differentially expressed genes (DEGs) between early- and mid-secretory samples, suggesting that the transcriptome of cervical cells does not change significantly during the opening of the implantation window. The most differences appeared during the transition to the late secretory phase (2136 DEGs) before the onset of menstruation. Cervical cells collected during hormonal replacement cycles showed 1899 DEGs enriched in immune system processes.

**Conclusions:**

The results of our study suggested that cervical cells undergo moderate transcriptomic changes throughout the menstrual cycle; however, these changes do not reflect the gene expression pattern of endometrial tissue and offer little or no potential for endometrial receptivity diagnostics.

## Introduction

Our understanding of female reproductive biology has improved significantly in recent decades, and considerable efforts have been made to reveal the molecular changes that occur in the reproductive tract and endometrium throughout the menstrual cycle. Although molecular changes driving endometrial maturation have been described in many studies (1–6), little is known about whether and how the menstrual cycle affects the transcriptome of cervical cells. The first studies using the endocervical tissues collected during hysterectomy revealed significant changes between the proliferative and secretory phases samples (7, 8), suggesting that the cervical tissue transcriptome changes during the menstrual cycle. While the endocervical tissue transcriptome showed a menstrual cycle-dependent changes, it remains unclear whether a superficial layer of cytobrush-collected cervical cells shares a similar pattern. Since cytobrush collection of cervical cells is a standardized, quick, simple, well tolerated, minimally invasive, and routinely used sampling technique in daily gynaecological practice, these cells may offer diagnostic potential in various clinical settings, e.g., to identify the window of implantation (WOI).

Endometrial receptivity tests are widely used to determine the WOI in women undergoing infertility treatment. All current receptivity tests require invasive collection of endometrial biopsies. Like other invasive procedures, endometrial biopsy collection is not completely safe. Besides general discomfort during the biopsy collection, especially in nulliparous women, it can increase risk of side effects such as infections, pain, and bleeding. Thus, less invasive and non-invasive approaches to detect WOI have been eagerly sought, but with little success so far (9, 10). Currently, the most promising source for the detection of less invasive biomarkers of WOI appears to be uterine fluid (11) as it reflects the local environment of the uterine cavity, it`s collection is less traumatic, and embryos can be transferred in the same cycle. However, sampling of uterine fluid requires standardization, as the volume and concentrations of the fluid components (blood, mucus, endometrial cells, etc.) may vary depending on the fluid collection procedure (7, 8). Thus, more stable and easily accessible sources, such as brush-collected cervical cells, could offer even better diagnostic potential.

To the best of our knowledge, no studies have evaluated the suitability of brush-collected cervical cells for endometrial receptivity testing. In the current study, we used whole transcriptome RNA sequencing to reveal the gene expression patterns of cervical cells collected throughout the menstrual cycle, with a specific aim of finding potential markers for a minimally invasive endometrial receptivity test.

## Materials and methods

The study was approved by the Research Ethics Committee of the University of Tartu, Estonia (No. 302/T-4 and 330M-8) and written informed consent was obtained from all participants.

### Patient selection and sample collection

Endometrial and cervical cell samples were collected from 20 individuals, including 16 healthy women from the South Estonian Hospital (Võru, Estonia) and four women undergoing infertility treatment at the Tartu University Hospital (Tartu, Estonia).

**Figure 1:**
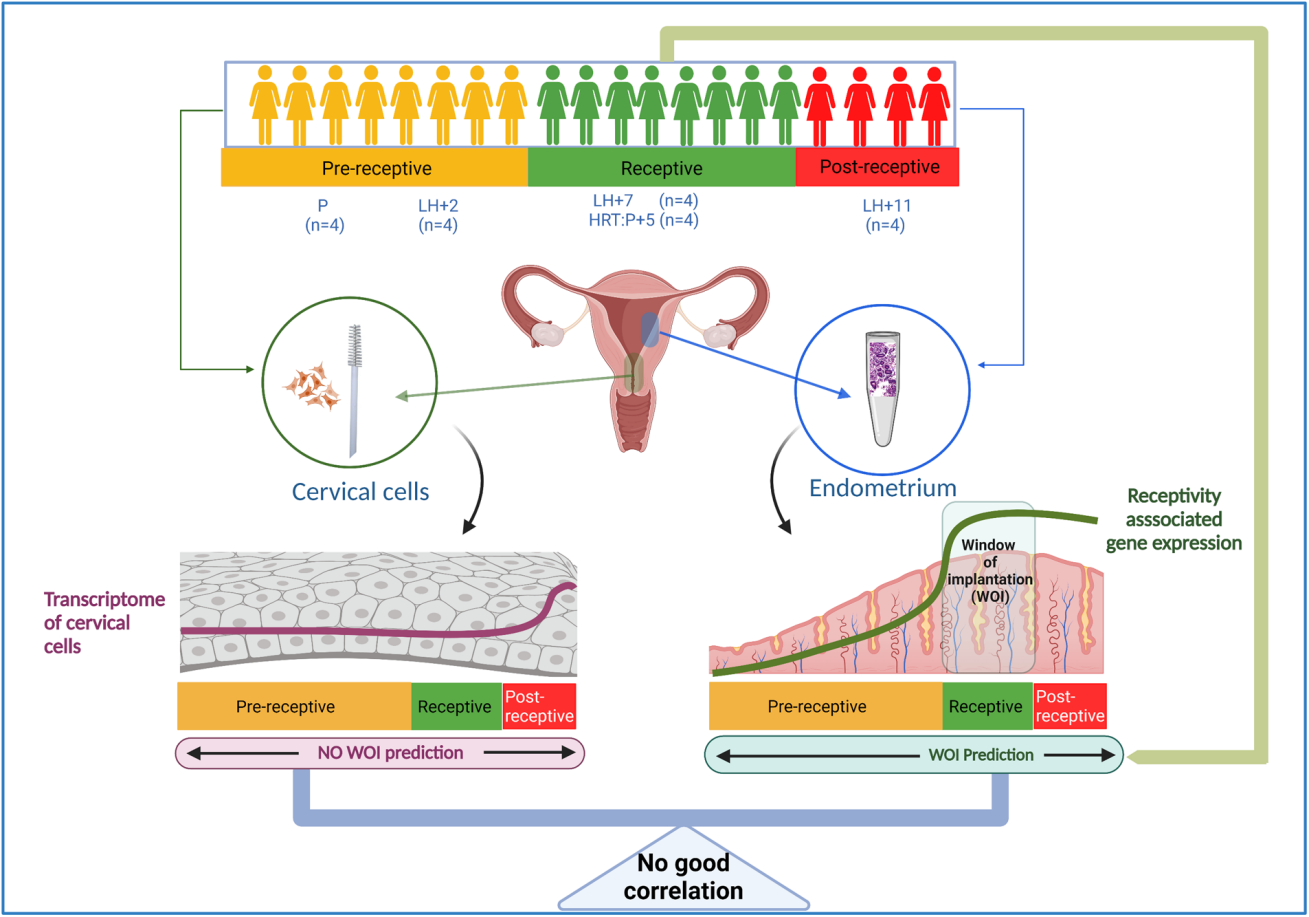
Outline of the study. Paired endometrial tissue and cervical cell samples were collected from 20 individuals; from healthy women in the proliferative phase (P, *n* = 4), at LH + 2 (*n* = 4), at LH + 7 (*n* = 4), and at LH + 11 (*n* = 4), and from four women five days after progesterone treatment in hormone replacement cycles (HRC: *P* + 5). RNA was isolated from cervical cells, and the whole transcriptome was sequenced and analyzed. The endometrial receptivity analysis was done by beREADY test (12) to confirm the receptivity status of studied endometrial samples. LH, luteinizing hormone, HRC, hormone replacement cycle.

All healthy women had negative screening results for sexually transmitted diseases, had no uterine pathologies, no signs of endometriosis or polycystic ovary syndrome, and had at least one live-born child. The menstrual cycle phase was confirmed by menstrual cycle history and luteinizing hormone (LH) peak measurement using BabyTime hLH urine cassette (Pharmanova) and histological evaluation of biopsies according to Noyes’ criteria. None of the women had used hormonal medications for at least three months. A total of 16 paired tissue and cervical cell samples were collected: four pairs from the proliferative (P) phase (cycle days 7–10), and four from each secretory phase stage: early-secretory (two days after LH peak, LH + 2), mid-secretory (seven days after LH peak, LH + 7) and late-secretory (11 days after LH peak, LH + 11) phases (Figure 1, Table 1).

**Table 1:** General characteristics of the study participants.

Study group	Age (years ± SD)	BMI kg/m^2^
Proliferative (*n* = 4)	33 ± 7.1	21.3 ± 1.3
LH + 2 (*n* = 4)	34.7 ± 3.2	27.6 ± 1.9
LH + 7 (*n* = 5)	33 ± 6.1	24.5 ± 3.2
LH + 11 (*n* = 3)	33.6 ± 1.2	26.1 ± 3.7
HRC: *P* + 5 (*n* = 4)	33.5 ± 5.9	25.0 ± 5.6

BMI, body mass index; LH, Luteinizing hormone; HRC, hormone replacement cycle.

In addition, four women undergoing infertility treatment at hormone replacement cycle (HRC): *P* + 5 [5 days after progesterone (P4) administration] and having a history of more than three unsuccessful IVF cycles with good-quality embryos were recruited into the study. All these women underwent endometrial receptivity testing with the beREADY test (12), which confirmed receptivity status.

In all study participants, cervical cell samples were collected by Kito-brushes (Kaltek S.R.L) before endometrial biopsy, and endometrial tissue biopsies were collected using Pipelle® flexible suction catheter. Both samples were placed immediately into RNAlater (Thermo Fisher Scientific, USA), and after 24-hour incubation at 4°C, were stored at −80°C until use.

### RNA extraction from endometrial tissue and cervical cells

Endometrial tissue total RNA was extracted using RNeasy Mini kit (Qiagen) according to the manufacturer’s protocol. Purified RNA quality (RIN) was assessed with Qubit RNA IQ Assay (Thermo Fisher Scientific). Samples with an RNA integrity number (RIN) ≥ 7 were eligible for further analysis.

Cervical cells’ RNA was isolated by RNeasy Micro kit (Qiagen) according to the manufacturer’s protocol. Samples with RIN ≥ 6 were considered eligible for further analysis.

### Endometrial receptivity genes expression profiling

Gene expression profiling of 67 endometrial receptivity-associated genes was done by commercial beREADY test (www.beready.ee, Competence Centre on Health Technologies, Tartu, Estonia) (12) to confirm the menstrual cycle date of the endometrial samples.

### RNA library preparation and sequencing

Cervical cell RNA libraries were prepared with the TruSeq Stranded mRNA Library Prep kit (Illumina) using 250–500 ng of RNA as input material. Samples were paired-end sequenced with a read length of 2 × 75 bp on a NextSeq 500 (Illumina) instrument according to the manufacturer’s instructions at the Core Facility of Genomics, University of Tartu (Estonia).

The raw sequencing data were analysed with the nf-core pipeline version 3.5 (13). Reads were aligned to the GRCh37 human genome with the STAR aligner (v2.7.10a) (14), and quantification was performed using RSEM (v1.3.3). The overall sequencing and alignment passed the quality checks (FastQC and MultiQC tools). The total number of sequenced reads ranged from 26 to 70 million (M). The alignment score was high for most of the samples. On average 22 M sequences were aligned to the genome, with an average of 92% of the reads being uniquely aligned; 83.6% of the uniquely aligned reads were aligned to exons and 10% of reads aligned to introns. In the next step, low-expressed genes were filtered out in two steps. In the first step, genes with a raw read count of zero were removed, and in the second step, the mean Transcript-per-Million (mean-TPM) count per group for each gene was calculated. Genes with mean-TPM > 1 were retained and raw counts of these genes were used for further downstream analyses. Differentially expressed genes (DEGs) were identified using the Bioconductor package DESeq2 (v.1.36.0) (15) with default parameters, and DEGs were defined as follows: (a) with Benjamini-Hochberg adjusted *P* value ≤0.01 (b) with at least 2-fold difference between compared groups. For visualisation and dimensionality reduction, the VST function from DESeq2 (v.1.36.0) was applied on the raw count matrix of highly expressed genes after the gene filtering steps. Then the matrix of transformed and normalized count was used for dimension reduction analysis with the UMAP algorithm using the umap (v.0.2.9.0) R (v.4.2.1) package and visualized using ggplot2 (v.3.4.0) in R package (https://ggplot2.tidyverse.org). Biological mechanisms underlying DEGs were investigated using g:Profiler (16).

### Cell-type enrichment analysis

To computationally disentangle bulk transcriptomic data into individual cell types, the cervical cell samples enrichment analysis was performed using the xCell tool (17). The datasets from the GSE119209, GSE86491 (a total of 12 endometrial tissues), and data from 79 randomly selected tissues (2–3 samples per tissue) from the Genotype-Tissue Expression (GTEx) project were used to create a heterogenous dataset for cell-type enrichment analysis. Enrichment scores were calculated using the xCell R package, version 1.1.0. The calculated scores were visualized using the pheatmap R package, version 1.10.12.

### Gene expression validation by quantitative real-time PCR (qRT-PCR)

The expression levels of *TK1*, *PBK*, *TROAP,* and *SFRP4* genes, and two housekeeping genes (*ACTB* and *TBP*) were determined by qRT-PCR. The list of primers is available upon request. DNase treated (TURBO DNA-free™ kit, Ambion Inc.) RNA (up to 1 µg) was converted into cDNA using RevertAid First Strand cDNA Synthesis Kit (Thermo-Fisher Scientific Inc. MA, USA). qRT-PCR was performed using 1 × HOT FIREPol EvaGreen qPCR Mix Plus (Solis BioDyne, Estonia) according to the conditions specified by the manufacturer. qRT-PCR reactions were done using 7,500 Fast Real-Time PCR System (Applied Biosystems, Foster City, USA). Normalized gene expression level (*Δ*CT) differences between the cells from different menstrual cycle phases were evaluated by Student’s two-sided t-test, and *P* < 0.05 was considered a statistically significant difference.

## Results

### Endometrial receptivity and menstrual cycle phase determination

Endometrial receptivity tests can be used to specify the precise menstrual cycle phase (18), and therefore, we used the beREADY test to determine whether endometrial samples of healthy women corresponded to the expected molecular menstrual cycle phase. The results showed that all but one of the 16 endometrial samples grouped correctly. One sample from the LH + 11 (post-receptive) group showed receptive test results. Further examination of the patient’s medical records and histological evaluation of the endometrial biopsy revealed that the sample was collected on day 22 of the menstrual cycle and histological biopsy assessment confirmed the mid-secretory phase, indicating an error in the LH peak reading. Cervical cells were grouped based on the endometrial beREADY test results, and the sample with incorrect cycle phase was re-grouped into the receptive group of LH + 7 samples in further analysis. The samples of healthy women were then classified as: *P* (*n* = 4), LH + 2 (*n* = 4), LH + 7 (*n* = 5), and LH + 11 (*n* = 3). All HRC: *P* + 5 (*n* = 4) biopsies collected from infertile women showed a receptive profile with the beREADY test.

### Transcriptomes of cervical cells collected throughout the menstrual cycle

Cervical cells were analyzed from 20 individuals using RNA-seq. One *P*-phase sample was excluded as an outlier. A total of 16,176 genes were detected after filtering. The most highly expressed gene in cervical cells was *SLPI* (Secretory leukocyte protease inhibitor), previously described as the most abundant protein in cervical mucus (19). Other highly expressed top-list genes were *B2M*, *FTH1*, *HBB*, *EEF1A1*, *S100A9*, *CXCL8*, *WFDC2*, *TPT1* and *LCN2* (the list of highly expressed genes is presented in Supplementary Table S1).

**Figure 2:**
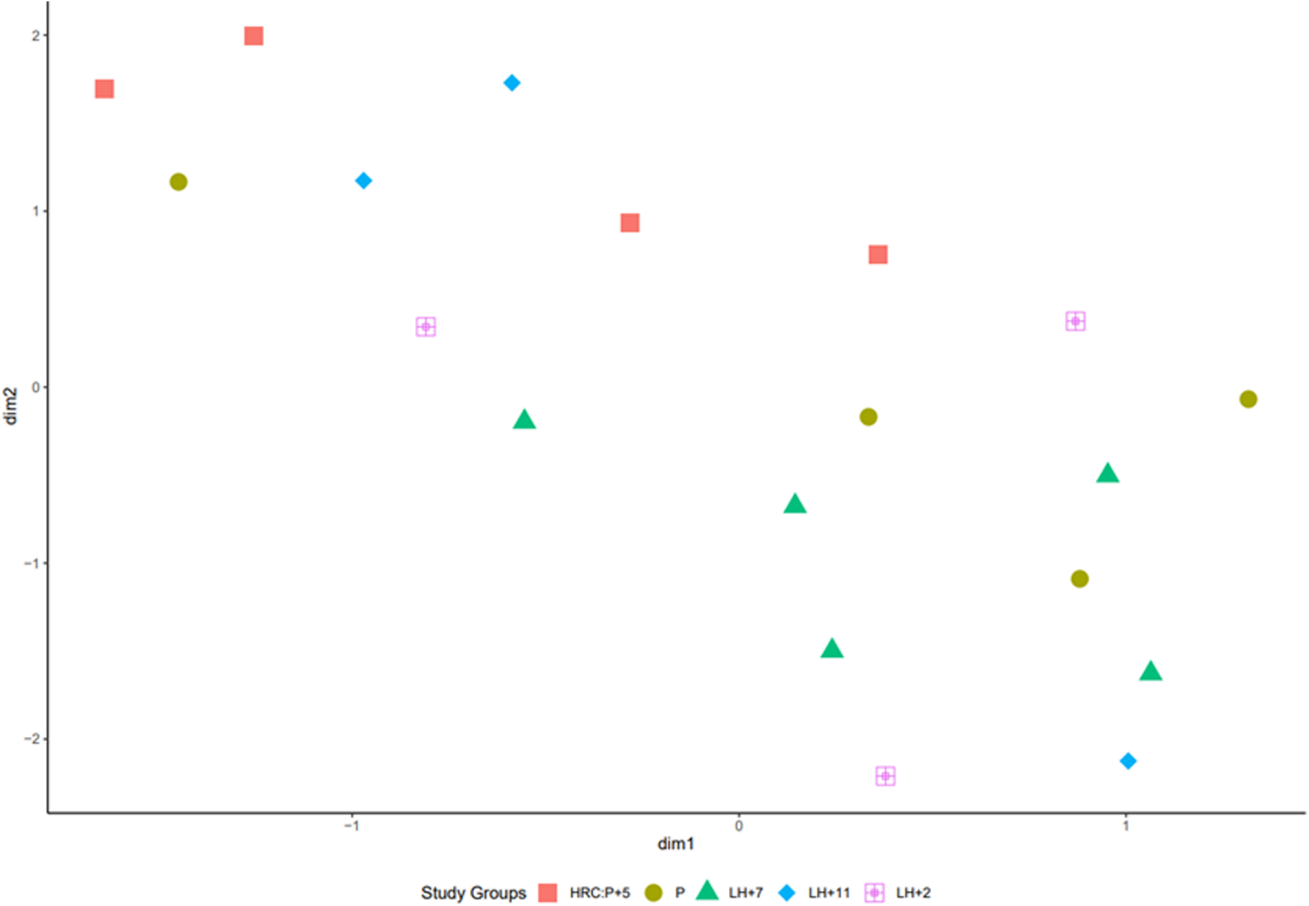
Uniform manifold approximation and projection (UMAP) of transcriptome data of 19 cervical cell samples collected at the different menstrual cycle time points. VST transformed count matrix was used to plot the UMAP projections.

UMAP was utilised to cluster the samples according to the menstrual cycle phases. Samples with similar trends in their gene expression profiles should cluster together in the plot. However, according to UMAP clustering data, the overall gene expression profiles of cervical cells showed no apparent clustering according to their collection time (Figure 2). Gene expression data from specific menstrual cycle time points were compared to assess whether there are still some differences between cervical cells in distinct phases of menstrual cycle (Table 2). When *P* samples were compared to LH + 2 samples, 26 DEGs were found, from which 11 had low expression levels (Supplementary Table S2). The comparison of LH + 2 and LH + 7 data showed only four DEGs (*KIF2C*, *CENPF*, *HLA-DRB5,* and *CUTALP*), suggesting that the transcriptome of cervical cells, unlike that of the endometrium, does not exhibit significant differences during the WOI opening. Further, we combined the *P* and LH + 2 samples into one group as they both had pre-receptive profiles in endometrial studies and compared this group with the LH + 7 group. Data analysis revealed 37 DEGs, including two genes (*CENPF* and *KIF2C)* that also emerged in the LH + 2 vs. LH + 7 comparison. Most of these genes had relatively low expression values, and 35 out of 37 were downregulated in LH + 7 samples (Supplementary Table S3). However, when we compared LH + 11 and LH + 7 samples (Supplementary Figure S1), we found 2,136 significant DEGs (Supplementary Table S4). This pattern indicates substantial transcriptional changes in cervical cells before menstruation.

**Table 2:** The number of differentially expressed genes in cervical cells throughout the menstrual cycle. Comparison between cervical cells collected via brush in different menstrual cycle phase samples.

Menstrual cycle phase group comparison	Number of DEGs FDR < 0.01	Number of DEG with TPM > 1^a^
LH + 2 vs. LH + 7	4	2
LH + 7 vs. LH + 11	2,136	1,650
*P* vs. LH + 2	26	15
*P* vs. LH + 2/LH + 7/LH + 11	15	8
P/LH + 2 vs. LH + 7	37	11
LH + 11 vs. P/LH + 2/LH + 7	1,238	914
HRC: *P* + 5 vs. LH + 7	1,899	1,623
HRC: *P* + 5 vs. LH + 2	324	241
HRC: *P* + 5 vs. LH + 11	676	615

*P*, proliferative phase, LH, luteinizing hormone, HRC, Hormone replacement cycle, DEG, differentially expressed genes, FDR, false discovery rate, TPM, transcript per million.

^a^ TPM > 1 average value within one group.

### Transcriptomes of cervical cells collected in hormonal replacement cycles

Comparison of cervical cells collected at HRC:*P* + 5 and natural cycle LH + 7 samples showed 1,899 DEGs (Supplementary Table S5). Functional annotation analysis of these DEGs revealed “immune system process” (*P* value 6.03E^−78^), “immune response” (*P* value 1.06E^−65^), “regulation of immune system process” (*P* value 1.07E^−55^) as the most relevant GO terms and “Chemokine signalling pathway” (60 genes, *P* value 6.1E^−15^), “Osteoclast differentiation” (43 genes, *P* value 5,03E^−11^) and “B cell receptor signalling” (28 genes, *P* value 1,36E^−7^) pathways as the most enriched KEGG pathways (Supplementary Table S6).

### Endometrial receptivity markers in cervical cell samples

The expression of 67 common receptivity markers (12) was analysed *in silico* from cervical cell sequencing data to see whether the receptivity genes exhibit cycle-phase specific expression pattern in cervical cells as seen in endometrial tissue. Data analysis revealed that 64 out of 67 genes were expressed in cervical cells, but none of them was statistically differentially expressed between LH + 2 and LH + 7 samples.

### Validation of sequencing data by qRT-PCR

To confirm the sequencing results and to understand how the same genes are expressed in endometrium and cervical cells, four random genes (*PBK*, *TK1*, *SFRP4,* and *TROAP*) showing differential expression between the pooled group of pre-receptive samples and LH + 7 were analyzed by qRT-PCR. Data analysis confirmed significantly different expression levels of *PBK*, *TK1*, and *SFRP4* genes in both endometrial and cervical cell samples (Supplementary Figure S2). The *TROAP* showed no significant differential expression between the cervical samples but was highly significant between endometrial samples.

### Cellular heterogeneity of the cervical cell samples

Since numerous cell types within samples can influence gene expression signatures, we applied the xCell package to determine the cellular heterogeneity of brush-collected cervical samples. The most abundant cells were epithelial and smooth muscle cells, followed by monocytes, iDCs (interstitial dendritic cells), basophils, and neutrophils. The finding that smooth muscle cells were one of the most abundant cell types in cervical cells was surprising, but according to the literature, the cervix exhibits a large proportion of functionally active smooth muscle cells, and there is a gradient in the internal and external part of the cervix (20). The complete list of scores for all specific cell types is presented in Supplementary Table S7. When the cellular distribution of samples obtained from the different menstrual cycle phases was evaluated, no clustering based on the menstrual cycle phases was seen (Supplementary Figure S3), indicating the uniform composition of cervical biopsies.

## Discussion

To the best of our knowledge, this is the first study to examine the transcriptomes of brush-collected cervical cells throughout the normal menstrual cycle and to assess the potential of cervical cells in endometrial receptivity diagnostics.

The need for less invasive molecular markers for endometrial receptivity testing has triggered researchers to search for alternative tissues that reflect menstrual cycle-related hormonal changes in endometrium and predict the onset of WOI. The cervical cells meet the requirements of a minimally invasive approach, although the cervix is distinct from the uterus and is considered a separate anatomical structure. Collection of cervical epithelial cells from the lower part of the cervical canal with a cervical sampling brush has been widely used in the molecular diagnosis of cervical cancer and detection of HPV viruses (21). Given the simple collection technique that can be easily performed during a routine outpatient visit, the cervical cells may also offer excellent diagnostic potential for receptivity evaluation.

In this pilot study, we evaluated the suitability of cervical cells for endometrial receptivity testing and found only four DEGs between the cervical cells collected before WOI opening (LH + 2) and during WOI (LH + 7). Two of these genes (*KIF2C* and *CUTALP*) had low expression levels, and *CENPF* is a pseudogene. *HLA-DRB5* expression may reflect the presence and infiltration of blood-derived immune cells in cervical cells biopsies, thus, this marker’s diagnostic value is currently unclear. Since the expression levels of the most common endometrial receptivity genes also did not differ between the LH + 2 and LH + 7 samples, cervical cells’ transcriptomics is probably unsuitable for receptivity testing. The lack of transcriptomic differences between the cervical cells collected from the follicular and luteal phase was also reported in a previous microarray study (22), which coincides our results. Our results demonstrated that the transcriptome of cervical cells is relatively uniform throughout the menstrual cycle; however, more changes occur in the late-secretory phase, when the best time for embryo implantation is already passed. We hypothesise that the extensive rearrangements in gene expression are related to the preparation of cervical cells for the physiological changes in the onset of menstruation rather than related to the WOI.

Our results showed that cervical cells collected with a cervical brush exhibit a different molecular composition compared to endocervical tissue biopsies (7, 8). During the sample collection with a cervical brush, mainly superficial cells are obtained, and the most hormone sensitive glandular cells in cervical crypts are not collected, which likely explains the differences between our and previous results. Endocervical tissue (7, 8) studies have reported significant gene-expression changes between proliferative and secretory phase samples, but no similar menstrual cycle-related changes were seen in brush-derived cervical cells (22). Like endometrial tissue, endocervical tissue contains estrogen and progesterone receptors that make it susceptible to progesterone action during the menstrual cycle (23). We noticed estrogen and progesterone receptor expression in all brush-collected samples, which is in concordance with endocervical gene expression studies (7, 8). However, there were no significant differences in estrogen and progesterone receptor expression between the proliferative and secretory phase samples. A study by Mukhopadhyay et al., found more than 200 DEGs between the proliferative and secretory phases endocervical samples (8), with moderate FDR values for all genes. Our study also noticed the differences between the proliferative and secretory samples, but the expression changes were less pronounced.

Studies have shown that in hormonally stimulated cycles, the endometrium does not reach the receptivity in the same manner, or at least at the same time, as in natural cycles (24–27), and in HRC endometrial biopsies, the acquisition of the receptivity phenotype is slower than in natural cycles. During the HRC, women receive exogenous estrogen and progesterone to mature the endometrium for embryo implantation and this may also affect the gene expression signature of cervical cells. We observed notable differences between the transcriptomes of cervical cells from healthy women and women with RIF diagnosis, with significant enrichment in genes and pathways related to the immune system. The immune system involvement and alterations in immune system-related genes/pathways in endometrial tissue of RIF patients have been reported previously (24–26). However, it is important to consider that the large number of differentially expressed genes could also be attributed to the administration of exogenous hormones during hormone replacement therapy cycles. There is evidence suggesting that HRC significantly affects the overall gene expression profile also in non-RIF patient’s endometrium (24, 27), therefore, it is very likely that hormonal stimulation can also alter the expression of cervical cells. The significant impact of HRC protocols rather than RIF/healthy status of the patients to endometrial transcriptome was also reported by Haouzi et al. (28). Hence, in order to ascertain whether the significant number of differentially expressed genes in the HRC group is a result of the RIF status or the hormonal stimulation, further investigations are required.

We acknowledge some limitations of this study. Our study group was relatively small, but we included samples of healthy women spanning the entire menstrual cycle. Also, although the biopsy collection procedure is not fully standardizable and RNA isolation from the brush-collected cervical cells samples is technically challenging (29), our analysis showed that the cellular composition of the studied samples was uniform and it is possible to obtain high-quality transcriptome data from such samples. Although transcriptional data from cervical cells are not useful for detecting WOI, they can be used in various clinical settings to answer other clinically relevant questions and to establish diagnostic tests for pathological conditions such as cervical cancer.

In conclusion, the results of our study suggested that although the transcriptome of brush-collected cervical cells exhibits changes during the menstrual cycle, it does not reflect the gene expression pattern of endometrial cells during the menstrual cycle. Therefore, these cells offer little or no potential for endometrial receptivity diagnostics.

## Data Availability

The data underlying this article are available in the article and its online Supplementary Material. The datasets are available in Gene Expression Omnibus and can be assessed with the unique identifier GSE221127.

## Supplementary material

**SUPPLEMENTARY FIGURE 1**

Uniform Manifold Approximation and Projection (UMAP) of transcriptome data of LH + 7 (receptive) and LH + 11 (post-receptive) cervical cells samples.

**SUPPLEMENTARY FIGURE 2**

Relative mRNA expression levels in LH + 7 and pooled group of *P* and LH + 2 cervical cells and endometrium samples. The error bars denote SEM (standard error of the mean), **P* value < 0.05, ***P* value < 0.01. Lower bars denote higher expression.

**SUPPLEMENTARY FIGURE 3**

Cellular composition of cervical cell samples collected throughout the menstrual cycle. The average cellular enrichment scores were calculated by xCell program.
