# Supplementary figures and images for: The cervical transcriptome changes during the menstrual cycle but does not predict the window of implantation
Source: Front Reprod Health. 2023 Jul 14;5:1224919. doi: 10.3389/frph.2023.1224919 (PMC10375708; doi:10.3389/frph.2023.1224919)

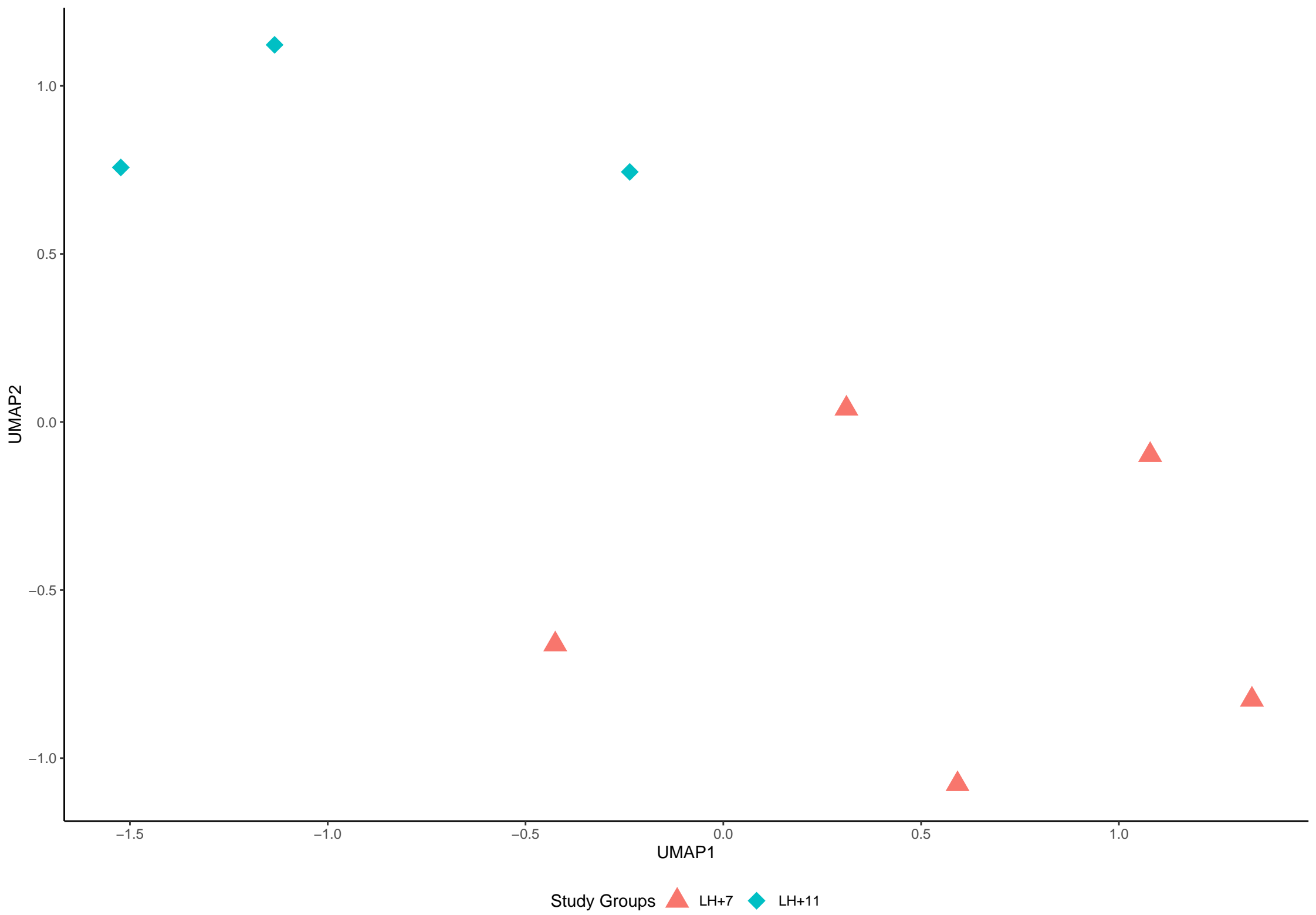

Supplement: Supplementary file 1 [file Image1.pdf]

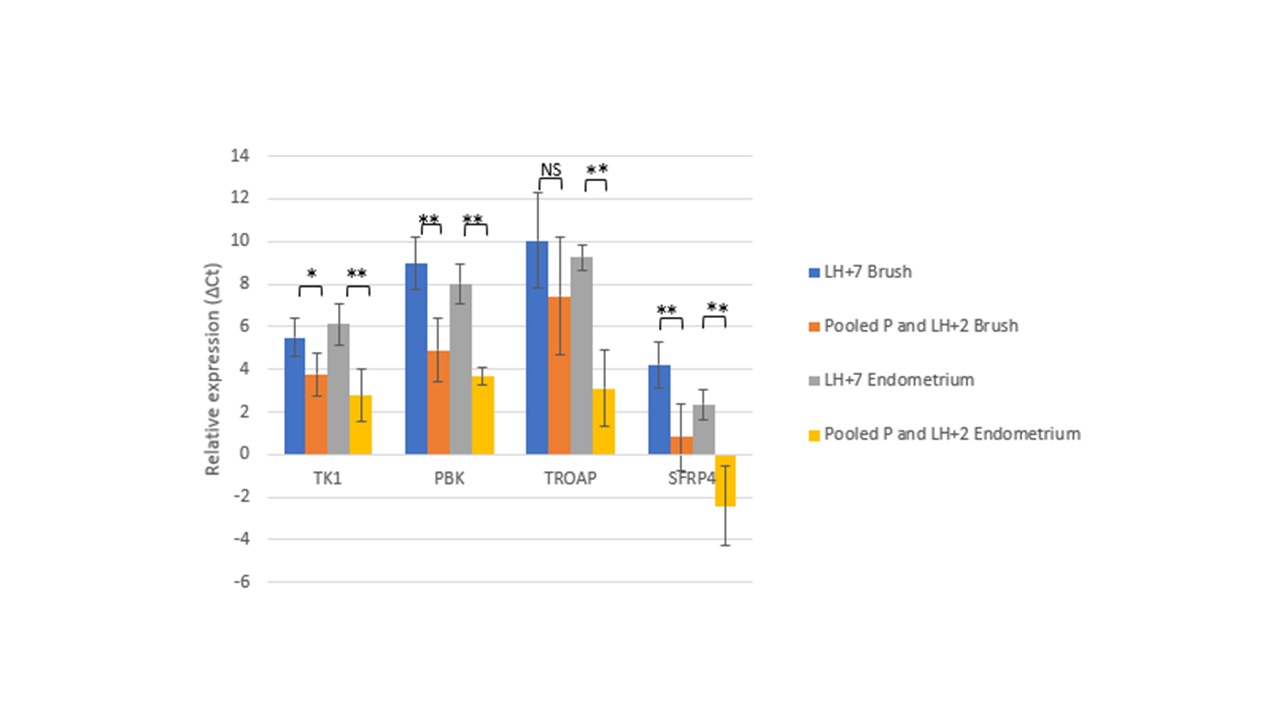

Supplement: Supplementary file 2 [file Image2.tif]

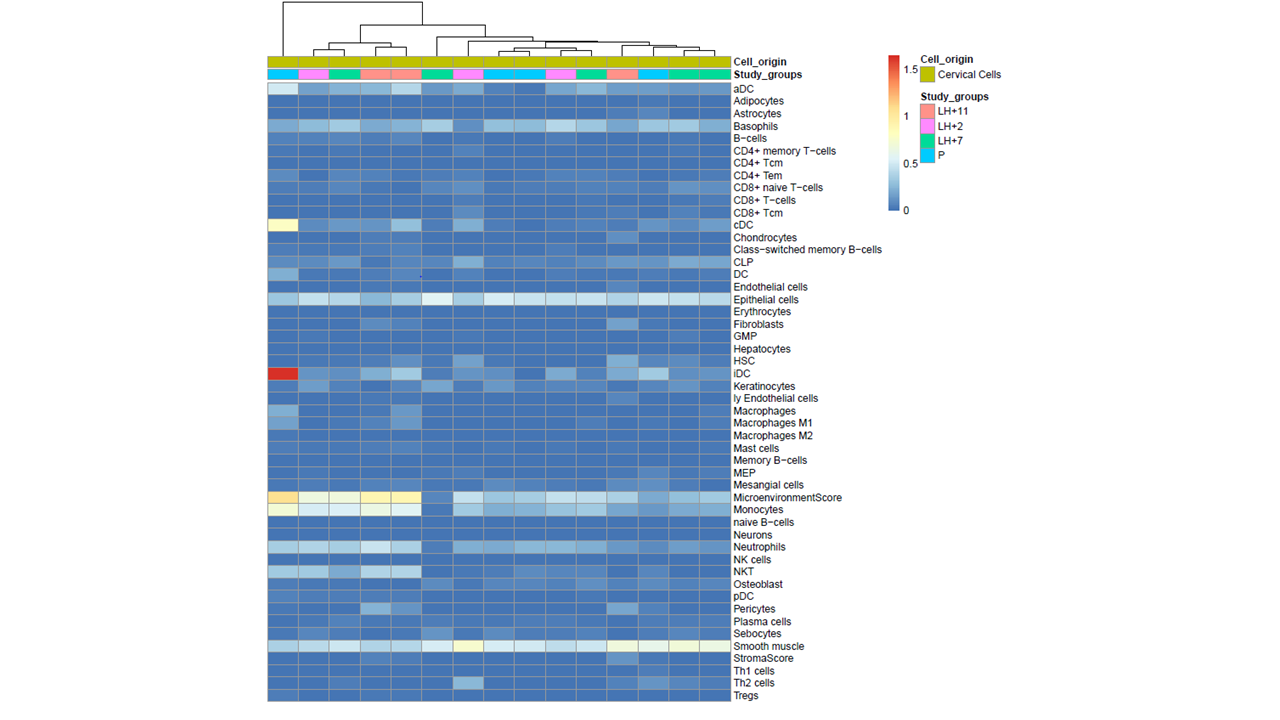

Supplement: Supplementary file 3 [file Image3.tif]
