# Supplementary material for: The cervical transcriptome changes during the menstrual cycle but does not predict the window of implantation
Source: Front Reprod Health. 2023 Jul 14;5:1224919. doi: 10.3389/frph.2023.1224919 (PMC10375708; doi:10.3389/frph.2023.1224919)
Supplement: Supplementary file 5 [file Table2.docx]

| **Supplementary table 2.** Differentially expressed genes (Log_2_Fold change > 1, FDR<0.01 and average values of transcript per million (TPM) per group) between proliferative (P) and LH+2 cervical cell samples of fertile women. | | | | |
| --- | --- | --- | --- | --- |
| **Gene_symbol** | **log2FC** | **FDR** | **TPM_P** | **TPM_LH+2** |
| SCGB1D4 | -7,52 | 3,38E-20 | 1,21 | 215,93 |
| SULT1E1 | -6,45 | 1,45E-06 | 0,35 | 12,10 |
| MYBPC1 | -5,22 | 1,55E-08 | 0,09 | 2,91 |
| SCGB1D2 | -5,07 | 9,07E-09 | 104,79 | 2692,98 |
| LINC00964 | -5,00 | 8,19E-03 | 0,08 | 2,42 |
| CXCL13 | -4,47 | 5,97E-19 | 0,90 | 17,07 |
| LEFTY1 | -4,39 | 8,19E-03 | 0,95 | 13,46 |
| SERPINA3 | -4,32 | 2,79E-07 | 59,91 | 935,72 |
| ADAMTS8 | -3,97 | 3,63E-04 | 0,31 | 3,31 |
| FGL1 | -3,96 | 3,30E-03 | 0,45 | 4,30 |
| AKR1C1 | -3,94 | 8,19E-03 | 1,87 | 17,37 |
| KRT24 | -3,90 | 6,20E-03 | 0,17 | 2,03 |
| SLC3A1 | -3,86 | 8,19E-03 | 0,78 | 6,76 |
| SERPINA5 | -3,85 | 3,29E-05 | 8,39 | 84,98 |
| ENPP3 | -3,66 | 9,06E-09 | 4,85 | 46,54 |
| FAM107A | -3,02 | 1,45E-04 | 1,13 | 7,30 |
| POU5F1 | -2,63 | 7,44E-03 | 6,48 | 32,29 |
| AC007681.1 | -2,34 | 5,63E-03 | 2,06 | 8,07 |
| MS4A1 | -2,26 | 4,68E-03 | 1,57 | 4,47 |
| ATAD2 | -2,23 | 6,95E-03 | 3,26 | 11,01 |
| GAS1 | -2,18 | 4,36E-03 | 2,25 | 8,81 |
| CEP70 | -1,94 | 5,13E-03 | 18,31 | 53,32 |
| LFNG | 1,81 | 3,30E-03 | 22,02 | 5,30 |
| ETV4 | 2,91 | 4,59E-05 | 6,49 | 0,67 |
| TFF2 | 4,25 | 5,63E-03 | 7,87 | 0,37 |
| SST | 4,94 | 8,90E-05 | 34,98 | 1,12 |
