# Supplementary material for: The cervical transcriptome changes during the menstrual cycle but does not predict the window of implantation
Source: Front Reprod Health. 2023 Jul 14;5:1224919. doi: 10.3389/frph.2023.1224919 (PMC10375708; doi:10.3389/frph.2023.1224919)
Supplement: Supplementary file 6 [file Table3.docx]

| **Supplementary table 3.** Differentially expressed genes (Log_2_Fold change > 1, FDR<0.01 and average values of transcript per million (TPM) per group) between LH+7 and pooled group of proliferative (P) and LH+2 samples. | | | | | |
| --- | --- | --- | --- | --- | --- |
| **Gene_symbol** | **log2FC** | **FDR** | **TPM_P** | **TPM_LH+2** | **TPM_LH+7** |
| MMP11 | -5,41 | 1,14E-06 | 171,08 | 29,20 | 2,25 |
| MMP3 | -5,05 | 7,38E-03 | 5,23 | 0,34 | 0,06 |
| NEFM | -4,31 | 9,30E-03 | 3,13 | 0,42 | 0,09 |
| PBK | -4,24 | 5,19E-04 | 2,65 | 4,84 | 0,28 |
| SFRP4 | -3,50 | 1,54E-03 | 161,17 | 63,24 | 10,76 |
| DLGAP5 | -3,39 | 8,99E-03 | 2,05 | 5,32 | 0,37 |
| KIF20A | -3,23 | 5,19E-04 | 2,57 | 3,06 | 0,37 |
| BUB1B | -2,98 | 1,99E-03 | 3,00 | 2,98 | 0,70 |
| NREP | -2,96 | 1,99E-03 | 84,32 | 30,44 | 5,83 |
| TOP2A | -2,94 | 5,07E-03 | 8,15 | 14,73 | 2,22 |
| HJURP | -2,89 | 1,99E-03 | 1,64 | 1,90 | 0,25 |
| CENPF | -2,85 | 1,39E-03 | 2,76 | 6,42 | 0,67 |
| FAM111B | -2,78 | 7,26E-03 | 2,05 | 2,67 | 0,32 |
| UCHL1 | -2,69 | 2,29E-03 | 6,56 | 3,25 | 0,75 |
| KIF23 | -2,64 | 5,07E-03 | 2,41 | 3,92 | 0,64 |
| TPX2 | -2,64 | 4,39E-04 | 2,85 | 2,35 | 0,41 |
| ADAM12 | -2,59 | 1,05E-04 | 2,68 | 1,03 | 0,22 |
| APOBEC3B | -2,58 | 1,99E-03 | 4,65 | 4,94 | 0,69 |
| UBE2C | -2,56 | 4,13E-03 | 11,18 | 6,65 | 1,51 |
| MIR503HG | -2,55 | 5,17E-03 | 11,06 | 6,17 | 1,39 |
| CEP55 | -2,53 | 5,07E-03 | 3,13 | 3,93 | 0,56 |
| KIFC1 | -2,48 | 1,39E-03 | 2,44 | 1,99 | 0,44 |
| CDC45 | -2,45 | 7,47E-03 | 2,47 | 1,22 | 0,74 |
| MELK | -2,41 | 9,40E-03 | 2,67 | 2,30 | 0,45 |
| BUB1 | -2,39 | 2,08E-03 | 2,02 | 2,28 | 0,38 |
| RRM2 | -2,32 | 1,05E-04 | 6,83 | 5,77 | 1,06 |
| CCNB2 | -2,30 | 7,46E-03 | 4,38 | 3,50 | 0,76 |
| MKI67 | -2,29 | 1,05E-04 | 1,63 | 1,98 | 0,47 |
| NEK2 | -2,18 | 5,55E-03 | 2,19 | 2,70 | 0,50 |
| KIF2C | -2,16 | 1,99E-03 | 2,21 | 2,92 | 0,59 |
| TROAP | -2,09 | 5,03E-03 | 2,20 | 2,59 | 0,55 |
| TYMS | -2,01 | 2,22E-03 | 11,80 | 8,42 | 2,57 |
| SHCBP1 | -1,90 | 4,16E-03 | 1,56 | 1,51 | 0,52 |
| TK1 | -1,52 | 5,07E-03 | 9,95 | 6,44 | 2,43 |
| FANCA | -1,44 | 3,75E-03 | 5,40 | 3,80 | 2,30 |
| MT1M | 4,01 | 5,03E-03 | 0,79 | 0,52 | 8,38 |
| MT1G | 4,27 | 3,83E-03 | 12,71 | 9,60 | 175,50 |
