# Supplementary material for: The cervical transcriptome changes during the menstrual cycle but does not predict the window of implantation
Source: Front Reprod Health. 2023 Jul 14;5:1224919. doi: 10.3389/frph.2023.1224919 (PMC10375708; doi:10.3389/frph.2023.1224919)
Supplement: Supplementary file 7 [file Table4.docx]

| **Supplementary table 4.** Differentially expressed genes (Log_2_Fold change > 1, FDR<0.01 and average values of transcript per million (TPM) per group) between LH+7 and LH+11 cervical cell samples of fertile women. | | | | |
| --- | --- | --- | --- | --- |
| **Gene Symbol** | **log2FC** | **FDR** | **TPM_LH+7** | **TPM_LH+11** |
| COMP | -9,35 | 5,05E-30 | 0,03 | 27,48 |
| AL513283.1 | -6,97 | 1,39E-08 | 0,02 | 3,91 |
| TMEM252 | -6,85 | 9,00E-11 | 0,18 | 32,20 |
| PAEP | -6,79 | 1,27E-20 | 16,62 | 2340,48 |
| AC011313.1 | -6,32 | 1,47E-04 | 0,00 | 3,06 |
| RHEBP2 | -6,21 | 6,89E-05 | 0,00 | 2,29 |
| CYP2C9 | -6,06 | 9,97E-08 | 0,14 | 13,00 |
| LINC02532 | -6,00 | 2,10E-12 | 0,25 | 27,51 |
| TEX101 | -5,88 | 2,15E-04 | 0,02 | 2,04 |
| AL117336.2 | -5,83 | 3,25E-04 | 0,34 | 17,56 |
| GALNT15 | -5,82 | 2,37E-10 | 0,07 | 3,35 |
| AL591846.1 | -5,71 | 3,16E-04 | 0,37 | 24,51 |
| TEK | -5,58 | 1,24E-15 | 0,07 | 4,48 |
| SORCS1 | -5,51 | 2,70E-06 | 0,05 | 2,10 |
| PLA2G2A | -5,51 | 3,25E-04 | 0,09 | 6,36 |
| TIMP3 | -5,27 | 1,87E-08 | 2,15 | 90,63 |
| GPAA1P1 | -5,25 | 7,70E-05 | 0,33 | 12,09 |
| SGIP1 | -5,23 | 2,07E-11 | 0,36 | 9,78 |
| NTRK2 | -5,20 | 2,65E-10 | 0,05 | 2,00 |
| AOX1 | -5,19 | 1,75E-14 | 0,55 | 11,14 |
| HNRNPA1P52 | -5,14 | 2,13E-03 | 0,06 | 2,42 |
| ADAMTS6 | -5,09 | 4,17E-12 | 0,09 | 3,84 |
| FXYD2 | -5,07 | 6,91E-10 | 0,61 | 24,29 |
| AC090954.1 | -4,99 | 1,59E-04 | 0,16 | 7,20 |
| ADAMTS5 | -4,98 | 3,84E-08 | 0,12 | 4,77 |
| PCDH17 | -4,95 | 6,40E-15 | 0,06 | 2,39 |
| DNAH17 | -4,86 | 1,46E-04 | 0,24 | 7,09 |
| ADCY1 | -4,82 | 3,95E-12 | 0,16 | 2,88 |
| ARSE | -4,77 | 1,30E-16 | 0,32 | 9,00 |
| CYS1 | -4,77 | 1,35E-26 | 0,18 | 5,59 |
| MMRN1 | -4,72 | 1,91E-07 | 0,26 | 10,12 |
| ACKR1 | -4,71 | 6,62E-11 | 0,71 | 23,09 |
| THBS2 | -4,70 | 4,82E-07 | 0,43 | 15,58 |
| MYH11 | -4,64 | 1,58E-07 | 0,07 | 2,16 |
| SAMMSON | -4,63 | 2,46E-04 | 0,11 | 2,93 |
| ADAMTS9 | -4,60 | 6,77E-09 | 1,76 | 59,21 |
| AC007728.3 | -4,59 | 9,38E-04 | 0,18 | 4,72 |
| DKK1 | -4,59 | 1,93E-06 | 7,00 | 209,71 |
| AC027288.3 | -4,52 | 1,62E-04 | 0,15 | 4,91 |
| SERPINE1 | -4,50 | 7,10E-11 | 0,19 | 5,40 |
| SLC18A2 | -4,50 | 5,14E-05 | 1,13 | 38,69 |
| SULF1 | -4,45 | 1,14E-08 | 0,71 | 14,47 |
| FAM239A | -4,45 | 2,88E-07 | 0,60 | 17,93 |
| ADAMTS2 | -4,44 | 2,11E-12 | 0,40 | 9,81 |
| EDNRB | -4,39 | 3,10E-09 | 0,43 | 11,20 |
| DOK5 | -4,39 | 1,47E-04 | 0,21 | 4,39 |
| CXCL12 | -4,37 | 1,87E-24 | 1,00 | 22,46 |
| AL391903.1 | -4,37 | 3,24E-03 | 1,15 | 27,45 |
| H19 | -4,37 | 2,55E-07 | 18,84 | 629,05 |
| KDR | -4,37 | 1,39E-08 | 0,28 | 6,56 |
| ABCA8 | -4,36 | 1,13E-05 | 0,38 | 7,86 |
| ABCC9 | -4,35 | 7,98E-04 | 0,12 | 3,06 |
| MMP11 | -4,33 | 1,91E-14 | 2,25 | 55,31 |
| PHYHIPL | -4,31 | 5,08E-04 | 0,81 | 21,28 |
| TRPC6 | -4,30 | 2,37E-07 | 0,31 | 6,38 |
| KIF5A | -4,27 | 3,73E-05 | 0,09 | 2,61 |
| ITGAD | -4,27 | 1,90E-08 | 0,14 | 4,11 |
| LMOD1 | -4,25 | 2,47E-11 | 0,36 | 9,04 |
| GNLY | -4,25 | 1,62E-08 | 15,30 | 369,83 |
| CD22 | -4,24 | 2,16E-04 | 1,88 | 30,95 |
| NID1 | -4,24 | 4,83E-08 | 1,50 | 37,24 |
| MYOCD | -4,21 | 2,42E-03 | 0,12 | 3,74 |
| RIPOR3 | -4,20 | 1,59E-13 | 0,32 | 6,64 |
| LBP | -4,20 | 3,25E-03 | 0,11 | 2,63 |
| HNF1A-AS1 | -4,08 | 2,50E-04 | 0,18 | 2,96 |
| MEG3 | -4,08 | 1,22E-08 | 8,14 | 85,17 |
| NCAM1 | -4,04 | 1,14E-13 | 0,25 | 3,02 |
| VCAN | -4,03 | 3,84E-08 | 14,13 | 282,51 |
| OLFML1 | -4,00 | 8,38E-07 | 0,39 | 7,33 |
| AC078950.1 | -3,99 | 4,61E-03 | 0,39 | 5,98 |
| FGF2 | -3,99 | 6,00E-05 | 0,13 | 2,47 |
| SPOCK1 | -3,98 | 2,59E-19 | 0,52 | 6,83 |
| CXorf36 | -3,98 | 2,89E-06 | 0,30 | 5,80 |
| PRDM8 | -3,96 | 3,72E-04 | 1,81 | 27,99 |
| ARHGAP28 | -3,94 | 2,82E-05 | 0,47 | 3,58 |
| TIE1 | -3,94 | 3,82E-07 | 0,26 | 4,16 |
| AC112496.1 | -3,93 | 8,91E-03 | 2,76 | 49,42 |
| C2CD4A | -3,92 | 4,46E-09 | 2,28 | 42,09 |
| HOXD10 | -3,92 | 2,77E-04 | 0,65 | 12,18 |
| COL4A1 | -3,91 | 3,66E-08 | 2,30 | 43,68 |
| HAND2 | -3,91 | 1,05E-03 | 1,65 | 29,82 |
| CPXM1 | -3,90 | 6,03E-10 | 2,73 | 48,17 |
| HAND2-AS1 | -3,89 | 7,65E-04 | 1,71 | 28,70 |
| CNTN1 | -3,89 | 1,84E-05 | 0,15 | 2,88 |
| CCL21 | -3,88 | 1,88E-08 | 2,82 | 58,64 |
| CDH2 | -3,84 | 1,32E-04 | 0,17 | 2,12 |
| PRAME | -3,82 | 1,59E-13 | 0,84 | 12,95 |
| GJC1 | -3,82 | 1,13E-03 | 0,17 | 2,66 |
| ABI3BP | -3,81 | 9,09E-05 | 1,19 | 14,58 |
| CNKSR2 | -3,80 | 4,63E-05 | 0,30 | 3,13 |
| ADAMTS12 | -3,80 | 1,05E-04 | 0,22 | 3,48 |
| NAALAD2 | -3,80 | 7,79E-04 | 0,61 | 11,06 |
| AC007278.1 | -3,80 | 2,63E-03 | 0,30 | 4,98 |
| ADAM12 | -3,78 | 1,32E-18 | 0,22 | 3,52 |
| DRAIC | -3,78 | 1,67E-05 | 0,27 | 3,52 |
| SYNPO2 | -3,77 | 5,33E-07 | 0,35 | 6,73 |
| ANGPTL2 | -3,77 | 1,91E-07 | 0,69 | 12,71 |
| DACT1 | -3,74 | 1,38E-04 | 0,41 | 6,79 |
| CXCL14 | -3,73 | 2,75E-06 | 16,30 | 285,70 |
| APOBEC3B | -3,72 | 2,52E-05 | 0,69 | 10,14 |
| GJA1 | -3,72 | 7,88E-05 | 8,08 | 109,72 |
| FBN1 | -3,72 | 2,18E-07 | 1,66 | 27,45 |
| LAMC3 | -3,70 | 6,52E-08 | 1,52 | 19,88 |
| COL5A1 | -3,68 | 3,17E-08 | 1,40 | 24,87 |
| MUM1L1 | -3,68 | 6,74E-04 | 1,38 | 26,90 |
| KCNJ8 | -3,68 | 1,54E-03 | 0,46 | 8,06 |
| CALD1 | -3,68 | 1,91E-14 | 3,90 | 58,98 |
| ADGRE2 | -3,66 | 3,88E-03 | 10,56 | 138,80 |
| MRGPRF | -3,65 | 6,55E-06 | 0,32 | 5,50 |
| KIAA1755 | -3,65 | 5,47E-14 | 0,22 | 2,41 |
| MAMDC2 | -3,63 | 7,88E-05 | 0,25 | 3,79 |
| FILIP1L | -3,63 | 3,45E-15 | 0,92 | 13,53 |
| THBS1 | -3,63 | 5,86E-03 | 8,50 | 94,96 |
| WT1-AS | -3,62 | 3,86E-03 | 0,29 | 4,98 |
| CD177 | -3,61 | 2,83E-03 | 1,84 | 32,46 |
| SVEP1 | -3,61 | 2,42E-06 | 0,16 | 2,52 |
| PTPRB | -3,60 | 6,60E-09 | 0,16 | 2,69 |
| AL359532.1 | -3,59 | 6,88E-04 | 0,14 | 1,98 |
| RASL11B | -3,59 | 7,15E-04 | 0,14 | 2,28 |
| KIR2DL4 | -3,58 | 3,24E-04 | 0,15 | 2,04 |
| TRPC4 | -3,58 | 2,96E-03 | 0,16 | 2,50 |
| THSD7A | -3,58 | 8,29E-08 | 0,53 | 7,03 |
| RHOJ | -3,58 | 1,19E-05 | 0,43 | 4,57 |
| TMCC3 | -3,57 | 2,82E-04 | 3,87 | 46,60 |
| PAPLN | -3,57 | 1,27E-08 | 4,00 | 51,94 |
| MCAM | -3,56 | 7,90E-04 | 0,68 | 7,72 |
| DIO3OS | -3,55 | 2,71E-14 | 2,88 | 17,03 |
| SLC15A1 | -3,55 | 1,02E-06 | 3,45 | 42,66 |
| P3H2 | -3,55 | 3,06E-18 | 0,85 | 11,41 |
| TBX3 | -3,54 | 7,01E-06 | 1,13 | 18,02 |
| ITGAX | -3,54 | 2,06E-03 | 26,34 | 399,99 |
| HOXD11 | -3,54 | 4,70E-05 | 0,34 | 5,12 |
| LUM | -3,53 | 1,08E-04 | 44,29 | 667,27 |
| NOX4 | -3,53 | 3,16E-06 | 0,69 | 9,93 |
| STON1 | -3,52 | 1,37E-05 | 0,62 | 7,91 |
| CRISPLD2 | -3,52 | 1,00E-06 | 2,30 | 30,24 |
| ADGRD1 | -3,52 | 1,63E-03 | 0,47 | 7,55 |
| COL3A1 | -3,52 | 1,59E-04 | 44,37 | 722,36 |
| CHSY1 | -3,51 | 2,33E-03 | 10,88 | 119,04 |
| NOS3 | -3,51 | 1,24E-06 | 0,27 | 2,80 |
| SPARC | -3,51 | 4,45E-05 | 42,18 | 653,13 |
| RUNX1T1 | -3,51 | 8,87E-07 | 0,28 | 2,89 |
| ACTA2 | -3,51 | 6,24E-11 | 6,15 | 96,09 |
| S1PR3 | -3,50 | 1,94E-05 | 0,55 | 8,43 |
| AC112229.1 | -3,50 | 1,41E-03 | 2,80 | 28,94 |
| HABP2 | -3,50 | 1,50E-08 | 2,35 | 27,79 |
| F2R | -3,49 | 2,03E-11 | 0,58 | 7,31 |
| FLT4 | -3,49 | 2,05E-06 | 0,17 | 1,92 |
| DCHS1 | -3,48 | 3,54E-10 | 0,28 | 3,66 |
| PRLR | -3,47 | 2,64E-06 | 0,77 | 7,94 |
| NOSTRIN | -3,45 | 1,44E-05 | 1,47 | 22,23 |
| AC136475.5 | -3,45 | 7,15E-03 | 1,41 | 18,35 |
| AL139393.2 | -3,44 | 7,11E-06 | 0,23 | 2,60 |
| ADGRF5 | -3,44 | 3,50E-04 | 0,28 | 3,73 |
| P2RY14 | -3,44 | 3,25E-04 | 1,36 | 16,30 |
| CLDN5 | -3,43 | 2,78E-05 | 0,25 | 3,27 |
| HAP1 | -3,43 | 4,10E-03 | 0,67 | 10,37 |
| PDGFRA | -3,42 | 3,73E-03 | 3,96 | 57,86 |
| CLMP | -3,42 | 6,12E-04 | 0,53 | 6,44 |
| COL5A2 | -3,41 | 4,42E-05 | 1,97 | 29,30 |
| COL4A2 | -3,41 | 5,51E-09 | 3,44 | 44,40 |
| MMP19 | -3,41 | 6,53E-07 | 3,78 | 42,93 |
| PAG1 | -3,41 | 7,74E-04 | 1,50 | 21,72 |
| WNT2 | -3,41 | 2,61E-04 | 0,48 | 5,95 |
| PDGFRB | -3,40 | 6,31E-08 | 3,00 | 31,32 |
| PLEKHH2 | -3,40 | 2,41E-05 | 2,02 | 31,77 |
| CSDC2 | -3,40 | 8,41E-10 | 0,27 | 3,54 |
| HTR2B | -3,40 | 4,62E-03 | 1,06 | 14,74 |
| AC112229.3 | -3,39 | 5,76E-05 | 2,43 | 28,27 |
| LAMB1 | -3,39 | 6,72E-05 | 17,48 | 245,56 |
| ADGRL4 | -3,38 | 1,99E-03 | 0,56 | 7,79 |
| AC136475.9 | -3,38 | 7,34E-03 | 1,49 | 17,63 |
| LHFPL6 | -3,38 | 1,85E-05 | 0,60 | 8,44 |
| TGFB2 | -3,37 | 8,35E-10 | 1,25 | 11,59 |
| LINC02432 | -3,37 | 1,81E-08 | 0,68 | 7,24 |
| SYT14 | -3,37 | 6,80E-03 | 0,19 | 2,17 |
| HEPH | -3,36 | 7,47E-05 | 0,49 | 7,61 |
| ECSCR | -3,36 | 2,91E-06 | 1,51 | 24,38 |
| CPZ | -3,36 | 1,50E-11 | 1,07 | 13,29 |
| KIR2DL3 | -3,35 | 3,95E-04 | 0,16 | 1,92 |
| RORB | -3,34 | 2,68E-04 | 1,01 | 14,91 |
| ALDH1A2 | -3,34 | 1,18E-08 | 4,90 | 58,07 |
| ENPEP | -3,33 | 2,87E-03 | 0,46 | 5,86 |
| FOXL2NB | -3,33 | 1,64E-04 | 0,36 | 4,58 |
| SEMA5A | -3,33 | 2,61E-06 | 0,69 | 9,19 |
| AC104695.3 | -3,32 | 1,24E-04 | 0,99 | 10,46 |
| ADAMTS1 | -3,31 | 7,02E-06 | 2,20 | 23,12 |
| CAMK1G | -3,31 | 8,26E-03 | 0,35 | 5,87 |
| FAM157C | -3,31 | 1,96E-03 | 8,90 | 80,45 |
| SFRP1 | -3,30 | 4,44E-04 | 2,33 | 29,29 |
| BMP6 | -3,30 | 8,86E-07 | 0,32 | 3,88 |
| SCARA5 | -3,30 | 1,94E-03 | 0,47 | 6,79 |
| VWF | -3,30 | 2,56E-09 | 0,36 | 4,22 |
| EBF1 | -3,29 | 2,29E-05 | 0,20 | 2,72 |
| JAM3 | -3,28 | 4,92E-06 | 0,52 | 6,43 |
| IL1RAP | -3,28 | 3,93E-03 | 10,71 | 95,43 |
| CD248 | -3,28 | 5,09E-05 | 1,47 | 20,21 |
| CREB5 | -3,27 | 7,44E-03 | 6,79 | 62,95 |
| TWIST1 | -3,27 | 1,30E-04 | 0,31 | 3,17 |
| IL1RL1 | -3,25 | 4,23E-03 | 0,23 | 1,98 |
| RAMP3 | -3,25 | 6,41E-07 | 0,79 | 8,40 |
| DGAT2 | -3,25 | 6,97E-03 | 13,77 | 156,74 |
| ADAM33 | -3,25 | 8,69E-07 | 1,96 | 21,48 |
| CDH13 | -3,25 | 1,87E-04 | 0,84 | 6,38 |
| TRPM8 | -3,25 | 1,11E-04 | 0,32 | 3,42 |
| AL078604.2 | -3,25 | 7,16E-03 | 0,54 | 6,21 |
| ECM2 | -3,25 | 1,66E-03 | 0,71 | 8,99 |
| SLC11A1 | -3,25 | 8,74E-03 | 35,48 | 381,69 |
| INHBA | -3,22 | 4,28E-03 | 1,59 | 16,31 |
| DEPDC1B | -3,21 | 1,00E-05 | 0,24 | 2,84 |
| AC123912.4 | -3,20 | 6,03E-03 | 0,59 | 6,68 |
| AC141928.1 | -3,20 | 2,92E-06 | 0,59 | 6,82 |
| THY1 | -3,19 | 1,69E-05 | 1,80 | 25,68 |
| NOD2 | -3,18 | 1,05E-03 | 2,74 | 25,84 |
| C1QTNF1 | -3,18 | 1,22E-10 | 0,77 | 6,75 |
| MX2 | -3,18 | 6,78E-03 | 14,22 | 154,12 |
| FLNA | -3,18 | 1,72E-07 | 44,27 | 399,23 |
| EMCN | -3,17 | 1,21E-05 | 0,79 | 10,64 |
| NAP1L4P1 | -3,17 | 2,53E-03 | 0,26 | 2,41 |
| MSRB3 | -3,17 | 4,84E-08 | 0,53 | 3,78 |
| SLC2A5 | -3,16 | 6,05E-05 | 1,07 | 10,11 |
| TMEM88 | -3,16 | 1,96E-03 | 1,70 | 15,90 |
| SFRP4 | -3,16 | 5,07E-04 | 10,76 | 126,35 |
| MOXD1 | -3,15 | 4,06E-03 | 1,73 | 22,82 |
| CLEC14A | -3,15 | 7,83E-05 | 0,30 | 3,29 |
| FLT1 | -3,14 | 7,23E-06 | 1,01 | 9,06 |
| NECAB2 | -3,13 | 8,28E-03 | 0,71 | 5,21 |
| PAX8-AS1 | -3,13 | 3,19E-04 | 1,12 | 9,62 |
| GPAA1P2 | -3,13 | 4,94E-08 | 2,60 | 22,06 |
| MFGE8 | -3,11 | 7,76E-06 | 6,69 | 74,00 |
| DCN | -3,11 | 6,67E-04 | 50,21 | 495,74 |
| NABP1 | -3,11 | 1,96E-03 | 50,99 | 405,70 |
| ATP13A3 | -3,10 | 2,61E-04 | 28,30 | 223,84 |
| ADAM23 | -3,10 | 9,98E-03 | 0,28 | 1,97 |
| COL1A1 | -3,09 | 4,66E-06 | 9,58 | 101,49 |
| EDNRA | -3,08 | 5,09E-03 | 1,13 | 14,23 |
| HELB | -3,08 | 7,34E-03 | 0,74 | 5,52 |
| CDH11 | -3,07 | 1,90E-06 | 2,82 | 17,87 |
| OSR2 | -3,07 | 5,36E-04 | 4,02 | 50,39 |
| ADAMTS15 | -3,06 | 4,43E-03 | 0,25 | 3,06 |
| PCOLCE | -3,06 | 1,57E-04 | 11,05 | 117,99 |
| BCL6B | -3,06 | 1,06E-05 | 0,19 | 2,20 |
| MMP2 | -3,05 | 1,36E-08 | 20,15 | 141,23 |
| PPP1R3B | -3,05 | 3,11E-03 | 10,98 | 97,40 |
| AL732372.3 | -3,04 | 2,02E-03 | 3,63 | 30,80 |
| EMILIN1 | -3,04 | 1,90E-06 | 1,64 | 16,03 |
| PYGM | -3,04 | 2,03E-03 | 0,42 | 4,10 |
| MFAP2 | -3,03 | 2,30E-08 | 1,82 | 19,38 |
| PCBP3 | -3,03 | 2,12E-04 | 0,22 | 2,50 |
| APCDD1 | -3,03 | 2,50E-03 | 8,01 | 85,86 |
| GPX3 | -3,02 | 4,30E-16 | 97,12 | 975,48 |
| SMTN | -3,02 | 9,49E-07 | 2,59 | 20,19 |
| MEX3B | -3,02 | 1,90E-08 | 0,27 | 2,41 |
| A2M | -3,01 | 8,39E-10 | 27,09 | 182,11 |
| ANO4 | -3,01 | 4,85E-03 | 0,42 | 4,14 |
| LILRB5 | -3,01 | 3,82E-04 | 0,26 | 1,83 |
| ISLR | -3,01 | 1,26E-06 | 7,92 | 84,16 |
| COL15A1 | -3,00 | 2,26E-08 | 1,02 | 9,80 |
| HOXD9 | -3,00 | 1,54E-03 | 0,74 | 6,69 |
| NES | -2,99 | 1,60E-03 | 0,61 | 6,65 |
| TAGLN | -2,99 | 4,03E-12 | 11,47 | 148,99 |
| TRAF3IP3 | -2,98 | 3,79E-04 | 7,75 | 58,87 |
| GFPT2 | -2,98 | 2,03E-06 | 0,62 | 5,43 |
| DIO3 | -2,98 | 5,92E-08 | 0,23 | 2,20 |
| BHMT2 | -2,97 | 1,54E-05 | 0,33 | 3,47 |
| TNXB | -2,97 | 4,14E-07 | 1,90 | 19,24 |
| AC138393.1 | -2,97 | 5,86E-03 | 0,61 | 3,19 |
| FAM89A | -2,96 | 7,70E-05 | 0,81 | 8,50 |
| TMEM200B | -2,96 | 2,77E-05 | 0,43 | 4,55 |
| VSTM4 | -2,96 | 1,02E-08 | 0,27 | 2,50 |
| CYFIP2 | -2,96 | 7,25E-04 | 5,38 | 51,63 |
| NRIP3 | -2,96 | 1,27E-03 | 0,97 | 6,80 |
| ITGA5 | -2,95 | 7,88E-05 | 5,66 | 55,19 |
| HS3ST3B1 | -2,95 | 1,51E-03 | 1,35 | 11,18 |
| JAM2 | -2,95 | 4,84E-08 | 0,33 | 2,99 |
| SLC26A10 | -2,95 | 1,56E-04 | 0,47 | 3,35 |
| COL6A2 | -2,95 | 1,17E-04 | 8,94 | 92,53 |
| SRPX | -2,94 | 8,73E-03 | 0,40 | 4,94 |
| PODN | -2,94 | 6,23E-06 | 0,48 | 4,69 |
| NCR1 | -2,94 | 6,93E-03 | 0,55 | 4,14 |
| TMEM140 | -2,94 | 3,60E-03 | 9,90 | 93,45 |
| ADAMTS7 | -2,93 | 1,11E-04 | 0,22 | 2,36 |
| CACNA1H | -2,93 | 4,45E-05 | 0,24 | 2,68 |
| ITGAM | -2,92 | 3,09E-03 | 5,75 | 49,83 |
| AL645608.1 | -2,91 | 2,29E-05 | 0,41 | 3,29 |
| B3GNT5 | -2,91 | 9,04E-03 | 12,26 | 87,97 |
| CPT1C | -2,90 | 5,96E-04 | 0,54 | 7,06 |
| MXRA8 | -2,90 | 9,05E-06 | 7,37 | 70,69 |
| IGF2 | -2,90 | 2,67E-05 | 3,67 | 36,91 |
| MASP1 | -2,89 | 8,82E-04 | 1,79 | 17,91 |
| EML1 | -2,88 | 7,23E-11 | 0,77 | 5,74 |
| PNRC1 | -2,88 | 8,46E-03 | 79,02 | 641,00 |
| COL6A3 | -2,87 | 6,48E-05 | 4,44 | 42,06 |
| AC013268.1 | -2,87 | 1,70E-03 | 4,43 | 41,49 |
| GABRP | -2,85 | 1,97E-03 | 29,30 | 279,72 |
| CNN1 | -2,85 | 9,95E-06 | 0,60 | 4,67 |
| FGF13 | -2,84 | 1,59E-03 | 0,47 | 2,16 |
| HOXD8 | -2,83 | 8,26E-03 | 0,40 | 3,30 |
| DOCK4 | -2,83 | 9,26E-04 | 3,43 | 27,69 |
| PRRX1 | -2,83 | 1,04E-04 | 2,02 | 19,20 |
| DPT | -2,82 | 5,47E-03 | 0,75 | 7,73 |
| GNG4 | -2,82 | 4,85E-03 | 0,69 | 3,50 |
| GPC2 | -2,82 | 6,95E-04 | 0,25 | 2,04 |
| PLIN4 | -2,81 | 8,00E-03 | 0,97 | 7,05 |
| ADCY5 | -2,81 | 1,86E-03 | 0,37 | 1,71 |
| MCC | -2,81 | 5,11E-03 | 1,01 | 5,78 |
| MAMLD1 | -2,80 | 2,40E-08 | 0,92 | 6,08 |
| TUBB3 | -2,80 | 4,36E-04 | 1,30 | 8,07 |
| PHLDB2 | -2,78 | 4,68E-03 | 0,87 | 5,26 |
| COLEC11 | -2,78 | 5,13E-03 | 0,33 | 2,30 |
| SLC1A1 | -2,78 | 1,26E-09 | 6,65 | 57,98 |
| ABCA1 | -2,77 | 6,85E-05 | 3,16 | 24,24 |
| ANTXR2 | -2,77 | 6,33E-04 | 13,46 | 108,51 |
| CACNA1C | -2,77 | 5,26E-03 | 0,38 | 1,66 |
| SEMA6A | -2,76 | 2,72E-03 | 0,39 | 1,95 |
| SP140 | -2,76 | 3,21E-03 | 3,09 | 16,25 |
| AC079944.2 | -2,76 | 2,55E-05 | 0,95 | 7,53 |
| SIRPB2 | -2,76 | 8,26E-03 | 4,62 | 32,40 |
| CLEC3B | -2,76 | 4,87E-03 | 4,30 | 45,22 |
| REM1 | -2,75 | 8,04E-03 | 0,48 | 4,36 |
| DOCK8 | -2,75 | 9,08E-03 | 7,02 | 52,62 |
| FREM1 | -2,75 | 8,71E-05 | 0,60 | 5,33 |
| LPL | -2,75 | 2,23E-03 | 0,70 | 5,02 |
| LSAMP | -2,74 | 4,33E-03 | 0,40 | 3,89 |
| OLFML3 | -2,74 | 1,74E-04 | 5,15 | 47,41 |
| SGTB | -2,74 | 7,07E-03 | 1,86 | 12,10 |
| PELI1 | -2,74 | 8,48E-03 | 16,87 | 125,54 |
| ADGRA2 | -2,74 | 1,32E-03 | 1,04 | 9,96 |
| PLAGL1 | -2,73 | 3,18E-05 | 4,35 | 27,43 |
| CYP3A5 | -2,73 | 5,56E-03 | 3,92 | 24,96 |
| S100A1 | -2,73 | 5,27E-03 | 3,18 | 28,21 |
| AL390066.1 | -2,73 | 8,73E-04 | 0,68 | 4,79 |
| CYP1B1 | -2,73 | 2,03E-06 | 0,53 | 4,05 |
| NREP | -2,73 | 1,43E-04 | 5,83 | 52,25 |
| ARID5A | -2,72 | 7,48E-04 | 8,62 | 60,21 |
| DNM1 | -2,72 | 7,29E-07 | 0,77 | 6,29 |
| SNX20 | -2,72 | 4,70E-03 | 3,53 | 26,29 |
| FBLN2 | -2,72 | 2,12E-06 | 4,12 | 29,63 |
| OLFML2B | -2,71 | 6,81E-12 | 2,53 | 15,56 |
| KIRREL1 | -2,71 | 2,98E-03 | 0,65 | 5,00 |
| PRDM1 | -2,71 | 5,47E-05 | 2,77 | 17,66 |
| PHLDB1 | -2,71 | 1,33E-05 | 4,97 | 41,13 |
| PRKDC | -2,71 | 5,15E-04 | 22,50 | 133,68 |
| GEM | -2,70 | 3,85E-05 | 1,11 | 6,35 |
| SAMD11 | -2,70 | 4,27E-06 | 0,99 | 8,79 |
| FOXL2 | -2,70 | 6,74E-04 | 0,31 | 2,64 |
| CD93 | -2,69 | 9,70E-03 | 5,68 | 41,66 |
| ADCY3 | -2,69 | 3,17E-12 | 4,61 | 37,50 |
| BCL2L10 | -2,69 | 2,16E-05 | 0,38 | 3,00 |
| ANK2 | -2,68 | 2,03E-03 | 0,47 | 2,57 |
| ZBTB16 | -2,66 | 6,99E-04 | 0,32 | 2,29 |
| LAMA2 | -2,66 | 8,00E-03 | 2,05 | 11,92 |
| HAL | -2,66 | 3,64E-03 | 3,14 | 28,36 |
| MIR503HG | -2,65 | 3,34E-05 | 1,39 | 11,94 |
| MRVI1 | -2,65 | 3,02E-03 | 1,50 | 12,18 |
| FGFR4 | -2,65 | 4,77E-04 | 0,20 | 2,13 |
| AEBP1 | -2,64 | 1,05E-07 | 4,78 | 35,72 |
| ENPP1 | -2,64 | 3,17E-03 | 0,61 | 5,36 |
| LDB2 | -2,64 | 1,54E-03 | 2,10 | 13,67 |
| FN1 | -2,64 | 1,43E-04 | 12,32 | 91,18 |
| ASPN | -2,63 | 1,63E-03 | 0,39 | 2,82 |
| POSTN | -2,63 | 7,36E-03 | 0,33 | 2,62 |
| DLC1 | -2,62 | 1,16E-04 | 0,36 | 2,96 |
| FHL1 | -2,62 | 2,35E-03 | 1,37 | 12,25 |
| CYYR1 | -2,62 | 2,71E-09 | 0,57 | 4,02 |
| CDH5 | -2,61 | 1,15E-03 | 0,59 | 3,80 |
| SLC7A11 | -2,60 | 2,57E-03 | 1,10 | 7,92 |
| LINC01001 | -2,59 | 8,66E-03 | 14,67 | 86,80 |
| MMRN2 | -2,59 | 7,90E-03 | 0,47 | 2,64 |
| RECK | -2,59 | 6,78E-07 | 0,41 | 2,23 |
| IL6R | -2,59 | 8,83E-03 | 9,02 | 62,99 |
| RN7SKP176 | -2,59 | 3,77E-04 | 0,79 | 4,51 |
| PLB1 | -2,58 | 1,83E-03 | 2,54 | 15,19 |
| DOCK5 | -2,58 | 4,85E-03 | 7,11 | 54,31 |
| HPN | -2,57 | 7,40E-03 | 0,83 | 5,50 |
| PROCR | -2,57 | 7,02E-06 | 1,77 | 14,16 |
| LMCD1 | -2,57 | 2,96E-05 | 1,13 | 8,23 |
| GNA13 | -2,56 | 4,31E-03 | 12,26 | 81,40 |
| COL14A1 | -2,56 | 6,12E-03 | 0,69 | 6,00 |
| HSPB6 | -2,55 | 2,65E-07 | 1,70 | 12,88 |
| CCDC71L | -2,55 | 1,37E-03 | 4,10 | 26,62 |
| C1QTNF7 | -2,55 | 2,52E-03 | 0,34 | 1,75 |
| LIMS1 | -2,55 | 7,11E-04 | 18,51 | 110,03 |
| TBX2 | -2,54 | 3,45E-03 | 1,28 | 6,76 |
| PDE2A | -2,54 | 3,26E-05 | 0,48 | 2,39 |
| FRY | -2,54 | 7,91E-03 | 2,50 | 12,58 |
| QKI | -2,53 | 5,18E-04 | 15,27 | 65,66 |
| MYC | -2,53 | 1,03E-04 | 2,81 | 19,52 |
| TGFB1I1 | -2,53 | 2,10E-06 | 1,67 | 11,91 |
| KLF6 | -2,52 | 1,12E-05 | 22,35 | 134,24 |
| ERN1 | -2,52 | 1,17E-03 | 4,81 | 24,99 |
| ATG2A | -2,51 | 8,34E-03 | 4,59 | 33,02 |
| DENND2A | -2,51 | 3,15E-04 | 0,43 | 3,05 |
| CRYAB | -2,51 | 6,06E-04 | 5,36 | 40,79 |
| TIMP2 | -2,51 | 2,20E-14 | 11,16 | 74,28 |
| MAF | -2,50 | 2,16E-11 | 1,71 | 8,75 |
| COLEC12 | -2,50 | 1,02E-06 | 0,40 | 2,81 |
| ARSB | -2,50 | 2,37E-17 | 1,31 | 8,78 |
| CTSW | -2,50 | 6,62E-05 | 3,11 | 20,15 |
| GTPBP1 | -2,49 | 6,32E-03 | 12,30 | 96,06 |
| STARD9 | -2,49 | 3,18E-07 | 0,31 | 2,41 |
| MARCH3 | -2,49 | 8,37E-03 | 1,21 | 7,91 |
| FAM131B | -2,48 | 3,09E-03 | 0,26 | 1,81 |
| CNTN4 | -2,48 | 1,36E-04 | 0,82 | 4,13 |
| SH3BP5 | -2,48 | 2,55E-03 | 18,21 | 99,74 |
| GAB3 | -2,48 | 3,83E-03 | 1,27 | 8,00 |
| FAM198B-AS1 | -2,47 | 3,11E-05 | 0,48 | 2,92 |
| RFTN1 | -2,47 | 5,73E-07 | 3,30 | 23,01 |
| AC132217.1 | -2,47 | 1,02E-05 | 40,82 | 295,01 |
| SERPINE2 | -2,47 | 5,44E-03 | 0,94 | 5,55 |
| AVPR1A | -2,47 | 1,03E-03 | 0,40 | 2,47 |
| SKIL | -2,47 | 4,83E-03 | 16,67 | 108,63 |
| ANTXR1 | -2,46 | 3,23E-05 | 5,12 | 29,62 |
| RAB3IL1 | -2,46 | 1,66E-08 | 1,48 | 10,40 |
| IL10RA | -2,45 | 9,99E-03 | 7,67 | 48,21 |
| NEXN | -2,44 | 1,25E-04 | 0,42 | 1,92 |
| MITF | -2,44 | 5,22E-09 | 3,80 | 28,57 |
| CAVIN2 | -2,44 | 4,80E-03 | 0,36 | 2,58 |
| BMP1 | -2,44 | 6,51E-05 | 2,23 | 13,49 |
| KLHL15 | -2,43 | 9,24E-03 | 1,59 | 9,31 |
| AC138035.1 | -2,43 | 8,99E-03 | 15,43 | 93,07 |
| ARHGEF25 | -2,43 | 9,25E-04 | 1,35 | 9,35 |
| CRMP1 | -2,43 | 3,03E-03 | 0,36 | 2,33 |
| EDN1 | -2,43 | 6,29E-05 | 1,37 | 9,28 |
| SVIL | -2,42 | 1,62E-03 | 3,70 | 23,45 |
| AL133330.1 | -2,42 | 4,17E-03 | 0,94 | 5,93 |
| DAB2 | -2,42 | 9,72E-10 | 6,07 | 40,33 |
| ENG | -2,42 | 6,00E-09 | 4,30 | 26,18 |
| ELOVL7 | -2,42 | 2,11E-12 | 1,06 | 5,93 |
| ABCB1 | -2,42 | 1,92E-03 | 1,05 | 5,87 |
| PAPSS2 | -2,42 | 5,36E-04 | 3,76 | 17,39 |
| LEF1 | -2,41 | 4,25E-03 | 1,93 | 12,82 |
| TMEM158 | -2,41 | 1,44E-07 | 0,55 | 3,62 |
| SYT11 | -2,41 | 8,41E-08 | 0,78 | 5,01 |
| E2F3 | -2,41 | 6,71E-03 | 3,03 | 18,37 |
| IL2RB | -2,40 | 9,64E-09 | 1,73 | 10,73 |
| DPP4 | -2,40 | 8,05E-09 | 10,02 | 62,90 |
| IL6ST | -2,39 | 5,50E-10 | 28,18 | 153,89 |
| GADD45A | -2,39 | 6,27E-04 | 12,05 | 63,60 |
| GGT5 | -2,39 | 9,55E-04 | 1,12 | 4,63 |
| CDYL2 | -2,39 | 6,47E-10 | 1,52 | 9,31 |
| ADAMTS10 | -2,38 | 1,11E-06 | 1,96 | 10,84 |
| ZDHHC14 | -2,38 | 3,46E-06 | 1,15 | 5,67 |
| DTX1 | -2,38 | 1,48E-03 | 0,93 | 7,33 |
| TRANK1 | -2,37 | 2,48E-04 | 6,29 | 35,47 |
| GNAI1 | -2,37 | 7,30E-05 | 3,11 | 18,75 |
| PID1 | -2,37 | 9,95E-07 | 1,78 | 10,93 |
| HPSE | -2,37 | 5,88E-03 | 6,54 | 39,54 |
| GPC6 | -2,37 | 2,25E-03 | 0,26 | 1,79 |
| EMILIN2 | -2,36 | 2,47E-04 | 6,94 | 33,43 |
| MSC-AS1 | -2,36 | 4,57E-03 | 0,33 | 1,89 |
| TLN2 | -2,35 | 3,24E-05 | 0,44 | 3,49 |
| CHST15 | -2,35 | 3,87E-03 | 11,05 | 60,48 |
| TMEM255B | -2,35 | 2,64E-04 | 0,54 | 2,97 |
| CALHM2 | -2,35 | 2,01E-07 | 1,88 | 11,90 |
| PDLIM7 | -2,35 | 1,05E-03 | 13,13 | 86,34 |
| HECA | -2,35 | 2,64E-03 | 5,26 | 29,86 |
| BGN | -2,35 | 2,70E-03 | 2,60 | 15,82 |
| MIAT | -2,34 | 8,95E-04 | 0,79 | 3,56 |
| HIVEP2 | -2,34 | 4,65E-03 | 5,32 | 28,73 |
| NDEL1 | -2,34 | 9,40E-03 | 32,19 | 199,99 |
| TSHZ3 | -2,34 | 5,65E-06 | 0,96 | 5,71 |
| AOC3 | -2,34 | 3,67E-04 | 0,80 | 3,67 |
| RAMP2 | -2,34 | 2,65E-03 | 2,69 | 19,56 |
| CDO1 | -2,33 | 6,69E-04 | 0,30 | 1,95 |
| TSPAN5 | -2,33 | 2,46E-03 | 1,44 | 7,96 |
| FXYD6 | -2,32 | 2,43E-04 | 1,80 | 10,41 |
| MFAP4 | -2,32 | 5,88E-06 | 1,67 | 9,83 |
| UBE2B | -2,31 | 9,84E-03 | 48,68 | 309,17 |
| AKT3 | -2,31 | 4,68E-07 | 1,15 | 6,46 |
| MICAL1 | -2,31 | 1,44E-08 | 6,69 | 40,63 |
| TPST1 | -2,31 | 7,36E-07 | 4,68 | 27,28 |
| DPYSL3 | -2,31 | 6,99E-04 | 0,70 | 4,16 |
| FOSL2 | -2,30 | 7,21E-03 | 39,97 | 217,91 |
| IL3RA | -2,30 | 4,50E-04 | 1,99 | 10,13 |
| AXL | -2,30 | 9,49E-07 | 1,91 | 10,80 |
| NRP1 | -2,30 | 5,81E-04 | 5,02 | 23,99 |
| SULT1C4 | -2,29 | 2,06E-05 | 0,75 | 4,46 |
| GRK5 | -2,29 | 3,37E-05 | 1,27 | 7,51 |
| JARID2 | -2,29 | 4,19E-03 | 2,88 | 15,52 |
| ZYX | -2,29 | 2,54E-03 | 33,14 | 184,77 |
| HJURP | -2,29 | 3,24E-04 | 0,25 | 2,08 |
| KREMEN1 | -2,29 | 5,74E-04 | 3,79 | 19,65 |
| PDPN | -2,28 | 7,90E-04 | 4,17 | 20,93 |
| IL15 | -2,28 | 3,66E-05 | 5,54 | 26,69 |
| TNFRSF1A | -2,28 | 7,44E-03 | 52,53 | 285,43 |
| C1S | -2,28 | 2,62E-04 | 34,88 | 221,08 |
| ERG | -2,28 | 9,40E-08 | 0,47 | 2,48 |
| DPYSL2 | -2,27 | 1,04E-10 | 2,66 | 14,74 |
| PRKCA | -2,27 | 3,15E-06 | 0,78 | 4,31 |
| PLA2G4C | -2,27 | 2,43E-08 | 1,65 | 7,93 |
| LINC00654 | -2,27 | 2,09E-04 | 0,50 | 2,63 |
| ELF1 | -2,27 | 2,81E-03 | 16,39 | 83,59 |
| ARHGAP31 | -2,27 | 1,83E-03 | 1,15 | 4,63 |
| SCUBE2 | -2,27 | 4,33E-03 | 1,72 | 9,69 |
| KLRC1 | -2,26 | 1,08E-03 | 1,15 | 7,12 |
| ERI1 | -2,26 | 9,50E-03 | 6,80 | 27,68 |
| MYD88 | -2,25 | 9,05E-03 | 27,21 | 145,76 |
| ABLIM3 | -2,25 | 4,19E-03 | 1,29 | 6,48 |
| NR2F1-AS1 | -2,25 | 3,57E-03 | 0,92 | 6,21 |
| SLC8A1 | -2,25 | 8,46E-03 | 2,21 | 11,07 |
| CNRIP1 | -2,24 | 1,19E-04 | 1,51 | 9,70 |
| KANK2 | -2,24 | 1,82E-07 | 6,88 | 43,58 |
| MEF2C | -2,24 | 2,29E-04 | 1,79 | 9,74 |
| SERPINF1 | -2,24 | 6,19E-04 | 41,34 | 235,94 |
| GGT3P | -2,24 | 4,55E-03 | 0,78 | 4,72 |
| WDFY3 | -2,24 | 6,62E-04 | 8,62 | 44,31 |
| PGM5 | -2,24 | 4,79E-05 | 0,60 | 3,19 |
| C1R | -2,23 | 4,17E-04 | 64,76 | 362,06 |
| SCG5 | -2,23 | 5,83E-03 | 0,48 | 3,01 |
| AMIGO2 | -2,23 | 1,39E-05 | 4,45 | 22,97 |
| CMTM1 | -2,23 | 5,06E-03 | 1,56 | 6,61 |
| TGFBI | -2,23 | 2,72E-05 | 33,65 | 156,65 |
| CHST2 | -2,22 | 7,01E-06 | 1,50 | 7,76 |
| HIC1 | -2,22 | 1,20E-05 | 0,64 | 3,80 |
| HSPA2 | -2,22 | 3,26E-03 | 1,76 | 9,00 |
| AGPAT5 | -2,22 | 1,05E-04 | 4,96 | 25,36 |
| B3GNTL1 | -2,22 | 2,56E-03 | 4,70 | 18,83 |
| NUP98 | -2,22 | 5,23E-03 | 19,59 | 105,35 |
| RYBP | -2,21 | 1,42E-03 | 4,28 | 22,02 |
| CSGALNACT2 | -2,21 | 2,39E-03 | 5,91 | 31,97 |
| PLCG2 | -2,21 | 7,44E-03 | 6,93 | 37,23 |
| NLGN4X | -2,21 | 1,05E-03 | 1,60 | 9,02 |
| KCNAB3 | -2,20 | 2,96E-05 | 0,79 | 4,43 |
| VEGFA | -2,20 | 5,45E-03 | 40,91 | 211,37 |
| ADAM19 | -2,20 | 7,36E-03 | 1,86 | 9,62 |
| CCND2 | -2,20 | 4,49E-09 | 1,62 | 8,56 |
| WNT4 | -2,19 | 4,96E-04 | 2,69 | 15,16 |
| MCOLN1 | -2,19 | 2,57E-03 | 7,57 | 38,67 |
| ABCC3 | -2,19 | 6,24E-06 | 6,33 | 28,56 |
| AL353625.1 | -2,19 | 2,09E-03 | 0,66 | 2,89 |
| PGS1 | -2,18 | 8,74E-03 | 14,70 | 70,13 |
| CARD8 | -2,18 | 6,24E-03 | 17,41 | 76,82 |
| MED13L | -2,18 | 6,18E-04 | 6,99 | 35,16 |
| CLIP3 | -2,18 | 9,42E-05 | 0,80 | 4,47 |
| AL627309.1 | -2,18 | 4,90E-03 | 0,79 | 4,54 |
| CCM2L | -2,18 | 3,25E-03 | 0,53 | 2,89 |
| ZNF646 | -2,18 | 4,27E-03 | 2,70 | 12,99 |
| GPSM1 | -2,18 | 2,15E-04 | 2,41 | 12,13 |
| IGFBP2 | -2,17 | 7,54E-03 | 27,51 | 171,18 |
| IL18BP | -2,17 | 1,54E-03 | 3,81 | 19,73 |
| MAP4K4 | -2,17 | 3,36E-03 | 16,07 | 97,29 |
| WLS | -2,17 | 1,74E-03 | 12,62 | 62,70 |
| TUB | -2,16 | 7,02E-04 | 1,09 | 5,94 |
| YPEL4 | -2,16 | 6,63E-04 | 0,49 | 2,26 |
| GPBAR1 | -2,16 | 3,12E-03 | 0,36 | 1,94 |
| MAN1A1 | -2,16 | 2,05E-05 | 4,18 | 20,97 |
| PLEKHG2 | -2,16 | 3,80E-05 | 6,24 | 22,70 |
| MYCT1 | -2,16 | 1,80E-04 | 0,50 | 2,67 |
| SFMBT2 | -2,15 | 7,18E-03 | 1,05 | 5,47 |
| FBLN5 | -2,15 | 1,19E-05 | 8,28 | 47,08 |
| RAB21 | -2,15 | 4,29E-03 | 15,57 | 86,51 |
| HEY2 | -2,15 | 1,41E-03 | 0,45 | 2,56 |
| BOD1L1 | -2,15 | 3,64E-03 | 14,33 | 59,36 |
| CASC15 | -2,15 | 4,45E-05 | 0,44 | 2,06 |
| AKAP13 | -2,14 | 7,24E-06 | 19,13 | 81,26 |
| SPATA13 | -2,14 | 5,81E-03 | 9,55 | 44,57 |
| PMP22 | -2,14 | 1,01E-03 | 9,45 | 48,56 |
| CBL | -2,14 | 7,19E-03 | 2,40 | 11,41 |
| TYRO3 | -2,13 | 6,71E-03 | 0,81 | 4,07 |
| HGF | -2,13 | 8,60E-03 | 0,53 | 2,50 |
| HECW2 | -2,13 | 3,78E-03 | 0,76 | 2,14 |
| NRXN2 | -2,13 | 6,93E-04 | 0,56 | 2,70 |
| ADAMTSL4 | -2,13 | 2,36E-07 | 2,90 | 15,94 |
| AC093010.3 | -2,13 | 3,98E-04 | 4,78 | 27,42 |
| SPAG9 | -2,12 | 6,32E-03 | 18,41 | 114,96 |
| TPX2 | -2,12 | 2,11E-03 | 0,41 | 2,04 |
| COL6A1 | -2,12 | 1,30E-03 | 19,89 | 116,59 |
| NPR1 | -2,12 | 1,58E-03 | 1,34 | 7,83 |
| FAM171A2 | -2,11 | 1,67E-03 | 0,54 | 3,26 |
| C11orf96 | -2,11 | 1,05E-03 | 1,04 | 4,75 |
| IRS2 | -2,11 | 7,55E-03 | 4,15 | 17,28 |
| PTPN7 | -2,11 | 8,96E-03 | 2,55 | 12,52 |
| DLL1 | -2,11 | 3,46E-08 | 1,01 | 5,03 |
| ZSWIM6 | -2,10 | 8,10E-03 | 4,64 | 21,01 |
| IGFBP7 | -2,10 | 8,26E-06 | 79,83 | 436,80 |
| RGS16 | -2,10 | 2,93E-04 | 1,14 | 5,52 |
| KCNAB2 | -2,10 | 6,48E-03 | 4,52 | 22,61 |
| ELL2 | -2,10 | 9,37E-03 | 14,39 | 64,63 |
| S1PR1 | -2,09 | 1,67E-05 | 0,66 | 2,61 |
| EMID1 | -2,09 | 9,16E-04 | 0,90 | 5,10 |
| MSN | -2,09 | 4,28E-03 | 45,07 | 207,56 |
| FMNL3 | -2,09 | 2,83E-03 | 2,17 | 11,26 |
| ENPP2 | -2,08 | 2,09E-05 | 4,57 | 23,54 |
| HPS5 | -2,08 | 6,00E-03 | 5,22 | 27,32 |
| C6orf141 | -2,08 | 2,89E-03 | 4,21 | 22,87 |
| IL12RB1 | -2,07 | 9,67E-03 | 0,95 | 4,58 |
| LOXL2 | -2,06 | 3,80E-03 | 1,56 | 6,73 |
| ADRA2C | -2,06 | 8,06E-03 | 4,02 | 22,76 |
| MICB | -2,06 | 1,74E-04 | 1,05 | 4,72 |
| PARD6G | -2,05 | 5,29E-03 | 0,43 | 1,78 |
| SIPA1L2 | -2,05 | 6,43E-04 | 4,39 | 17,19 |
| WASF1 | -2,05 | 3,64E-03 | 0,68 | 2,70 |
| FBXO10 | -2,05 | 7,28E-04 | 0,74 | 2,93 |
| GSDME | -2,04 | 2,27E-05 | 4,25 | 19,65 |
| PCNX1 | -2,04 | 2,89E-03 | 10,14 | 39,16 |
| ID4 | -2,04 | 1,61E-06 | 13,25 | 66,81 |
| MKNK1 | -2,04 | 1,31E-03 | 17,64 | 82,12 |
| KCTD20 | -2,04 | 6,32E-03 | 10,59 | 47,46 |
| CMKLR1 | -2,04 | 8,74E-04 | 0,89 | 3,91 |
| NRM | -2,04 | 5,42E-07 | 1,85 | 9,77 |
| MKI67 | -2,04 | 1,90E-04 | 0,47 | 1,90 |
| BRWD3 | -2,03 | 8,78E-03 | 3,53 | 13,17 |
| VIPR2 | -2,03 | 3,38E-03 | 0,43 | 2,14 |
| TRO | -2,03 | 3,31E-04 | 3,38 | 15,17 |
| TMSB15B | -2,03 | 2,40E-03 | 1,23 | 5,88 |
| TGM3 | -2,03 | 7,18E-03 | 1,83 | 8,30 |
| TET3 | -2,03 | 5,11E-03 | 2,36 | 10,66 |
| SGK2 | -2,03 | 2,53E-03 | 0,63 | 2,91 |
| FNDC3B | -2,02 | 3,70E-03 | 17,57 | 74,39 |
| RABGEF1 | -2,02 | 8,48E-03 | 9,88 | 49,67 |
| TP53INP2 | -2,02 | 4,46E-03 | 5,93 | 25,96 |
| NKG7 | -2,02 | 3,55E-06 | 9,35 | 45,86 |
| TRERF1 | -2,01 | 1,84E-03 | 0,97 | 4,25 |
| ZMIZ1 | -2,01 | 1,80E-03 | 7,26 | 33,96 |
| FKBP10 | -2,01 | 1,23E-04 | 6,56 | 32,88 |
| LHFPL2 | -2,01 | 2,78E-05 | 4,51 | 20,25 |
| LDLRAD3 | -2,01 | 3,93E-03 | 1,00 | 4,17 |
| KIF20A | -2,00 | 9,98E-03 | 0,37 | 1,79 |
| ACAP2 | -2,00 | 1,00E-02 | 7,32 | 31,63 |
| SH2D3C | -2,00 | 2,06E-03 | 1,89 | 9,83 |
| ZCCHC24 | -2,00 | 2,25E-05 | 1,76 | 9,09 |
| ABAT | -2,00 | 1,44E-07 | 1,94 | 9,08 |
| sept.05 | -2,00 | 2,32E-04 | 2,13 | 10,97 |
| MAP1B | -2,00 | 3,55E-03 | 0,52 | 2,72 |
| GRB10 | -2,00 | 3,18E-05 | 2,09 | 9,65 |
| CPED1 | -1,99 | 4,70E-04 | 1,34 | 4,89 |
| EPB41L3 | -1,99 | 7,00E-03 | 8,01 | 38,85 |
| MAPK12 | -1,99 | 5,94E-03 | 0,87 | 4,68 |
| CD68 | -1,99 | 8,73E-03 | 44,21 | 212,99 |
| MYH9 | -1,99 | 2,23E-03 | 29,60 | 127,00 |
| NLGN2 | -1,99 | 3,19E-04 | 2,23 | 12,17 |
| EAF1 | -1,98 | 2,02E-03 | 4,63 | 20,33 |
| MYL9 | -1,98 | 1,75E-05 | 18,11 | 81,55 |
| SLC9B2 | -1,98 | 1,05E-03 | 1,06 | 4,34 |
| GDF11 | -1,98 | 2,09E-03 | 0,90 | 4,14 |
| FOLR2 | -1,98 | 5,13E-05 | 2,14 | 9,15 |
| AL121603.2 | -1,98 | 9,90E-03 | 0,67 | 3,02 |
| NOVA1 | -1,97 | 6,37E-03 | 0,84 | 2,07 |
| GAS7 | -1,97 | 3,91E-03 | 2,63 | 10,89 |
| FLCN | -1,96 | 1,34E-03 | 8,34 | 40,09 |
| KCTD12 | -1,96 | 9,29E-06 | 3,25 | 14,66 |
| SLIT3 | -1,96 | 3,29E-03 | 0,52 | 2,92 |
| PXDN | -1,95 | 8,03E-03 | 3,72 | 14,52 |
| WNT5A | -1,95 | 4,31E-03 | 3,93 | 17,86 |
| KAT6A | -1,95 | 2,42E-03 | 5,87 | 24,73 |
| GRASP | -1,94 | 1,71E-05 | 1,71 | 7,41 |
| AP3B2 | -1,94 | 5,95E-03 | 0,44 | 1,67 |
| CENPF | -1,94 | 2,82E-05 | 0,67 | 2,55 |
| PEG3 | -1,94 | 7,70E-05 | 1,25 | 5,58 |
| DIP2B | -1,94 | 4,58E-03 | 3,81 | 15,54 |
| BRAF | -1,94 | 5,44E-03 | 6,37 | 29,73 |
| CMTM3 | -1,94 | 1,40E-12 | 7,30 | 32,02 |
| NCOA1 | -1,94 | 7,20E-03 | 6,86 | 28,77 |
| CYTH3 | -1,94 | 1,00E-06 | 1,18 | 5,74 |
| JUN | -1,93 | 5,38E-04 | 6,99 | 32,38 |
| TWIST2 | -1,93 | 6,97E-03 | 0,72 | 3,56 |
| ZNF697 | -1,93 | 1,59E-03 | 0,67 | 2,95 |
| ARPC5 | -1,93 | 5,65E-03 | 56,07 | 240,00 |
| MYO9B | -1,93 | 5,14E-03 | 19,25 | 81,58 |
| XYLT1 | -1,92 | 4,66E-05 | 0,73 | 3,13 |
| SMG1P1 | -1,92 | 1,05E-03 | 2,57 | 10,23 |
| PRKD1 | -1,92 | 7,65E-03 | 0,79 | 4,13 |
| APBB2 | -1,92 | 1,81E-06 | 2,62 | 8,36 |
| BTG1 | -1,92 | 4,44E-03 | 39,31 | 158,13 |
| NCOA2 | -1,92 | 2,53E-03 | 4,19 | 15,92 |
| MTF1 | -1,92 | 5,44E-03 | 4,61 | 20,69 |
| ZNFX1 | -1,92 | 8,00E-03 | 7,16 | 29,39 |
| TMEFF1 | -1,92 | 1,05E-05 | 0,54 | 2,53 |
| PAMR1 | -1,92 | 1,33E-03 | 7,90 | 38,40 |
| RAMP1 | -1,91 | 2,93E-04 | 15,46 | 77,39 |
| GPNMB | -1,91 | 5,42E-03 | 43,98 | 185,51 |
| EFEMP1 | -1,91 | 4,94E-03 | 10,94 | 45,42 |
| GPR137B | -1,91 | 3,71E-04 | 6,50 | 26,48 |
| RBP5 | -1,91 | 2,78E-03 | 0,51 | 2,55 |
| RPS6KA5 | -1,91 | 4,94E-03 | 3,36 | 14,74 |
| BCL2 | -1,90 | 2,79E-14 | 0,94 | 3,96 |
| NEDD9 | -1,90 | 8,00E-03 | 14,96 | 70,36 |
| SYNJ1 | -1,90 | 7,20E-03 | 2,10 | 9,30 |
| SULF2 | -1,89 | 2,22E-10 | 27,49 | 117,35 |
| PXK | -1,89 | 5,65E-06 | 6,38 | 25,48 |
| PLPP3 | -1,89 | 3,72E-04 | 12,59 | 57,71 |
| MMP14 | -1,89 | 4,66E-05 | 25,71 | 101,23 |
| ARHGEF2 | -1,88 | 6,80E-03 | 15,73 | 60,14 |
| CAVIN1 | -1,88 | 6,34E-05 | 5,42 | 24,62 |
| MEX3A | -1,88 | 9,26E-04 | 0,46 | 2,01 |
| TLE4 | -1,88 | 6,67E-04 | 4,60 | 20,20 |
| MAST2 | -1,88 | 8,36E-05 | 4,21 | 17,20 |
| FHL3 | -1,87 | 1,27E-04 | 2,15 | 9,59 |
| APOE | -1,87 | 1,67E-03 | 40,28 | 193,93 |
| TMEM131L | -1,87 | 3,24E-03 | 1,24 | 4,73 |
| SLC36A4 | -1,87 | 4,67E-03 | 2,84 | 12,57 |
| LEPR | -1,87 | 5,78E-05 | 2,26 | 9,57 |
| UTRN | -1,86 | 5,91E-08 | 7,10 | 23,85 |
| FNIP2 | -1,86 | 2,65E-03 | 4,10 | 16,57 |
| SLC15A4 | -1,86 | 1,12E-03 | 12,05 | 51,11 |
| SYDE1 | -1,86 | 9,48E-04 | 0,76 | 3,56 |
| AP1S2 | -1,86 | 7,84E-04 | 10,19 | 39,10 |
| FAM151B | -1,85 | 4,69E-03 | 0,96 | 3,85 |
| LATS2 | -1,85 | 1,01E-03 | 1,64 | 6,11 |
| TCF4 | -1,85 | 2,50E-06 | 4,01 | 15,84 |
| CLIC4 | -1,85 | 1,78E-07 | 15,35 | 62,99 |
| CLEC1A | -1,84 | 7,51E-03 | 0,59 | 2,11 |
| CREBBP | -1,84 | 5,39E-03 | 6,39 | 21,34 |
| LOXL3 | -1,84 | 2,89E-06 | 0,99 | 3,48 |
| MTFR2 | -1,84 | 3,93E-03 | 0,49 | 2,10 |
| OXSR1 | -1,84 | 8,39E-03 | 13,83 | 56,92 |
| TBL1X | -1,84 | 4,14E-04 | 5,23 | 20,53 |
| PRKCQ | -1,84 | 7,66E-03 | 0,66 | 2,46 |
| BUB1 | -1,84 | 3,85E-05 | 0,38 | 1,63 |
| TMEM2 | -1,84 | 7,65E-04 | 7,94 | 29,76 |
| ABCA6 | -1,84 | 4,58E-03 | 1,87 | 6,66 |
| PLEKHM1P1 | -1,84 | 4,85E-03 | 3,16 | 11,66 |
| FILIP1 | -1,83 | 3,10E-04 | 0,82 | 3,45 |
| TPP1 | -1,83 | 8,02E-04 | 28,39 | 134,19 |
| PHF19 | -1,83 | 5,19E-08 | 1,85 | 8,07 |
| NLRC5 | -1,83 | 3,61E-03 | 7,09 | 25,19 |
| FLVCR2 | -1,83 | 2,56E-04 | 1,13 | 4,83 |
| KRAS | -1,82 | 6,09E-03 | 6,17 | 27,71 |
| SNTB1 | -1,81 | 4,75E-05 | 0,92 | 3,61 |
| FERMT2 | -1,80 | 1,74E-04 | 5,59 | 24,07 |
| FYN | -1,80 | 2,86E-07 | 6,98 | 21,34 |
| TMEM119 | -1,80 | 5,74E-04 | 0,87 | 4,00 |
| STMN1 | -1,80 | 3,03E-04 | 19,15 | 75,32 |
| TMEM204 | -1,80 | 6,65E-03 | 2,57 | 10,99 |
| JAZF1 | -1,80 | 2,89E-03 | 4,31 | 19,72 |
| SH3RF3 | -1,79 | 9,62E-06 | 1,53 | 6,03 |
| TPM1 | -1,79 | 1,78E-04 | 30,97 | 133,00 |
| LMO2 | -1,79 | 6,19E-05 | 9,20 | 39,12 |
| SERPINH1 | -1,79 | 5,56E-05 | 22,97 | 89,19 |
| CCDC198 | -1,79 | 4,03E-04 | 1,93 | 7,07 |
| FSCN1 | -1,79 | 3,19E-04 | 2,70 | 11,25 |
| SGK1 | -1,79 | 1,05E-04 | 83,89 | 351,07 |
| CD44 | -1,78 | 6,97E-03 | 152,08 | 577,92 |
| TAP2 | -1,78 | 7,48E-03 | 12,46 | 38,43 |
| GIMAP8 | -1,78 | 1,40E-03 | 0,87 | 3,17 |
| EGFL7 | -1,78 | 2,26E-03 | 2,18 | 8,73 |
| IFFO1 | -1,78 | 6,91E-10 | 2,09 | 8,21 |
| RIPK2 | -1,77 | 2,03E-03 | 8,83 | 35,80 |
| SNX29 | -1,77 | 4,53E-06 | 2,14 | 9,08 |
| HIP1 | -1,77 | 4,42E-03 | 6,16 | 22,45 |
| AC009812.4 | -1,77 | 2,00E-03 | 0,54 | 2,32 |
| PRF1 | -1,77 | 1,12E-03 | 2,27 | 8,94 |
| PARVB | -1,76 | 5,08E-04 | 3,27 | 12,75 |
| PKD2 | -1,76 | 8,13E-07 | 5,18 | 15,69 |
| C20orf27 | -1,76 | 1,55E-05 | 4,11 | 17,47 |
| PIM1 | -1,76 | 8,94E-03 | 12,13 | 46,51 |
| SP4 | -1,76 | 8,68E-05 | 0,66 | 2,41 |
| MYO5A | -1,76 | 2,39E-03 | 3,02 | 9,93 |
| EIF4E3 | -1,76 | 1,40E-03 | 6,27 | 27,00 |
| PAQR3 | -1,75 | 6,19E-05 | 1,90 | 7,29 |
| LAMC1 | -1,75 | 3,47E-04 | 11,13 | 42,24 |
| GPRC5B | -1,74 | 6,39E-05 | 3,93 | 14,87 |
| SRGAP2B | -1,74 | 3,94E-03 | 2,14 | 7,31 |
| SAMD8 | -1,74 | 2,57E-03 | 4,04 | 15,43 |
| HIPK3 | -1,74 | 4,95E-03 | 11,18 | 39,58 |
| GUCY1B1 | -1,74 | 2,64E-03 | 1,98 | 8,34 |
| CHD2 | -1,73 | 7,57E-03 | 17,03 | 62,42 |
| RRM2 | -1,73 | 7,89E-05 | 1,06 | 4,89 |
| ADCY4 | -1,73 | 4,04E-03 | 2,15 | 7,17 |
| TFPI | -1,73 | 7,40E-03 | 4,97 | 17,15 |
| SCIN | -1,73 | 3,67E-05 | 2,51 | 8,26 |
| FAM84B | -1,73 | 2,63E-03 | 10,13 | 33,37 |
| EOMES | -1,72 | 7,63E-05 | 0,42 | 1,76 |
| STAT1 | -1,72 | 4,74E-03 | 20,18 | 72,29 |
| GINS4 | -1,72 | 1,53E-03 | 0,70 | 2,08 |
| PLPP1 | -1,72 | 1,82E-04 | 19,19 | 79,00 |
| RFFL | -1,72 | 7,20E-03 | 6,21 | 19,67 |
| GFOD1 | -1,72 | 5,41E-03 | 0,43 | 1,70 |
| TTPAL | -1,72 | 5,03E-04 | 3,02 | 10,94 |
| ESAM | -1,71 | 1,05E-03 | 1,46 | 5,64 |
| RASGRP3 | -1,70 | 1,73E-03 | 1,55 | 5,01 |
| ATXN1 | -1,70 | 8,45E-04 | 8,47 | 34,10 |
| MAGI2-AS3 | -1,70 | 1,16E-03 | 2,13 | 6,97 |
| FOXO1 | -1,70 | 1,09E-05 | 6,90 | 25,94 |
| PHC2 | -1,70 | 9,23E-03 | 30,56 | 109,57 |
| PHACTR2 | -1,70 | 2,16E-05 | 1,96 | 6,21 |
| HSPA12A | -1,69 | 1,39E-03 | 0,70 | 2,77 |
| RHOB | -1,69 | 2,85E-03 | 16,50 | 58,20 |
| KMT2C | -1,69 | 6,64E-03 | 8,05 | 25,42 |
| MTMR3 | -1,69 | 4,84E-03 | 9,37 | 36,05 |
| TMEM43 | -1,69 | 3,28E-04 | 14,74 | 50,77 |
| MAP1LC3B | -1,69 | 8,95E-03 | 31,63 | 117,65 |
| ARHGAP10 | -1,69 | 1,63E-03 | 1,85 | 7,14 |
| GTF2IP4 | -1,69 | 2,57E-03 | 7,73 | 26,47 |
| CEP170 | -1,68 | 2,91E-03 | 9,47 | 34,97 |
| KLHL5 | -1,68 | 5,93E-03 | 4,95 | 16,04 |
| PRICKLE2 | -1,68 | 4,88E-04 | 2,68 | 10,29 |
| LMO4 | -1,68 | 3,13E-03 | 16,10 | 66,03 |
| MAP7D3 | -1,68 | 1,47E-03 | 0,88 | 3,36 |
| TMEM200A | -1,68 | 3,41E-03 | 1,71 | 5,72 |
| SOD3 | -1,68 | 2,79E-03 | 2,16 | 8,59 |
| DST | -1,67 | 4,06E-03 | 19,34 | 82,09 |
| LINC01341 | -1,67 | 5,09E-06 | 2,89 | 8,19 |
| RASA3 | -1,67 | 5,95E-08 | 2,16 | 7,83 |
| ATP8B2 | -1,67 | 5,56E-05 | 1,68 | 5,74 |
| IMPA2 | -1,67 | 3,38E-03 | 10,84 | 40,31 |
| GDPD5 | -1,67 | 4,88E-04 | 1,53 | 5,93 |
| LRRC32 | -1,67 | 2,17E-03 | 1,36 | 5,12 |
| HTRA1 | -1,66 | 1,33E-05 | 25,43 | 94,81 |
| KMT2E | -1,66 | 5,11E-03 | 10,78 | 37,74 |
| ABL2 | -1,66 | 1,59E-03 | 1,67 | 6,12 |
| MAP3K20 | -1,66 | 3,65E-05 | 4,36 | 14,66 |
| RNF144A | -1,66 | 3,73E-05 | 1,84 | 6,79 |
| IGFBP6 | -1,66 | 1,01E-03 | 8,61 | 29,73 |
| DDR2 | -1,65 | 3,94E-03 | 2,48 | 11,69 |
| TWISTNB | -1,65 | 7,55E-04 | 7,06 | 22,65 |
| VPS13B | -1,65 | 1,64E-04 | 4,66 | 15,46 |
| BMP2K | -1,65 | 1,36E-03 | 5,14 | 21,65 |
| ADCY7 | -1,65 | 2,08E-03 | 2,91 | 10,17 |
| PSD | -1,65 | 8,39E-03 | 1,22 | 5,02 |
| SDC2 | -1,65 | 3,50E-04 | 19,60 | 56,75 |
| ZNF333 | -1,65 | 2,53E-03 | 3,50 | 13,58 |
| ABCG1 | -1,64 | 8,54E-03 | 7,25 | 24,72 |
| LRRC8C | -1,64 | 2,46E-05 | 0,76 | 2,72 |
| AQP1 | -1,64 | 3,18E-05 | 3,28 | 11,36 |
| GUSBP9 | -1,63 | 7,89E-03 | 1,05 | 4,28 |
| C3orf58 | -1,63 | 3,94E-04 | 2,57 | 7,31 |
| GAS2L3 | -1,63 | 1,29E-03 | 0,69 | 1,98 |
| ACVRL1 | -1,63 | 9,67E-03 | 2,92 | 10,38 |
| AL137003.2 | -1,62 | 5,84E-04 | 1,93 | 6,57 |
| sept.11 | -1,62 | 8,21E-06 | 8,81 | 31,72 |
| CORO1C | -1,62 | 4,06E-05 | 19,35 | 70,67 |
| YTHDF3 | -1,62 | 4,68E-03 | 11,73 | 40,08 |
| SLC36A1 | -1,62 | 8,00E-03 | 1,40 | 4,58 |
| DENND5B | -1,61 | 4,39E-04 | 0,44 | 1,60 |
| SEC23A | -1,61 | 8,53E-07 | 7,50 | 17,34 |
| PSD3 | -1,61 | 2,63E-03 | 1,65 | 5,41 |
| DNMBP | -1,60 | 2,52E-03 | 2,78 | 8,54 |
| PSAP | -1,60 | 9,50E-03 | 263,10 | 868,90 |
| RNF213 | -1,60 | 9,74E-03 | 28,53 | 81,54 |
| NR2C2 | -1,60 | 9,76E-03 | 2,69 | 8,05 |
| HOMER1 | -1,60 | 1,09E-03 | 1,93 | 5,92 |
| CCDC93 | -1,59 | 4,52E-03 | 9,36 | 37,92 |
| NFAT5 | -1,59 | 3,21E-03 | 7,03 | 20,18 |
| GBE1 | -1,59 | 1,72E-03 | 7,35 | 26,48 |
| PEAR1 | -1,59 | 1,51E-03 | 0,59 | 1,97 |
| LIMS2 | -1,59 | 8,26E-03 | 1,32 | 4,00 |
| MTMR10 | -1,59 | 6,47E-06 | 5,53 | 19,24 |
| GPAT4 | -1,59 | 8,46E-05 | 34,18 | 135,28 |
| CD1D | -1,59 | 8,46E-03 | 0,63 | 2,29 |
| NETO2 | -1,59 | 1,85E-05 | 1,00 | 3,12 |
| NBL1 | -1,59 | 4,97E-04 | 10,47 | 37,09 |
| SLC44A1 | -1,58 | 1,08E-03 | 9,21 | 41,91 |
| DTX3L | -1,58 | 4,98E-03 | 8,41 | 26,82 |
| ADARB1 | -1,58 | 3,78E-04 | 1,82 | 5,61 |
| CDK19 | -1,58 | 3,28E-03 | 2,96 | 9,82 |
| C12orf75 | -1,58 | 6,11E-03 | 22,37 | 84,52 |
| CAMKK1 | -1,58 | 2,06E-03 | 2,19 | 7,02 |
| MTMR11 | -1,58 | 2,40E-03 | 2,52 | 8,28 |
| PRR11 | -1,58 | 3,28E-03 | 0,50 | 2,00 |
| ZBTB10 | -1,58 | 8,91E-04 | 2,58 | 9,86 |
| CADM4 | -1,57 | 4,68E-03 | 4,54 | 12,33 |
| PLEKHO1 | -1,57 | 9,29E-03 | 11,17 | 36,37 |
| SNX18 | -1,57 | 9,99E-03 | 2,78 | 9,20 |
| VIM | -1,57 | 6,27E-05 | 242,19 | 836,67 |
| MAP3K5 | -1,57 | 1,30E-03 | 11,86 | 36,19 |
| KIF3C | -1,56 | 3,66E-03 | 0,53 | 1,66 |
| ATP6V1A | -1,56 | 1,24E-06 | 18,74 | 64,04 |
| KLF7 | -1,56 | 4,14E-03 | 3,73 | 11,01 |
| RTL5 | -1,56 | 5,40E-04 | 0,80 | 2,76 |
| ACOT9 | -1,56 | 6,80E-03 | 22,03 | 73,53 |
| PARP14 | -1,55 | 1,01E-03 | 13,23 | 43,94 |
| SERINC3 | -1,55 | 6,79E-03 | 30,89 | 92,32 |
| BUB1B | -1,55 | 9,21E-03 | 0,70 | 1,79 |
| KIF23 | -1,55 | 4,57E-03 | 0,64 | 1,87 |
| PHF20 | -1,55 | 1,67E-03 | 4,63 | 15,64 |
| DNAJC6 | -1,55 | 4,62E-03 | 0,50 | 1,52 |
| UBR4 | -1,55 | 3,81E-03 | 38,00 | 84,70 |
| SMPDL3A | -1,55 | 7,04E-05 | 8,17 | 28,46 |
| TRAM2 | -1,55 | 6,83E-03 | 1,54 | 5,23 |
| MIR100HG | -1,54 | 5,15E-03 | 5,23 | 17,45 |
| ITPR1 | -1,54 | 2,39E-03 | 3,34 | 9,10 |
| GNS | -1,54 | 3,14E-03 | 31,01 | 97,40 |
| CGAS | -1,54 | 6,97E-03 | 0,75 | 2,53 |
| FOXN3 | -1,53 | 4,21E-03 | 5,19 | 18,52 |
| SDC3 | -1,53 | 2,47E-07 | 4,78 | 16,14 |
| ABCC5 | -1,53 | 1,40E-03 | 7,90 | 24,35 |
| ITGA4 | -1,53 | 2,15E-03 | 3,18 | 10,07 |
| GPR173 | -1,53 | 4,63E-04 | 1,10 | 3,70 |
| TBC1D4 | -1,53 | 4,23E-03 | 2,46 | 9,00 |
| MOB1A | -1,53 | 4,77E-03 | 29,79 | 100,40 |
| EVC | -1,52 | 3,01E-03 | 1,84 | 6,14 |
| SYNE2 | -1,52 | 8,00E-03 | 29,10 | 73,96 |
| PTPRG | -1,52 | 7,15E-03 | 1,55 | 4,56 |
| ELK4 | -1,52 | 6,58E-07 | 3,59 | 12,06 |
| WNK1 | -1,52 | 3,27E-03 | 11,81 | 34,27 |
| FAM105A | -1,52 | 2,50E-03 | 1,54 | 6,23 |
| ANKH | -1,51 | 5,14E-06 | 3,04 | 8,41 |
| TRAK2 | -1,50 | 2,09E-05 | 5,05 | 17,99 |
| FANCA | -1,50 | 8,99E-03 | 2,30 | 5,35 |
| VHL | -1,50 | 4,43E-03 | 9,72 | 32,07 |
| DISC1 | -1,50 | 5,84E-03 | 0,88 | 2,81 |
| STAMBPL1 | -1,49 | 1,65E-03 | 1,58 | 5,55 |
| TCP11L1 | -1,49 | 2,53E-03 | 1,39 | 3,76 |
| SLC9A9 | -1,49 | 3,50E-04 | 0,79 | 2,71 |
| FHDC1 | -1,48 | 5,15E-03 | 1,25 | 3,86 |
| Z83843.1 | -1,48 | 3,63E-03 | 1,47 | 4,36 |
| EXOC8 | -1,48 | 6,05E-03 | 2,67 | 8,08 |
| ZNF708 | -1,48 | 9,73E-03 | 4,32 | 11,17 |
| HAAO | -1,48 | 6,45E-03 | 1,31 | 3,98 |
| STARD13 | -1,48 | 1,35E-04 | 1,83 | 6,56 |
| CCDC88A | -1,48 | 3,59E-03 | 2,75 | 8,88 |
| RCN3 | -1,48 | 6,96E-04 | 6,66 | 18,81 |
| SMG1 | -1,48 | 1,78E-03 | 8,73 | 25,96 |
| TOP2A | -1,47 | 1,35E-03 | 2,22 | 6,44 |
| OAS3 | -1,47 | 4,47E-03 | 2,41 | 7,89 |
| MYLIP | -1,47 | 1,71E-03 | 15,20 | 47,48 |
| ETS1 | -1,47 | 3,37E-03 | 5,45 | 16,42 |
| FXYD5 | -1,47 | 1,12E-04 | 16,09 | 46,65 |
| ITM2B | -1,47 | 5,10E-03 | 578,87 | 1467,49 |
| JAK1 | -1,47 | 9,48E-03 | 30,41 | 94,53 |
| ELMSAN1 | -1,46 | 1,14E-03 | 3,77 | 12,05 |
| ZBTB21 | -1,46 | 3,28E-03 | 2,33 | 7,41 |
| TGIF1 | -1,46 | 1,73E-03 | 29,95 | 76,81 |
| FAM219A | -1,46 | 1,33E-03 | 1,21 | 3,58 |
| ADAMTSL5 | -1,45 | 7,83E-03 | 0,78 | 2,13 |
| GNA12 | -1,45 | 5,23E-03 | 4,83 | 13,73 |
| ITSN1 | -1,45 | 1,51E-04 | 3,66 | 14,82 |
| SESN3 | -1,44 | 4,10E-04 | 3,31 | 10,12 |
| ZNF641 | -1,44 | 6,68E-03 | 7,96 | 25,45 |
| SYNE1 | -1,44 | 2,33E-03 | 7,39 | 22,59 |
| WWTR1 | -1,44 | 1,84E-03 | 6,49 | 18,57 |
| ATP2B4 | -1,42 | 2,35E-03 | 14,02 | 40,88 |
| VASH1 | -1,42 | 2,75E-03 | 3,29 | 9,45 |
| FBLIM1 | -1,42 | 5,82E-03 | 6,87 | 22,79 |
| FAM102B | -1,42 | 8,71E-03 | 2,19 | 6,38 |
| SMG1P3 | -1,42 | 7,01E-03 | 4,55 | 10,56 |
| PLEKHA1 | -1,42 | 6,67E-04 | 7,58 | 23,60 |
| CDC25B | -1,42 | 7,02E-03 | 3,71 | 11,73 |
| MAP3K12 | -1,42 | 5,26E-03 | 4,50 | 18,00 |
| METTL22 | -1,41 | 3,18E-03 | 5,88 | 17,24 |
| MBNL1 | -1,41 | 5,79E-03 | 14,55 | 44,47 |
| RAPGEF6 | -1,40 | 4,12E-03 | 2,54 | 8,06 |
| KCTD17 | -1,40 | 3,63E-03 | 1,78 | 5,03 |
| MERTK | -1,40 | 2,89E-03 | 1,93 | 4,88 |
| TMC8 | -1,40 | 1,28E-03 | 2,27 | 7,45 |
| EIF5A2 | -1,39 | 5,06E-03 | 0,69 | 2,33 |
| MTPN | -1,39 | 9,44E-03 | 24,35 | 66,36 |
| CLIP2 | -1,39 | 4,87E-05 | 1,17 | 3,57 |
| GIMAP6 | -1,39 | 1,54E-03 | 2,36 | 7,05 |
| FAM171A1 | -1,38 | 1,73E-03 | 2,89 | 8,96 |
| RBMS1 | -1,38 | 2,70E-03 | 16,31 | 46,87 |
| DOCK10 | -1,38 | 6,30E-03 | 2,96 | 9,95 |
| MX1 | -1,37 | 7,20E-03 | 13,14 | 39,04 |
| TIPARP | -1,37 | 5,09E-03 | 6,82 | 18,00 |
| ZNF100 | -1,37 | 6,15E-03 | 2,74 | 6,89 |
| CDKN1C | -1,37 | 7,15E-03 | 7,41 | 20,67 |
| SEMA6C | -1,36 | 5,35E-03 | 1,07 | 2,86 |
| SLC26A11 | -1,36 | 5,58E-03 | 2,90 | 9,00 |
| IL15RA | -1,36 | 7,23E-03 | 3,73 | 10,37 |
| PEAK1 | -1,36 | 7,95E-05 | 1,93 | 5,91 |
| GPRASP1 | -1,36 | 1,54E-03 | 1,15 | 3,48 |
| SLC1A2 | -1,36 | 1,84E-03 | 0,55 | 1,63 |
| SHROOM4 | -1,36 | 7,09E-03 | 1,01 | 2,06 |
| RNF111 | -1,35 | 9,75E-03 | 5,86 | 16,50 |
| TXK | -1,35 | 3,00E-03 | 1,24 | 3,00 |
| ITPR2 | -1,35 | 8,31E-03 | 2,51 | 6,62 |
| SNX8 | -1,35 | 4,39E-03 | 5,99 | 17,31 |
| MOB3B | -1,35 | 5,48E-06 | 1,63 | 4,71 |
| PLEKHG4 | -1,34 | 4,08E-03 | 0,82 | 2,18 |
| MED13 | -1,34 | 2,93E-03 | 5,66 | 16,45 |
| HELZ | -1,34 | 3,12E-05 | 2,97 | 8,70 |
| SPN | -1,33 | 4,19E-03 | 1,37 | 4,31 |
| HCG18 | -1,33 | 6,84E-03 | 7,86 | 21,05 |
| DPY19L3 | -1,33 | 6,54E-04 | 3,17 | 9,15 |
| KDM1B | -1,32 | 6,32E-03 | 3,10 | 7,73 |
| MDFIC | -1,32 | 5,82E-04 | 3,76 | 6,55 |
| TGFBR1 | -1,32 | 7,87E-03 | 6,39 | 16,64 |
| TMOD1 | -1,32 | 6,11E-03 | 3,11 | 9,08 |
| DBN1 | -1,32 | 7,98E-04 | 6,10 | 18,38 |
| MRAS | -1,32 | 9,13E-04 | 2,21 | 6,54 |
| FAM149A | -1,32 | 4,30E-03 | 5,65 | 16,51 |
| INPP5A | -1,32 | 4,02E-03 | 4,85 | 14,08 |
| INPP4A | -1,31 | 3,36E-03 | 2,76 | 7,33 |
| GLS | -1,31 | 1,14E-03 | 11,97 | 35,58 |
| CPM | -1,31 | 1,78E-04 | 4,66 | 18,18 |
| GPATCH2L | -1,31 | 1,84E-03 | 4,21 | 13,70 |
| ZNF281 | -1,31 | 5,23E-03 | 3,19 | 8,50 |
| GOLIM4 | -1,31 | 1,57E-03 | 2,49 | 6,66 |
| HSD3B7 | -1,30 | 2,70E-03 | 4,06 | 11,58 |
| RNF11 | -1,30 | 4,85E-03 | 25,84 | 49,21 |
| NLN | -1,30 | 7,92E-05 | 2,83 | 10,08 |
| KATNAL1 | -1,29 | 1,14E-03 | 1,06 | 3,15 |
| RHOBTB1 | -1,29 | 1,09E-03 | 3,52 | 9,22 |
| TAB3 | -1,29 | 6,32E-03 | 4,45 | 12,10 |
| MAPRE2 | -1,28 | 4,60E-04 | 2,96 | 7,79 |
| AGPAT4 | -1,28 | 2,54E-04 | 3,38 | 10,61 |
| KLF11 | -1,28 | 2,78E-05 | 1,93 | 5,66 |
| PIKFYVE | -1,28 | 6,31E-04 | 3,21 | 8,53 |
| TNS1 | -1,28 | 8,39E-03 | 4,72 | 12,64 |
| MAOB | -1,27 | 1,77E-03 | 8,06 | 24,04 |
| ADNP2 | -1,27 | 7,18E-03 | 2,42 | 6,66 |
| PPARG | -1,27 | 9,44E-03 | 1,05 | 2,82 |
| RAB32 | -1,26 | 3,26E-03 | 8,18 | 23,52 |
| SDK1 | -1,25 | 6,91E-03 | 1,43 | 4,30 |
| STARD4 | -1,25 | 8,66E-03 | 4,05 | 8,94 |
| GNPDA1 | -1,25 | 4,47E-03 | 11,32 | 33,56 |
| AC009948.1 | -1,24 | 2,24E-03 | 2,39 | 7,12 |
| IREB2 | -1,24 | 3,29E-04 | 5,63 | 15,37 |
| GUSBP11 | -1,23 | 1,79E-03 | 14,70 | 29,67 |
| ZNF532 | -1,23 | 4,10E-03 | 6,81 | 17,90 |
| RAP2A | -1,23 | 1,98E-04 | 2,69 | 6,33 |
| GPRIN3 | -1,23 | 9,44E-03 | 1,08 | 2,79 |
| UVRAG | -1,23 | 4,99E-03 | 2,97 | 8,28 |
| CX3CR1 | -1,22 | 4,23E-03 | 2,26 | 6,06 |
| FNBP1 | -1,22 | 8,94E-03 | 13,80 | 35,99 |
| GINS3 | -1,22 | 6,24E-03 | 2,07 | 5,96 |
| MSL2 | -1,22 | 5,67E-03 | 5,60 | 14,84 |
| RIPOR1 | -1,22 | 7,79E-04 | 6,44 | 16,89 |
| LIN37 | -1,21 | 9,74E-03 | 4,07 | 9,50 |
| NR2F2 | -1,21 | 6,80E-03 | 10,73 | 29,20 |
| USF3 | -1,21 | 5,71E-03 | 4,65 | 8,88 |
| GDI1 | -1,21 | 8,22E-03 | 26,74 | 70,66 |
| SSH1 | -1,20 | 1,95E-03 | 2,97 | 7,94 |
| RASA4 | -1,20 | 9,45E-04 | 2,66 | 7,88 |
| EIF3A | -1,20 | 5,96E-03 | 19,34 | 47,37 |
| GRN | -1,19 | 6,71E-03 | 131,96 | 268,88 |
| ZFYVE26 | -1,19 | 9,67E-03 | 3,00 | 8,06 |
| PPM1D | -1,19 | 3,97E-03 | 2,68 | 6,83 |
| ARHGAP11A | -1,19 | 3,53E-03 | 0,67 | 1,62 |
| CD247 | -1,18 | 7,40E-03 | 3,40 | 9,66 |
| KIDINS220 | -1,18 | 2,48E-03 | 9,39 | 24,20 |
| YWHAG | -1,18 | 6,46E-03 | 13,60 | 33,20 |
| TFIP11 | -1,18 | 4,02E-03 | 12,59 | 29,54 |
| EPG5 | -1,16 | 6,67E-04 | 4,51 | 10,53 |
| ZEB1-AS1 | -1,16 | 5,25E-03 | 1,74 | 5,03 |
| BLOC1S6 | -1,16 | 8,29E-03 | 10,41 | 26,38 |
| RIC1 | -1,15 | 3,93E-03 | 3,54 | 8,78 |
| MALT1 | -1,15 | 4,72E-03 | 6,06 | 13,33 |
| CHML | -1,15 | 2,86E-03 | 1,13 | 3,00 |
| GXYLT1 | -1,15 | 3,78E-03 | 1,61 | 4,41 |
| SESTD1 | -1,15 | 1,59E-04 | 15,55 | 42,73 |
| NUSAP1 | -1,15 | 2,84E-03 | 2,61 | 5,37 |
| ARMCX1 | -1,15 | 2,66E-03 | 2,95 | 8,10 |
| LTBP4 | -1,14 | 9,70E-03 | 14,41 | 35,14 |
| SERINC5 | -1,14 | 2,61E-03 | 5,39 | 14,45 |
| NXPE3 | -1,14 | 5,82E-03 | 1,05 | 3,26 |
| RGL1 | -1,14 | 9,93E-05 | 5,65 | 14,66 |
| SLC25A13 | -1,13 | 1,33E-03 | 8,48 | 26,75 |
| SAP30BP | -1,13 | 3,30E-03 | 37,69 | 79,43 |
| SNX9 | -1,13 | 9,02E-03 | 10,37 | 25,29 |
| ATP6V0A1 | -1,13 | 9,25E-03 | 9,28 | 21,20 |
| SOAT1 | -1,13 | 6,27E-03 | 12,47 | 33,84 |
| KCTD10 | -1,13 | 9,88E-04 | 6,15 | 15,27 |
| ARNT | -1,12 | 3,70E-05 | 7,19 | 18,43 |
| ARIH1 | -1,12 | 8,71E-03 | 13,01 | 35,41 |
| FEM1C | -1,12 | 7,45E-03 | 3,26 | 8,20 |
| PHTF2 | -1,12 | 1,73E-03 | 2,21 | 5,67 |
| TMED5 | -1,12 | 9,29E-03 | 13,32 | 36,42 |
| TTL | -1,12 | 2,55E-04 | 3,93 | 10,63 |
| SFXN3 | -1,12 | 4,43E-04 | 9,11 | 24,65 |
| SNTB2 | -1,12 | 1,40E-03 | 1,66 | 4,01 |
| ZNF516 | -1,11 | 2,28E-03 | 12,84 | 29,92 |
| RRN3P1 | -1,11 | 5,91E-03 | 4,08 | 10,56 |
| SACS | -1,10 | 5,27E-03 | 0,89 | 2,71 |
| GAL3ST4 | -1,10 | 4,43E-03 | 1,00 | 2,47 |
| NCOA3 | -1,10 | 1,35E-03 | 5,38 | 12,86 |
| TMOD3 | -1,10 | 8,92E-03 | 9,71 | 20,65 |
| MTMR9 | -1,09 | 1,51E-03 | 1,72 | 3,58 |
| C5orf51 | -1,09 | 6,83E-03 | 4,79 | 12,13 |
| ACER3 | -1,09 | 3,67E-05 | 4,58 | 9,50 |
| CTSA | -1,08 | 2,00E-03 | 40,34 | 98,72 |
| MOB1B | -1,08 | 3,41E-03 | 3,09 | 7,44 |
| CDK17 | -1,08 | 5,82E-03 | 5,96 | 11,09 |
| HP1BP3 | -1,08 | 8,04E-03 | 34,15 | 78,04 |
| SPRED1 | -1,07 | 1,44E-03 | 2,14 | 4,94 |
| BICRAL | -1,07 | 7,00E-03 | 2,77 | 6,35 |
| CCDC18 | -1,07 | 8,78E-03 | 1,39 | 3,15 |
| ZC3HAV1 | -1,07 | 8,08E-05 | 9,19 | 24,81 |
| sept.06 | -1,06 | 9,92E-04 | 5,90 | 14,53 |
| AFF4 | -1,06 | 9,40E-03 | 10,86 | 24,44 |
| GALNT11 | -1,06 | 4,85E-03 | 13,38 | 36,97 |
| C6orf62 | -1,06 | 7,45E-03 | 48,67 | 87,19 |
| WARS | -1,06 | 9,93E-03 | 40,58 | 108,02 |
| TAOK1 | -1,05 | 5,84E-03 | 5,90 | 14,18 |
| PTPN11 | -1,05 | 2,25E-05 | 8,78 | 21,40 |
| ARL15 | -1,05 | 4,85E-03 | 4,82 | 10,04 |
| TBC1D2B | -1,05 | 1,29E-04 | 7,39 | 18,78 |
| ATP11C | -1,04 | 2,37E-03 | 4,24 | 10,20 |
| C5orf24 | -1,04 | 2,33E-04 | 6,92 | 16,33 |
| KLHL42 | -1,04 | 9,74E-03 | 2,25 | 6,07 |
| ITGB1 | -1,04 | 5,47E-04 | 70,78 | 170,82 |
| SLC35F5 | -1,03 | 1,01E-03 | 17,23 | 36,61 |
| SEL1L | -1,03 | 9,76E-03 | 11,49 | 26,60 |
| DPY19L1 | -1,02 | 1,20E-03 | 3,26 | 6,86 |
| CBLL1 | -1,02 | 2,65E-03 | 3,86 | 7,86 |
| LMBR1L | -1,02 | 7,39E-03 | 15,53 | 38,12 |
| DENND6A | -1,01 | 9,90E-04 | 4,31 | 7,96 |
| CAMK1 | -1,01 | 4,62E-03 | 3,71 | 8,76 |
| DCTN4 | -1,01 | 7,15E-03 | 11,88 | 28,51 |
| EIF4G3 | -1,00 | 3,54E-03 | 9,54 | 20,48 |
| PALLD | -1,00 | 9,49E-03 | 18,38 | 48,14 |
| PPP2R1B | 1,00 | 9,66E-03 | 23,75 | 11,95 |
| ARRDC1 | 1,00 | 3,60E-03 | 40,66 | 22,85 |
| GSTK1 | 1,02 | 1,03E-05 | 213,59 | 123,18 |
| BAG1 | 1,02 | 1,55E-05 | 77,16 | 44,53 |
| C9orf16 | 1,02 | 2,64E-03 | 49,54 | 27,97 |
| SLC2A10 | 1,02 | 2,55E-03 | 10,75 | 5,91 |
| PGPEP1 | 1,02 | 7,01E-06 | 32,90 | 13,93 |
| MRPL23 | 1,02 | 4,21E-03 | 50,31 | 31,50 |
| SLC18B1 | 1,02 | 9,99E-03 | 9,93 | 5,94 |
| TIMMDC1 | 1,03 | 7,58E-03 | 91,71 | 53,65 |
| FAM83H | 1,03 | 8,60E-03 | 7,72 | 4,11 |
| ATP5F1D | 1,03 | 7,45E-03 | 56,16 | 34,38 |
| BOLA1 | 1,04 | 7,49E-03 | 14,35 | 8,71 |
| LHPP | 1,04 | 1,97E-03 | 10,20 | 5,52 |
| ARL1 | 1,04 | 3,03E-03 | 99,45 | 44,26 |
| LURAP1L | 1,04 | 9,74E-03 | 9,15 | 4,99 |
| H2AFJ | 1,05 | 4,85E-03 | 36,07 | 19,57 |
| MRPL57 | 1,05 | 6,81E-03 | 12,61 | 7,57 |
| PSMG3 | 1,05 | 5,07E-03 | 41,21 | 24,22 |
| FAM174B | 1,05 | 8,34E-03 | 24,67 | 12,44 |
| CCDC106 | 1,06 | 2,50E-03 | 6,19 | 4,11 |
| AK2 | 1,06 | 8,83E-05 | 118,00 | 64,01 |
| SLC50A1 | 1,06 | 1,80E-04 | 51,42 | 32,69 |
| ANK3 | 1,06 | 1,81E-03 | 20,08 | 10,52 |
| WBP1 | 1,06 | 5,45E-04 | 71,50 | 40,21 |
| SRPRB | 1,06 | 4,19E-04 | 40,91 | 27,64 |
| ALDH4A1 | 1,07 | 4,27E-03 | 5,91 | 3,33 |
| ILVBL | 1,07 | 3,78E-03 | 24,01 | 14,14 |
| FDPS | 1,07 | 2,68E-03 | 158,07 | 90,69 |
| HDAC11 | 1,07 | 1,42E-03 | 9,31 | 5,75 |
| TFPT | 1,07 | 1,50E-03 | 19,84 | 9,03 |
| C6orf136 | 1,08 | 4,61E-03 | 15,57 | 7,33 |
| CHRNB1 | 1,08 | 6,96E-03 | 5,91 | 3,86 |
| PMM1 | 1,08 | 1,83E-03 | 34,87 | 19,80 |
| PFN2 | 1,08 | 1,48E-03 | 32,41 | 19,46 |
| GIPC1 | 1,08 | 4,52E-03 | 54,19 | 30,75 |
| KIF2A | 1,09 | 1,34E-03 | 21,78 | 13,11 |
| PTGR2 | 1,09 | 5,47E-03 | 6,88 | 4,11 |
| MTURN | 1,09 | 2,24E-03 | 19,94 | 11,19 |
| SIX4 | 1,09 | 8,68E-07 | 3,96 | 2,23 |
| HOXB7 | 1,09 | 1,61E-04 | 27,71 | 15,57 |
| PPP1R16A | 1,09 | 4,18E-05 | 22,44 | 11,03 |
| TREX1 | 1,09 | 1,22E-03 | 18,32 | 9,39 |
| ENPP4 | 1,09 | 9,24E-03 | 10,38 | 6,06 |
| DHRS7B | 1,09 | 9,62E-03 | 48,70 | 25,86 |
| TNFRSF19 | 1,10 | 1,47E-03 | 6,13 | 3,56 |
| ARVCF | 1,10 | 9,51E-03 | 7,75 | 4,11 |
| KIAA0895 | 1,10 | 4,63E-03 | 4,72 | 2,53 |
| PEX11G | 1,10 | 8,13E-03 | 4,75 | 2,67 |
| WDR34 | 1,11 | 2,50E-03 | 36,47 | 21,84 |
| CTNNBIP1 | 1,11 | 3,63E-03 | 18,97 | 12,87 |
| CUTA | 1,11 | 1,63E-03 | 130,62 | 72,66 |
| CDS1 | 1,11 | 6,17E-03 | 12,17 | 7,15 |
| FAM173A | 1,11 | 4,58E-03 | 13,55 | 7,44 |
| RPARP-AS1 | 1,11 | 1,15E-03 | 13,82 | 7,30 |
| ISCA2 | 1,12 | 1,15E-03 | 26,58 | 13,45 |
| SCRN2 | 1,12 | 7,90E-04 | 14,82 | 8,78 |
| EMC9 | 1,12 | 2,56E-03 | 13,22 | 7,56 |
| PRSS16 | 1,12 | 2,76E-04 | 14,59 | 8,37 |
| ACSS1 | 1,12 | 2,46E-03 | 10,80 | 5,90 |
| PARP16 | 1,12 | 7,57E-03 | 8,14 | 4,39 |
| GLCE | 1,12 | 8,87E-04 | 9,73 | 5,46 |
| OCIAD2 | 1,12 | 8,83E-03 | 52,06 | 28,75 |
| ALDH3A2 | 1,13 | 3,53E-03 | 34,26 | 20,46 |
| RBBP9 | 1,13 | 3,77E-04 | 13,63 | 7,92 |
| ALDH18A1 | 1,13 | 5,13E-03 | 28,86 | 16,61 |
| YIPF2 | 1,13 | 3,62E-04 | 26,85 | 15,99 |
| GON7 | 1,13 | 2,66E-03 | 26,81 | 15,49 |
| CCDC30 | 1,13 | 6,56E-04 | 7,46 | 3,24 |
| DNAJA4 | 1,14 | 1,07E-03 | 35,16 | 19,23 |
| PIGH | 1,14 | 3,07E-04 | 31,44 | 16,99 |
| MPHOSPH6 | 1,14 | 4,49E-04 | 50,32 | 24,23 |
| TMCO4 | 1,14 | 3,61E-03 | 15,95 | 8,48 |
| FAM162A | 1,14 | 7,83E-03 | 50,07 | 29,65 |
| ZNF219 | 1,15 | 1,46E-04 | 5,76 | 3,21 |
| HOXB2 | 1,15 | 3,96E-04 | 15,13 | 7,64 |
| RPL36AL | 1,16 | 8,39E-03 | 412,21 | 233,24 |
| ATF7IP2 | 1,16 | 6,88E-04 | 17,74 | 6,38 |
| CUEDC1 | 1,16 | 3,50E-06 | 25,67 | 17,50 |
| SLC39A11 | 1,17 | 6,71E-04 | 19,16 | 11,14 |
| RAP1GAP | 1,17 | 2,03E-03 | 6,92 | 3,55 |
| ZNF334 | 1,17 | 5,80E-03 | 9,89 | 5,36 |
| KDF1 | 1,17 | 8,52E-03 | 6,22 | 3,31 |
| ANKEF1 | 1,18 | 6,29E-04 | 2,59 | 1,37 |
| GOLM1 | 1,18 | 4,68E-04 | 75,86 | 41,57 |
| GSTO2 | 1,18 | 4,45E-04 | 25,06 | 11,05 |
| POLR2H | 1,18 | 8,00E-03 | 104,96 | 58,81 |
| ARFIP2 | 1,18 | 6,56E-04 | 47,56 | 25,89 |
| TST | 1,18 | 4,50E-04 | 56,15 | 29,72 |
| NEIL1 | 1,18 | 5,80E-03 | 39,10 | 18,71 |
| NDUFA6-AS1 | 1,18 | 3,73E-03 | 3,16 | 1,78 |
| RITA1 | 1,18 | 1,48E-03 | 8,74 | 4,81 |
| EVA1A | 1,19 | 8,16E-03 | 8,72 | 4,23 |
| TMPRSS4 | 1,19 | 3,59E-03 | 142,90 | 72,89 |
| SHC2 | 1,19 | 1,67E-03 | 9,78 | 5,42 |
| SPINT2 | 1,19 | 1,05E-03 | 372,06 | 193,91 |
| PRRG2 | 1,19 | 1,40E-03 | 7,35 | 3,59 |
| ZIK1 | 1,19 | 5,34E-04 | 2,60 | 1,40 |
| TECR | 1,20 | 3,12E-03 | 64,16 | 29,03 |
| ATP5IF1 | 1,20 | 9,20E-04 | 269,70 | 135,55 |
| SMIM20 | 1,20 | 5,78E-03 | 31,69 | 17,79 |
| DDO | 1,20 | 1,04E-03 | 3,40 | 1,88 |
| TXNDC17 | 1,20 | 6,84E-03 | 158,44 | 82,80 |
| SLC25A12 | 1,21 | 1,61E-03 | 15,02 | 8,41 |
| LLGL2 | 1,21 | 5,93E-03 | 89,03 | 38,51 |
| ATP8B1 | 1,21 | 9,26E-04 | 12,71 | 5,88 |
| TMEM59 | 1,21 | 9,98E-03 | 472,35 | 242,27 |
| SLC46A1 | 1,21 | 4,33E-03 | 4,89 | 2,74 |
| FBXO36 | 1,22 | 2,86E-03 | 3,28 | 1,77 |
| GCAT | 1,22 | 7,57E-03 | 9,98 | 5,64 |
| DRC3 | 1,22 | 6,80E-03 | 21,21 | 11,56 |
| HSBP1L1 | 1,22 | 3,84E-04 | 25,96 | 13,85 |
| MAGEF1 | 1,22 | 2,49E-04 | 25,71 | 13,23 |
| BAIAP2L1 | 1,22 | 7,57E-03 | 13,70 | 6,67 |
| SORBS2 | 1,22 | 3,30E-03 | 13,27 | 6,28 |
| CISD1 | 1,23 | 6,30E-03 | 50,89 | 27,09 |
| AC027644.3 | 1,23 | 2,70E-04 | 15,18 | 7,64 |
| NPDC1 | 1,23 | 9,52E-03 | 60,18 | 32,75 |
| LRRC23 | 1,23 | 5,65E-03 | 23,02 | 12,67 |
| OCEL1 | 1,23 | 8,54E-03 | 26,54 | 11,25 |
| HADH | 1,23 | 7,65E-04 | 28,46 | 14,49 |
| KCTD6 | 1,24 | 1,36E-03 | 13,92 | 7,45 |
| C6orf226 | 1,24 | 4,58E-03 | 11,70 | 6,23 |
| NMNAT3 | 1,24 | 6,26E-03 | 2,03 | 1,17 |
| STAP2 | 1,24 | 1,04E-03 | 18,34 | 8,45 |
| HSPE1 | 1,24 | 4,69E-03 | 147,90 | 83,32 |
| MICALL2 | 1,24 | 4,42E-03 | 31,14 | 13,17 |
| MRPL14 | 1,24 | 3,07E-03 | 46,05 | 24,75 |
| C17orf97 | 1,24 | 2,44E-03 | 6,22 | 3,38 |
| ROMO1 | 1,24 | 5,63E-03 | 167,81 | 91,74 |
| SDF2L1 | 1,25 | 2,18E-04 | 39,23 | 19,86 |
| ATG101 | 1,25 | 6,53E-04 | 33,17 | 17,79 |
| FIS1 | 1,25 | 9,76E-03 | 163,74 | 89,39 |
| LRRC61 | 1,25 | 1,02E-05 | 9,39 | 5,01 |
| TRPM4 | 1,26 | 2,39E-03 | 33,17 | 14,22 |
| HOXB3 | 1,26 | 1,01E-04 | 38,95 | 18,55 |
| LPAR3 | 1,26 | 3,49E-04 | 9,11 | 4,64 |
| FMC1 | 1,26 | 8,53E-03 | 28,85 | 16,35 |
| CYB561 | 1,26 | 3,21E-04 | 57,85 | 28,69 |
| VANGL2 | 1,26 | 9,03E-03 | 6,68 | 3,17 |
| MYL6B | 1,26 | 8,80E-03 | 104,53 | 35,86 |
| TMEM30B | 1,26 | 2,83E-03 | 13,46 | 6,76 |
| SSR4 | 1,26 | 2,29E-03 | 582,57 | 316,87 |
| CENPS | 1,27 | 2,01E-03 | 13,32 | 7,23 |
| PCDH7 | 1,27 | 1,78E-03 | 10,20 | 4,80 |
| AK9 | 1,27 | 3,91E-03 | 6,98 | 4,44 |
| NDUFAF8 | 1,27 | 4,77E-03 | 53,66 | 31,68 |
| PKP2 | 1,28 | 5,57E-03 | 17,02 | 8,08 |
| BDH1 | 1,28 | 1,78E-03 | 21,46 | 11,18 |
| CHCHD6 | 1,28 | 4,31E-03 | 8,10 | 4,20 |
| CENPX | 1,28 | 2,51E-03 | 39,81 | 20,53 |
| ADHFE1 | 1,28 | 3,14E-04 | 12,60 | 4,32 |
| ALKBH7 | 1,29 | 9,55E-04 | 52,49 | 27,03 |
| TMEM56 | 1,29 | 3,58E-03 | 1,61 | 0,80 |
| CFAP69 | 1,29 | 9,64E-03 | 11,23 | 5,39 |
| HENMT1 | 1,29 | 1,30E-03 | 10,40 | 5,30 |
| C11orf80 | 1,29 | 5,92E-06 | 18,02 | 9,87 |
| FAAH | 1,30 | 1,09E-05 | 14,67 | 6,56 |
| MRPL17 | 1,30 | 6,17E-03 | 40,24 | 20,14 |
| AC021097.1 | 1,30 | 7,16E-03 | 5,63 | 2,77 |
| ZNF503 | 1,30 | 1,32E-04 | 5,95 | 2,82 |
| NFIA | 1,31 | 2,55E-03 | 5,18 | 2,57 |
| SRP14-AS1 | 1,31 | 3,59E-03 | 6,33 | 3,07 |
| SAMD12 | 1,31 | 3,87E-05 | 4,75 | 1,92 |
| ST6GALNAC4 | 1,32 | 2,16E-04 | 13,54 | 6,86 |
| FN3K | 1,32 | 9,04E-03 | 7,38 | 3,60 |
| TLE2 | 1,32 | 6,30E-04 | 16,86 | 7,58 |
| CD9 | 1,32 | 6,35E-03 | 487,97 | 244,11 |
| CLDN7 | 1,33 | 6,15E-03 | 97,63 | 47,66 |
| SULT1A3 | 1,33 | 1,84E-03 | 28,85 | 14,02 |
| PECR | 1,33 | 9,97E-03 | 8,93 | 5,53 |
| TMEM53 | 1,33 | 3,75E-04 | 9,71 | 4,21 |
| TOM1L1 | 1,33 | 7,36E-03 | 41,77 | 21,53 |
| KLHL14 | 1,33 | 1,31E-03 | 3,21 | 1,52 |
| LRRC45 | 1,33 | 3,08E-04 | 6,47 | 3,49 |
| TPBG | 1,34 | 7,88E-04 | 26,22 | 11,96 |
| C9orf152 | 1,34 | 1,90E-05 | 12,94 | 6,09 |
| NCK1-AS1 | 1,34 | 6,80E-03 | 4,68 | 2,18 |
| BEND5 | 1,34 | 3,52E-03 | 3,90 | 1,51 |
| SYNPO | 1,34 | 6,64E-04 | 6,54 | 3,02 |
| INAVA | 1,35 | 3,26E-04 | 15,87 | 6,29 |
| NME3 | 1,35 | 7,18E-04 | 47,66 | 22,96 |
| CMTM8 | 1,35 | 3,39E-04 | 7,37 | 3,39 |
| SMDT1 | 1,35 | 3,22E-04 | 79,51 | 38,50 |
| IL20RA | 1,35 | 2,41E-03 | 55,61 | 24,21 |
| SYT17 | 1,35 | 3,18E-05 | 3,66 | 1,64 |
| CCDC78 | 1,35 | 4,63E-03 | 17,87 | 8,88 |
| HLA-DMB | 1,35 | 4,82E-03 | 187,88 | 75,48 |
| CXXC5 | 1,35 | 6,54E-04 | 28,74 | 16,57 |
| ARHGEF16 | 1,35 | 5,17E-03 | 10,23 | 4,55 |
| PLEKHA8P1 | 1,35 | 9,21E-03 | 2,77 | 1,96 |
| NDUFA2 | 1,35 | 7,24E-03 | 113,74 | 58,63 |
| DLG3 | 1,35 | 2,43E-04 | 11,31 | 6,62 |
| LRTOMT | 1,36 | 1,53E-03 | 22,19 | 11,14 |
| AMY2B | 1,36 | 2,37E-04 | 64,56 | 30,51 |
| MFSD6L | 1,36 | 1,67E-04 | 2,89 | 1,35 |
| MAATS1 | 1,36 | 2,02E-03 | 11,71 | 5,53 |
| AL513477.1 | 1,36 | 9,90E-03 | 3,88 | 1,75 |
| SLC9A3R1 | 1,36 | 2,25E-04 | 56,88 | 25,54 |
| HOXB6 | 1,36 | 1,98E-05 | 47,16 | 21,43 |
| ZNF702P | 1,36 | 2,41E-03 | 7,86 | 4,57 |
| KRTCAP3 | 1,37 | 3,42E-06 | 36,53 | 15,28 |
| SYBU | 1,37 | 8,16E-03 | 7,70 | 3,26 |
| MAPK15 | 1,37 | 2,55E-03 | 10,65 | 5,07 |
| ATP1B1 | 1,38 | 2,81E-03 | 49,95 | 23,48 |
| TRAPPC6A | 1,38 | 5,63E-03 | 26,33 | 13,03 |
| CCDC103 | 1,38 | 5,93E-03 | 3,44 | 1,63 |
| FAIM | 1,38 | 6,11E-03 | 52,45 | 23,32 |
| ID1 | 1,38 | 6,24E-03 | 162,60 | 74,30 |
| RARRES3 | 1,38 | 1,49E-05 | 122,97 | 59,08 |
| NR2F6 | 1,38 | 4,30E-04 | 24,47 | 11,60 |
| SYNE4 | 1,39 | 7,21E-05 | 8,16 | 3,71 |
| ARHGAP44 | 1,39 | 2,19E-03 | 2,09 | 0,93 |
| FOXJ1 | 1,39 | 1,93E-03 | 19,60 | 9,43 |
| ST14 | 1,40 | 2,01E-04 | 39,45 | 17,77 |
| PPDPF | 1,40 | 3,80E-05 | 139,65 | 63,78 |
| PHKA1 | 1,40 | 1,23E-04 | 2,11 | 0,92 |
| KIAA1841 | 1,40 | 2,57E-05 | 25,37 | 7,78 |
| FAAH2 | 1,41 | 2,53E-03 | 22,66 | 10,16 |
| HSD17B8 | 1,41 | 4,44E-04 | 16,97 | 7,85 |
| GALNT3 | 1,41 | 2,82E-04 | 53,67 | 25,45 |
| SPINT1 | 1,41 | 7,76E-03 | 102,42 | 45,96 |
| PPIL6 | 1,41 | 9,67E-03 | 7,00 | 4,57 |
| TMED3 | 1,41 | 2,77E-04 | 99,35 | 44,86 |
| TTC39A | 1,41 | 2,61E-04 | 18,17 | 6,68 |
| ARV1 | 1,41 | 3,92E-03 | 17,65 | 8,72 |
| SKAP1 | 1,41 | 7,24E-03 | 22,61 | 9,40 |
| AC025181.2 | 1,41 | 9,20E-03 | 3,19 | 1,41 |
| SPATA18 | 1,41 | 1,77E-05 | 19,04 | 8,77 |
| SRGAP3 | 1,41 | 5,33E-03 | 9,15 | 3,86 |
| TEKT2 | 1,41 | 7,20E-03 | 9,71 | 4,72 |
| DCXR | 1,41 | 4,70E-03 | 62,30 | 29,67 |
| SPATA6L | 1,42 | 3,84E-05 | 4,36 | 1,93 |
| ITIH4 | 1,42 | 3,75E-06 | 14,57 | 5,43 |
| SCP2 | 1,42 | 5,97E-03 | 179,60 | 76,88 |
| VAMP8 | 1,42 | 6,66E-06 | 236,78 | 110,83 |
| DNAJB13 | 1,42 | 1,41E-03 | 27,71 | 14,28 |
| KLHDC9 | 1,42 | 2,76E-03 | 15,04 | 7,05 |
| CCDC96 | 1,42 | 7,61E-03 | 3,09 | 1,47 |
| KRT18 | 1,42 | 9,71E-04 | 346,56 | 148,74 |
| NEK11 | 1,43 | 8,00E-03 | 13,89 | 6,74 |
| SH2D4A | 1,43 | 1,15E-09 | 18,76 | 9,01 |
| SLC25A10 | 1,43 | 7,20E-03 | 10,25 | 4,81 |
| SLC25A4 | 1,43 | 2,01E-04 | 11,29 | 5,10 |
| KIAA1522 | 1,43 | 2,68E-06 | 29,31 | 13,43 |
| CFAP44 | 1,43 | 2,66E-04 | 25,30 | 11,53 |
| GYG2 | 1,43 | 2,88E-03 | 4,51 | 1,89 |
| RARB | 1,43 | 3,12E-03 | 5,73 | 2,58 |
| LINC00908 | 1,43 | 6,06E-04 | 3,01 | 1,43 |
| SERTAD4 | 1,43 | 3,18E-05 | 3,75 | 1,52 |
| SPAG8 | 1,43 | 8,38E-03 | 10,70 | 4,32 |
| NDUFA3 | 1,43 | 6,30E-05 | 160,07 | 64,93 |
| SGSM3 | 1,44 | 9,91E-15 | 61,66 | 24,36 |
| GAMT | 1,44 | 1,98E-03 | 4,74 | 2,08 |
| AC020978.5 | 1,44 | 1,81E-05 | 8,67 | 3,83 |
| LYPD6 | 1,44 | 5,69E-03 | 1,77 | 0,82 |
| CCDC65 | 1,44 | 6,21E-03 | 7,75 | 4,02 |
| SELENOW | 1,44 | 4,13E-04 | 274,59 | 122,87 |
| FMOD | 1,44 | 3,08E-03 | 33,07 | 15,01 |
| PWWP2B | 1,45 | 4,12E-05 | 8,66 | 3,91 |
| TMPRSS13 | 1,45 | 1,21E-03 | 10,34 | 4,93 |
| EFNA5 | 1,45 | 2,10E-05 | 5,92 | 2,34 |
| LINC00511 | 1,45 | 7,47E-05 | 8,47 | 3,90 |
| EPS8L1 | 1,45 | 6,10E-04 | 121,96 | 56,06 |
| TMEM176B | 1,45 | 2,96E-03 | 136,53 | 55,92 |
| C16orf45 | 1,46 | 2,44E-03 | 26,26 | 11,69 |
| XBP1 | 1,46 | 3,68E-05 | 482,23 | 215,82 |
| TSPAN13 | 1,46 | 5,29E-05 | 34,72 | 15,08 |
| FAM221A | 1,46 | 3,59E-03 | 9,31 | 3,75 |
| NDUFB7 | 1,46 | 8,10E-03 | 188,76 | 92,38 |
| IFT22 | 1,46 | 5,89E-03 | 51,19 | 24,58 |
| SERINC2 | 1,46 | 4,31E-03 | 75,76 | 30,79 |
| ILDR1 | 1,47 | 4,36E-04 | 14,70 | 6,30 |
| GPRC5C | 1,47 | 4,90E-06 | 93,24 | 38,56 |
| TRIM55 | 1,47 | 3,57E-03 | 3,23 | 1,71 |
| AC015813.2 | 1,47 | 6,97E-03 | 19,70 | 9,52 |
| ROGDI | 1,47 | 3,34E-05 | 17,58 | 7,44 |
| TMEM205 | 1,48 | 2,94E-07 | 196,02 | 82,66 |
| PTPRU | 1,48 | 2,32E-04 | 20,85 | 8,37 |
| CAPN5 | 1,48 | 7,01E-06 | 7,38 | 3,36 |
| ZBED6CL | 1,48 | 1,08E-03 | 5,48 | 2,44 |
| BAIAP2 | 1,48 | 7,60E-04 | 25,40 | 11,18 |
| NDUFA13 | 1,48 | 8,19E-03 | 248,79 | 120,01 |
| CES4A | 1,48 | 1,95E-04 | 9,03 | 3,59 |
| COX5B | 1,49 | 7,33E-03 | 254,45 | 119,56 |
| MKRN2OS | 1,49 | 4,17E-03 | 2,76 | 1,22 |
| TTC25 | 1,49 | 5,92E-03 | 13,03 | 6,31 |
| ZNF561-AS1 | 1,49 | 3,19E-03 | 8,25 | 3,72 |
| RAB17 | 1,49 | 9,15E-05 | 19,79 | 8,04 |
| VWA3A | 1,49 | 2,78E-03 | 15,62 | 7,20 |
| SLC45A3 | 1,49 | 6,58E-03 | 4,84 | 1,94 |
| CTNS | 1,49 | 4,10E-03 | 23,10 | 9,55 |
| ECE2 | 1,49 | 1,92E-04 | 2,58 | 1,10 |
| PRDX5 | 1,49 | 1,46E-03 | 485,95 | 226,38 |
| TMEM256 | 1,50 | 9,49E-03 | 88,47 | 42,99 |
| BCAM | 1,50 | 1,96E-03 | 37,63 | 16,39 |
| ZNHIT2 | 1,50 | 2,31E-03 | 7,64 | 3,49 |
| PRADC1 | 1,50 | 8,90E-04 | 12,54 | 5,56 |
| DPY19L2P2 | 1,50 | 4,89E-03 | 2,05 | 0,90 |
| EPB41L4B | 1,50 | 7,15E-04 | 6,35 | 2,89 |
| C1orf210 | 1,50 | 2,61E-04 | 12,12 | 5,76 |
| TSPAN6 | 1,50 | 2,02E-03 | 45,69 | 21,55 |
| TMEM47 | 1,50 | 5,10E-03 | 11,38 | 4,71 |
| BHLHE41 | 1,50 | 4,16E-03 | 30,82 | 11,38 |
| AC074212.1 | 1,51 | 4,97E-03 | 1,81 | 0,78 |
| MCF2L | 1,51 | 1,20E-05 | 10,31 | 4,33 |
| UBXN10 | 1,51 | 9,83E-04 | 16,99 | 7,74 |
| FHAD1 | 1,51 | 1,13E-04 | 11,27 | 5,28 |
| TSGA10 | 1,51 | 1,95E-03 | 12,53 | 6,25 |
| C1orf158 | 1,51 | 5,20E-03 | 4,14 | 1,85 |
| TP53TG1 | 1,51 | 1,68E-03 | 22,23 | 9,71 |
| CRIP2 | 1,52 | 1,01E-04 | 117,41 | 48,00 |
| CP | 1,52 | 6,22E-03 | 527,34 | 252,37 |
| BSCL2 | 1,52 | 6,00E-03 | 121,43 | 52,73 |
| AK7 | 1,52 | 7,00E-03 | 7,46 | 4,49 |
| HOXB4 | 1,52 | 4,07E-06 | 8,92 | 3,60 |
| RIBC1 | 1,52 | 2,99E-03 | 7,63 | 3,45 |
| OSCP1 | 1,52 | 2,47E-04 | 23,07 | 11,15 |
| HID1 | 1,53 | 2,02E-05 | 24,89 | 9,94 |
| CA13 | 1,53 | 4,90E-06 | 6,39 | 2,26 |
| CA3 | 1,53 | 4,57E-03 | 3,25 | 1,18 |
| CRIP1 | 1,53 | 4,42E-04 | 412,83 | 182,73 |
| IFFO2 | 1,53 | 7,22E-03 | 5,65 | 2,21 |
| ZNF671 | 1,53 | 7,83E-08 | 8,12 | 3,28 |
| SHMT1 | 1,53 | 7,22E-04 | 13,76 | 5,58 |
| CHN2 | 1,53 | 8,08E-05 | 7,47 | 3,27 |
| C6orf132 | 1,54 | 4,12E-04 | 14,93 | 5,75 |
| AL021328.1 | 1,54 | 8,51E-03 | 2,26 | 0,88 |
| BSPRY | 1,54 | 2,27E-04 | 11,67 | 4,60 |
| SMIM5 | 1,54 | 1,12E-03 | 6,06 | 2,42 |
| KLHDC7A | 1,54 | 1,62E-04 | 2,13 | 0,83 |
| MRPL21 | 1,54 | 4,12E-04 | 65,46 | 28,18 |
| POLE4 | 1,55 | 6,85E-05 | 93,67 | 38,94 |
| SNPH | 1,55 | 5,00E-03 | 1,78 | 0,70 |
| FAM187A | 1,55 | 4,10E-03 | 1,45 | 0,64 |
| PRR29 | 1,55 | 6,81E-04 | 6,34 | 2,60 |
| SLC9A3R2 | 1,55 | 3,49E-04 | 63,27 | 28,10 |
| EHF | 1,55 | 3,66E-05 | 96,55 | 38,81 |
| TSPAN1 | 1,55 | 2,90E-03 | 376,93 | 177,99 |
| GPX8 | 1,55 | 2,63E-03 | 42,82 | 15,27 |
| DNPH1 | 1,55 | 3,49E-04 | 55,04 | 24,57 |
| KRT8 | 1,55 | 2,14E-03 | 303,00 | 123,45 |
| DUSP23 | 1,55 | 7,40E-03 | 82,62 | 31,72 |
| SLC1A5 | 1,56 | 1,49E-04 | 42,89 | 15,61 |
| BLVRB | 1,56 | 5,16E-04 | 81,67 | 35,28 |
| SLC25A23 | 1,56 | 1,31E-04 | 11,86 | 4,79 |
| HMGA2 | 1,56 | 1,64E-03 | 1,73 | 0,68 |
| HOXB5 | 1,56 | 1,03E-06 | 18,94 | 8,10 |
| NRAV | 1,56 | 6,53E-03 | 11,96 | 5,56 |
| DMAC1 | 1,56 | 9,06E-03 | 82,55 | 31,72 |
| CALML4 | 1,57 | 2,95E-05 | 32,52 | 10,81 |
| FYB2 | 1,57 | 6,50E-04 | 15,86 | 5,99 |
| EGLN3 | 1,57 | 2,69E-03 | 21,68 | 8,14 |
| EBP | 1,57 | 2,82E-04 | 48,76 | 20,84 |
| AC016747.1 | 1,57 | 2,03E-03 | 8,30 | 3,89 |
| SH3BGRL2 | 1,57 | 1,04E-04 | 12,14 | 5,02 |
| TMEM139 | 1,57 | 1,76E-06 | 38,93 | 15,62 |
| SPR | 1,57 | 9,74E-06 | 14,11 | 5,86 |
| AP002761.4 | 1,57 | 7,46E-03 | 6,70 | 2,32 |
| NRXN3 | 1,57 | 1,00E-03 | 3,61 | 1,55 |
| FZD10 | 1,58 | 8,00E-03 | 4,46 | 1,62 |
| C11orf1 | 1,58 | 8,26E-03 | 36,91 | 16,13 |
| UQCRQ | 1,58 | 4,24E-03 | 423,38 | 184,41 |
| MMAB | 1,59 | 3,23E-04 | 31,55 | 14,22 |
| POP5 | 1,59 | 1,60E-03 | 56,48 | 25,92 |
| PRSS8 | 1,59 | 2,21E-06 | 65,86 | 26,96 |
| CLU | 1,59 | 6,30E-03 | 811,66 | 309,91 |
| HLA-DMA | 1,59 | 8,01E-05 | 159,00 | 61,17 |
| PLCE1 | 1,59 | 5,29E-04 | 7,15 | 2,99 |
| TMEM38A | 1,59 | 4,68E-04 | 1,95 | 1,01 |
| A1BG | 1,60 | 7,49E-03 | 12,00 | 4,57 |
| MIR210HG | 1,60 | 1,63E-05 | 25,85 | 8,90 |
| CADPS2 | 1,60 | 3,90E-06 | 19,71 | 7,12 |
| DTHD1 | 1,60 | 5,49E-03 | 5,74 | 2,42 |
| CFAP70 | 1,61 | 2,33E-04 | 38,92 | 16,14 |
| TEX9 | 1,61 | 3,28E-03 | 11,43 | 5,07 |
| CITED4 | 1,61 | 8,09E-04 | 16,64 | 6,61 |
| COX7C | 1,61 | 7,58E-03 | 702,48 | 298,32 |
| LARP6 | 1,61 | 2,04E-09 | 31,94 | 10,06 |
| PPFIA3 | 1,61 | 2,05E-04 | 2,74 | 1,10 |
| CREB3L1 | 1,62 | 7,68E-03 | 15,27 | 5,49 |
| SLC46A3 | 1,62 | 4,31E-05 | 25,87 | 9,03 |
| SLC44A4 | 1,62 | 2,13E-05 | 93,70 | 35,42 |
| PRR34-AS1 | 1,63 | 1,38E-04 | 32,11 | 12,46 |
| ZBED5-AS1 | 1,63 | 2,46E-04 | 12,24 | 5,32 |
| MMP15 | 1,63 | 2,50E-05 | 7,47 | 2,83 |
| AC010442.1 | 1,63 | 6,91E-03 | 19,40 | 7,09 |
| CCDC24 | 1,63 | 4,27E-07 | 17,67 | 7,10 |
| SERTM1 | 1,63 | 1,89E-03 | 1,76 | 0,66 |
| PANK1 | 1,63 | 4,95E-04 | 4,98 | 1,93 |
| SCNN1A | 1,64 | 5,00E-06 | 130,86 | 52,09 |
| ZNF667-AS1 | 1,64 | 2,01E-03 | 44,90 | 16,87 |
| GNAL | 1,64 | 1,83E-03 | 1,89 | 0,72 |
| S100A6 | 1,64 | 1,04E-05 | 3098,33 | 1226,38 |
| CAMSAP3 | 1,64 | 4,10E-08 | 6,56 | 2,82 |
| KRT19 | 1,64 | 6,11E-03 | 543,54 | 213,00 |
| URB1-AS1 | 1,65 | 1,00E-03 | 7,58 | 3,10 |
| IQCG | 1,66 | 2,89E-03 | 30,67 | 13,72 |
| FBXO15 | 1,66 | 7,67E-03 | 7,72 | 3,10 |
| VWA1 | 1,66 | 7,76E-05 | 37,81 | 14,27 |
| RAB6B | 1,66 | 1,01E-03 | 1,35 | 0,91 |
| C1orf194 | 1,66 | 1,96E-03 | 55,95 | 23,59 |
| RHBDL2 | 1,66 | 9,93E-03 | 5,49 | 2,63 |
| AK1 | 1,66 | 1,29E-05 | 65,10 | 21,69 |
| SIAH3 | 1,66 | 1,87E-03 | 1,51 | 0,56 |
| TSTD1 | 1,66 | 7,28E-05 | 106,56 | 41,59 |
| PRKAG2-AS1 | 1,66 | 4,45E-03 | 4,36 | 1,70 |
| EFCAB10 | 1,67 | 8,60E-03 | 6,71 | 2,83 |
| ERICH3 | 1,67 | 1,49E-04 | 7,20 | 2,67 |
| STX18 | 1,67 | 8,74E-03 | 249,51 | 106,63 |
| MARVELD3 | 1,67 | 2,29E-05 | 8,65 | 3,12 |
| AC004130.1 | 1,67 | 1,60E-03 | 4,59 | 1,72 |
| PYCR1 | 1,67 | 5,27E-03 | 17,64 | 8,17 |
| RSPH4A | 1,68 | 9,24E-04 | 7,56 | 3,08 |
| KIAA1211L | 1,68 | 7,57E-05 | 6,92 | 2,68 |
| SH3BP4 | 1,68 | 7,55E-04 | 22,22 | 7,39 |
| SPA17 | 1,68 | 4,14E-03 | 38,97 | 14,52 |
| RAC3 | 1,68 | 1,06E-03 | 4,78 | 1,72 |
| TMEM99 | 1,68 | 2,15E-03 | 26,34 | 11,48 |
| ASS1 | 1,68 | 9,26E-04 | 140,27 | 44,11 |
| SEL1L3 | 1,68 | 1,21E-05 | 85,07 | 26,60 |
| C9orf24 | 1,68 | 2,41E-03 | 39,55 | 17,17 |
| NUCB2 | 1,68 | 7,87E-04 | 196,09 | 79,17 |
| BTBD11 | 1,68 | 2,74E-03 | 6,39 | 2,65 |
| EFHB | 1,68 | 2,28E-03 | 5,00 | 2,25 |
| SIDT1 | 1,69 | 4,50E-04 | 4,94 | 1,85 |
| ELL3 | 1,69 | 2,81E-04 | 2,58 | 1,18 |
| HEY1 | 1,69 | 3,72E-03 | 10,01 | 3,22 |
| C5orf49 | 1,69 | 4,10E-03 | 17,50 | 6,86 |
| ENPP5 | 1,69 | 1,08E-04 | 15,45 | 6,14 |
| A4GALT | 1,69 | 1,72E-03 | 40,09 | 13,38 |
| FAM84A | 1,70 | 3,98E-04 | 7,14 | 2,77 |
| STARD10 | 1,70 | 6,85E-05 | 95,95 | 33,71 |
| COX14 | 1,70 | 1,77E-04 | 77,98 | 32,36 |
| NUS1P2 | 1,70 | 2,32E-03 | 171,37 | 58,23 |
| CYP39A1 | 1,70 | 8,00E-03 | 4,17 | 1,99 |
| ATAD3C | 1,71 | 1,40E-03 | 3,13 | 1,37 |
| QPRT | 1,71 | 1,34E-03 | 48,54 | 19,70 |
| SPAG17 | 1,71 | 1,40E-04 | 43,08 | 16,89 |
| LIPE-AS1 | 1,71 | 4,95E-03 | 24,02 | 8,58 |
| ALPK3 | 1,71 | 1,05E-05 | 3,40 | 1,39 |
| PROM1 | 1,71 | 5,75E-07 | 166,10 | 54,25 |
| NME9 | 1,71 | 6,75E-04 | 4,83 | 1,74 |
| RNF208 | 1,72 | 3,79E-04 | 3,59 | 1,35 |
| CA8 | 1,72 | 2,01E-04 | 16,69 | 5,35 |
| TMEM160 | 1,72 | 4,70E-04 | 8,63 | 3,36 |
| RUNX3 | 1,72 | 2,21E-04 | 24,66 | 8,48 |
| MARC2 | 1,72 | 1,04E-05 | 13,00 | 4,85 |
| CABYR | 1,72 | 2,95E-04 | 8,37 | 3,23 |
| KCNRG | 1,72 | 5,55E-03 | 8,62 | 3,66 |
| NHLRC4 | 1,72 | 3,46E-03 | 3,16 | 1,28 |
| DPCD | 1,72 | 4,19E-03 | 46,28 | 20,24 |
| FUCA1 | 1,72 | 5,95E-07 | 64,16 | 23,56 |
| TPTEP1 | 1,72 | 3,58E-07 | 15,47 | 5,35 |
| ALDH3A1 | 1,73 | 8,48E-04 | 2,96 | 1,05 |
| CCDC153 | 1,73 | 4,97E-03 | 10,03 | 3,59 |
| BOLA2 | 1,73 | 2,96E-03 | 5,02 | 2,01 |
| LZTS3 | 1,73 | 4,22E-09 | 15,59 | 5,51 |
| AKAP6 | 1,73 | 3,47E-04 | 1,48 | 0,68 |
| PROC | 1,73 | 4,75E-03 | 1,72 | 0,58 |
| C11orf97 | 1,73 | 3,78E-03 | 9,32 | 3,74 |
| CRYM | 1,73 | 2,43E-03 | 11,15 | 4,12 |
| GIPR | 1,74 | 1,33E-03 | 3,10 | 1,04 |
| CFAP43 | 1,74 | 1,01E-03 | 32,20 | 13,22 |
| ABCA5 | 1,74 | 2,86E-04 | 54,34 | 19,03 |
| CAPS | 1,74 | 7,01E-06 | 345,94 | 132,22 |
| ZNF540 | 1,74 | 1,37E-03 | 3,90 | 1,49 |
| CPAMD8 | 1,74 | 1,27E-04 | 24,53 | 8,57 |
| TMEM184A | 1,74 | 1,65E-03 | 10,73 | 3,76 |
| STEAP3 | 1,74 | 8,71E-03 | 35,99 | 11,48 |
| HMGB3 | 1,74 | 1,69E-03 | 51,03 | 23,15 |
| SLC38A5 | 1,75 | 1,33E-05 | 15,92 | 5,21 |
| CETN2 | 1,75 | 3,96E-03 | 131,19 | 54,35 |
| WDR38 | 1,75 | 4,90E-05 | 9,12 | 3,37 |
| GPRC5D-AS1 | 1,75 | 2,75E-03 | 7,39 | 2,90 |
| TRIM68 | 1,75 | 2,24E-04 | 12,89 | 5,46 |
| DOK7 | 1,75 | 2,26E-04 | 1,58 | 0,52 |
| DCDC2B | 1,75 | 1,85E-03 | 2,99 | 1,15 |
| NDRG2 | 1,75 | 8,79E-05 | 75,12 | 28,48 |
| TMPRSS2 | 1,76 | 1,80E-03 | 16,53 | 6,37 |
| ASRGL1 | 1,76 | 1,16E-03 | 183,11 | 68,02 |
| SH3RF2 | 1,76 | 7,53E-03 | 5,23 | 1,71 |
| LDHD | 1,76 | 3,37E-05 | 7,80 | 3,07 |
| PIH1D2 | 1,77 | 2,41E-03 | 9,70 | 3,76 |
| RF00100 | 1,77 | 6,92E-03 | 134,82 | 50,05 |
| MRPL40 | 1,77 | 2,87E-03 | 54,91 | 21,86 |
| DCDC1 | 1,77 | 7,67E-07 | 2,25 | 0,92 |
| ECI2 | 1,77 | 2,26E-03 | 81,27 | 30,14 |
| NECTIN4 | 1,77 | 4,14E-04 | 13,73 | 5,19 |
| RGPD1 | 1,78 | 1,36E-03 | 60,72 | 18,94 |
| SOBP | 1,78 | 5,91E-06 | 3,46 | 1,22 |
| ANKRD45 | 1,78 | 6,69E-04 | 3,97 | 1,48 |
| TMEM232 | 1,78 | 2,08E-03 | 7,00 | 2,89 |
| KCNQ1 | 1,78 | 7,13E-04 | 16,91 | 5,57 |
| SOX17 | 1,79 | 4,95E-04 | 25,35 | 8,43 |
| TMEM182 | 1,79 | 6,89E-05 | 2,23 | 0,95 |
| ZMYND10 | 1,79 | 1,78E-03 | 35,69 | 14,23 |
| AC104966.1 | 1,79 | 6,93E-03 | 3,69 | 1,44 |
| NAA38 | 1,79 | 2,82E-03 | 144,04 | 49,78 |
| TIGD4 | 1,79 | 6,50E-04 | 1,59 | 0,58 |
| RASAL1 | 1,79 | 3,14E-04 | 10,33 | 3,71 |
| HOXB8 | 1,79 | 1,21E-08 | 24,82 | 8,61 |
| EMP2 | 1,80 | 3,34E-12 | 31,10 | 12,01 |
| C11orf88 | 1,81 | 6,88E-03 | 59,83 | 23,73 |
| STXBP6 | 1,81 | 7,26E-06 | 32,51 | 8,82 |
| CDC42EP5 | 1,81 | 3,83E-04 | 30,44 | 11,61 |
| SELENBP1 | 1,81 | 3,18E-05 | 52,23 | 18,66 |
| AP006284.1 | 1,81 | 1,89E-04 | 5,70 | 2,11 |
| SMAGP | 1,81 | 7,16E-06 | 21,15 | 7,41 |
| VMO1 | 1,82 | 2,73E-03 | 29,33 | 9,81 |
| LRRC34 | 1,82 | 1,10E-03 | 4,76 | 1,87 |
| THEM6 | 1,82 | 6,71E-06 | 15,19 | 5,51 |
| MGST2 | 1,82 | 2,11E-04 | 118,47 | 43,82 |
| CYB5A | 1,82 | 8,79E-05 | 140,16 | 46,87 |
| AC008267.5 | 1,82 | 2,89E-03 | 4,93 | 1,53 |
| SLC27A2 | 1,82 | 1,42E-08 | 4,78 | 1,55 |
| MCEE | 1,82 | 6,93E-04 | 22,82 | 8,28 |
| CCDC160 | 1,82 | 9,11E-05 | 3,22 | 1,11 |
| SERHL | 1,83 | 7,27E-03 | 2,51 | 0,96 |
| AC007255.1 | 1,83 | 8,00E-03 | 13,48 | 4,09 |
| ANKFN1 | 1,83 | 1,45E-03 | 5,78 | 1,83 |
| TTC22 | 1,83 | 2,41E-04 | 7,40 | 2,34 |
| AC010719.1 | 1,83 | 1,63E-05 | 7,03 | 2,31 |
| MISP3 | 1,84 | 6,46E-05 | 3,59 | 1,04 |
| MORN2 | 1,84 | 1,01E-04 | 72,04 | 26,71 |
| HIST1H2BJ | 1,84 | 1,17E-03 | 6,07 | 1,85 |
| LINC02345 | 1,84 | 6,08E-03 | 6,26 | 2,14 |
| C22orf23 | 1,84 | 5,03E-03 | 1,65 | 0,70 |
| NLRP2 | 1,84 | 4,45E-03 | 16,15 | 5,05 |
| BST2 | 1,84 | 6,14E-06 | 156,73 | 53,67 |
| ABCA10 | 1,85 | 2,97E-03 | 8,20 | 1,96 |
| KCTD14 | 1,85 | 1,11E-06 | 20,41 | 7,11 |
| CFAP206 | 1,85 | 7,41E-03 | 7,41 | 2,93 |
| AC139769.1 | 1,85 | 5,46E-04 | 6,16 | 2,24 |
| AP002360.1 | 1,85 | 4,90E-03 | 9,52 | 3,26 |
| MRAP2 | 1,85 | 5,20E-03 | 2,73 | 0,96 |
| TJP3 | 1,85 | 1,47E-07 | 42,34 | 10,73 |
| B9D1 | 1,85 | 8,00E-05 | 24,54 | 8,67 |
| RAB38 | 1,85 | 6,17E-04 | 6,59 | 2,22 |
| ELF3 | 1,85 | 1,32E-06 | 192,64 | 64,22 |
| ATP6V0E2 | 1,86 | 6,50E-07 | 8,85 | 2,95 |
| C19orf18 | 1,86 | 2,27E-04 | 3,71 | 1,29 |
| BICDL2 | 1,86 | 1,46E-06 | 22,12 | 6,67 |
| PDCL3P4 | 1,86 | 6,34E-05 | 8,40 | 2,79 |
| PEG10 | 1,86 | 2,50E-06 | 12,35 | 3,95 |
| FBXO27 | 1,86 | 1,26E-03 | 2,63 | 0,76 |
| EYA2 | 1,86 | 1,20E-03 | 49,34 | 15,26 |
| MLPH | 1,86 | 3,02E-05 | 152,06 | 41,84 |
| CBLC | 1,87 | 7,01E-06 | 8,27 | 2,83 |
| DNAAF3 | 1,87 | 4,67E-05 | 8,98 | 3,16 |
| C22orf15 | 1,87 | 1,49E-03 | 3,12 | 0,77 |
| FOXA2 | 1,87 | 3,85E-05 | 6,55 | 2,21 |
| PLEKHS1 | 1,87 | 5,13E-03 | 83,67 | 25,07 |
| PAM | 1,87 | 3,08E-08 | 606,21 | 182,79 |
| RAB25 | 1,87 | 4,65E-06 | 78,39 | 23,79 |
| BOLA2B | 1,87 | 3,29E-04 | 77,76 | 25,64 |
| GOT1 | 1,87 | 2,16E-05 | 72,45 | 25,66 |
| NRG4 | 1,87 | 8,19E-03 | 2,22 | 0,65 |
| STX19 | 1,87 | 3,08E-03 | 5,60 | 2,00 |
| CILP | 1,88 | 7,82E-05 | 64,06 | 20,48 |
| AC027281.1 | 1,88 | 8,46E-03 | 4,63 | 1,66 |
| KIF9 | 1,88 | 1,32E-04 | 35,12 | 11,33 |
| CHST9 | 1,88 | 6,50E-04 | 4,40 | 1,48 |
| TMEM176A | 1,88 | 2,41E-08 | 83,02 | 26,49 |
| ST6GAL1 | 1,88 | 6,84E-07 | 92,78 | 28,14 |
| ANKRD65 | 1,88 | 2,22E-03 | 14,12 | 4,57 |
| WDR78 | 1,89 | 5,58E-03 | 13,64 | 5,23 |
| ZNF404 | 1,89 | 5,52E-04 | 6,33 | 2,00 |
| DEFB1 | 1,89 | 1,16E-03 | 950,88 | 337,89 |
| NME5 | 1,89 | 2,53E-03 | 22,85 | 8,77 |
| CMAHP | 1,89 | 9,92E-04 | 48,87 | 15,87 |
| SEMA3B | 1,89 | 6,95E-04 | 66,76 | 19,22 |
| TMEM229B | 1,89 | 2,69E-03 | 7,43 | 2,42 |
| LCA5L | 1,90 | 4,06E-05 | 5,51 | 2,05 |
| ZMYND12 | 1,90 | 3,96E-04 | 5,96 | 1,89 |
| KDELR3 | 1,90 | 2,01E-04 | 32,90 | 11,61 |
| TMEM246 | 1,90 | 1,88E-05 | 7,51 | 2,47 |
| C20orf85 | 1,91 | 3,21E-04 | 75,54 | 26,57 |
| LOXL4 | 1,91 | 1,54E-03 | 10,49 | 3,39 |
| ADGRV1 | 1,91 | 1,06E-05 | 1,61 | 0,46 |
| NQO1 | 1,91 | 4,91E-03 | 41,41 | 13,65 |
| RHPN1 | 1,91 | 7,41E-10 | 10,40 | 2,98 |
| AL441992.1 | 1,91 | 1,67E-03 | 8,77 | 2,98 |
| SPEF1 | 1,92 | 5,42E-04 | 6,77 | 2,34 |
| ALDH1L1 | 1,92 | 3,71E-04 | 23,89 | 7,58 |
| RPP25 | 1,92 | 1,90E-05 | 4,55 | 1,54 |
| RIMBP2 | 1,92 | 4,85E-03 | 5,87 | 1,99 |
| PLPP2 | 1,92 | 1,64E-09 | 103,56 | 33,30 |
| IQUB | 1,92 | 2,80E-03 | 2,46 | 0,94 |
| PLEKHB1 | 1,93 | 8,63E-09 | 11,36 | 4,62 |
| ARFGEF3 | 1,93 | 2,20E-08 | 4,32 | 1,32 |
| SMOC1 | 1,93 | 4,35E-04 | 2,50 | 0,74 |
| GCNT3 | 1,93 | 2,61E-04 | 50,61 | 17,12 |
| STOML3 | 1,93 | 7,83E-03 | 8,75 | 3,27 |
| CRACR2B | 1,94 | 1,02E-06 | 13,65 | 4,72 |
| GOLGA2P10 | 1,95 | 2,74E-03 | 9,55 | 3,01 |
| AL355075.4 | 1,95 | 8,39E-03 | 30,24 | 9,89 |
| PDE3A | 1,96 | 2,26E-04 | 5,49 | 1,75 |
| AC007009.1 | 1,96 | 2,75E-03 | 11,06 | 3,71 |
| CPNE7 | 1,96 | 6,36E-03 | 4,03 | 1,11 |
| ECT2L | 1,96 | 3,09E-04 | 3,15 | 0,87 |
| ZBBX | 1,96 | 2,96E-03 | 27,67 | 11,16 |
| TMEM190 | 1,97 | 8,98E-03 | 23,49 | 6,95 |
| ACADL | 1,97 | 7,16E-06 | 6,77 | 2,15 |
| PPP1R42 | 1,97 | 8,00E-03 | 8,26 | 2,76 |
| AC005083.1 | 1,97 | 8,10E-03 | 16,15 | 5,67 |
| MFSD3 | 1,97 | 2,80E-06 | 7,80 | 2,48 |
| KLHDC7B | 1,97 | 9,44E-03 | 4,82 | 1,44 |
| C1orf87 | 1,97 | 7,43E-03 | 9,74 | 3,55 |
| HOXA-AS2 | 1,98 | 1,48E-05 | 13,27 | 3,68 |
| HYKK | 1,98 | 2,53E-03 | 2,31 | 0,77 |
| LINC00240 | 1,98 | 8,93E-05 | 25,17 | 7,64 |
| PLEKHG7 | 1,98 | 5,76E-08 | 8,67 | 2,96 |
| TNFRSF6B | 1,98 | 7,90E-03 | 16,49 | 4,59 |
| ZNF233 | 1,98 | 6,80E-04 | 2,01 | 0,67 |
| AC093627.4 | 1,99 | 4,53E-08 | 4,90 | 1,74 |
| GOLGA2P7 | 1,99 | 4,74E-04 | 48,70 | 14,30 |
| ATP6V1B1 | 1,99 | 5,45E-03 | 30,71 | 9,47 |
| AL355916.1 | 2,00 | 3,73E-04 | 14,98 | 4,64 |
| PLA2G10 | 2,00 | 3,41E-03 | 4,42 | 1,52 |
| THSD4 | 2,00 | 5,94E-03 | 117,57 | 31,74 |
| CFTR | 2,01 | 4,24E-04 | 38,37 | 12,03 |
| CUTALP | 2,01 | 8,76E-08 | 17,17 | 5,72 |
| AL390719.2 | 2,01 | 1,37E-05 | 19,61 | 5,42 |
| AC099676.1 | 2,02 | 3,13E-03 | 2,45 | 0,72 |
| C4orf19 | 2,02 | 3,22E-07 | 17,88 | 5,46 |
| S100A14 | 2,02 | 2,62E-03 | 81,08 | 26,67 |
| CFAP45 | 2,02 | 2,64E-04 | 21,75 | 7,08 |
| FAM229B | 2,02 | 1,96E-03 | 48,59 | 16,15 |
| DMKN | 2,02 | 4,53E-13 | 169,13 | 46,18 |
| DTX4 | 2,03 | 5,55E-09 | 31,89 | 10,43 |
| MDH1B | 2,03 | 1,02E-04 | 8,41 | 2,97 |
| NUDT8 | 2,03 | 7,92E-05 | 6,54 | 1,84 |
| PLEKHG6 | 2,03 | 2,51E-03 | 3,86 | 1,23 |
| MSX1 | 2,03 | 3,90E-05 | 97,80 | 29,52 |
| MROH9 | 2,04 | 5,33E-03 | 2,32 | 0,96 |
| TNFRSF14-AS1 | 2,04 | 3,10E-07 | 33,63 | 7,95 |
| MCRIP2 | 2,04 | 1,67E-06 | 23,00 | 6,59 |
| DYNLRB2 | 2,04 | 9,31E-03 | 29,94 | 9,77 |
| RGS17 | 2,04 | 4,82E-07 | 2,06 | 0,66 |
| FAM47E | 2,04 | 1,71E-07 | 8,73 | 3,17 |
| WDR63 | 2,05 | 8,74E-03 | 7,35 | 3,27 |
| ZDHHC11B | 2,05 | 4,94E-03 | 1,64 | 0,46 |
| PDE4C | 2,05 | 3,46E-05 | 21,59 | 3,01 |
| CFAP126 | 2,05 | 6,88E-03 | 39,18 | 13,85 |
| CACNB1 | 2,05 | 2,05E-05 | 15,63 | 4,60 |
| CCDC173 | 2,05 | 5,31E-03 | 3,83 | 1,17 |
| NELL2 | 2,06 | 1,41E-03 | 3,81 | 1,28 |
| SPATA17 | 2,06 | 3,96E-04 | 13,06 | 3,80 |
| CSRP2 | 2,06 | 9,45E-06 | 48,14 | 14,09 |
| RASSF7 | 2,06 | 1,95E-07 | 60,41 | 18,70 |
| CNR1 | 2,06 | 5,04E-05 | 2,88 | 0,68 |
| C6 | 2,06 | 3,64E-03 | 8,58 | 2,93 |
| SLC5A1 | 2,06 | 1,20E-10 | 19,15 | 5,41 |
| FAM83E | 2,07 | 9,53E-05 | 42,33 | 12,12 |
| TMEM125 | 2,07 | 9,48E-15 | 34,41 | 9,70 |
| VWA5B2 | 2,07 | 2,06E-03 | 2,24 | 0,54 |
| TCTEX1D1 | 2,08 | 2,50E-05 | 13,68 | 3,78 |
| PROM2 | 2,08 | 6,17E-03 | 28,93 | 7,44 |
| SMIM22 | 2,08 | 2,11E-07 | 202,35 | 61,77 |
| SORCS2 | 2,08 | 2,21E-04 | 2,73 | 0,66 |
| PRSS22 | 2,09 | 1,82E-03 | 56,52 | 17,27 |
| LRRN1 | 2,09 | 3,16E-04 | 12,40 | 3,33 |
| SLC6A20 | 2,09 | 4,61E-04 | 7,03 | 1,92 |
| INPP5J | 2,09 | 1,38E-07 | 5,50 | 1,66 |
| TMPRSS3 | 2,09 | 3,87E-08 | 24,52 | 6,08 |
| ST6GALNAC1 | 2,10 | 1,14E-08 | 152,16 | 38,33 |
| ZNF582-AS1 | 2,10 | 1,80E-04 | 4,19 | 1,24 |
| STRA6 | 2,10 | 5,11E-05 | 131,13 | 45,06 |
| ASIC1 | 2,10 | 1,27E-04 | 2,57 | 0,69 |
| TMEM238 | 2,10 | 4,75E-04 | 3,72 | 1,05 |
| PIH1D3 | 2,10 | 3,61E-03 | 4,57 | 1,56 |
| EAF2 | 2,10 | 1,54E-03 | 10,84 | 3,29 |
| ADGB | 2,10 | 2,04E-03 | 7,43 | 2,54 |
| FAXC | 2,10 | 3,50E-04 | 2,03 | 0,39 |
| LMNTD1 | 2,10 | 7,01E-03 | 1,77 | 0,88 |
| BCAS1 | 2,10 | 8,59E-04 | 12,30 | 3,92 |
| LINC01139 | 2,11 | 7,55E-04 | 6,04 | 1,79 |
| GPR39 | 2,11 | 7,06E-05 | 5,28 | 1,34 |
| TNNT2 | 2,11 | 1,63E-03 | 3,74 | 1,13 |
| SMKR1 | 2,11 | 2,82E-03 | 2,26 | 0,67 |
| EPN3 | 2,11 | 4,28E-06 | 4,67 | 1,76 |
| SNTN | 2,11 | 4,44E-04 | 34,59 | 11,21 |
| ACSF2 | 2,11 | 3,33E-07 | 64,61 | 15,50 |
| AGR3 | 2,11 | 7,39E-03 | 199,63 | 70,07 |
| RIC3 | 2,11 | 1,93E-05 | 10,37 | 2,61 |
| C16orf71 | 2,12 | 1,79E-05 | 2,01 | 0,71 |
| IFNE | 2,12 | 1,67E-03 | 2,63 | 0,71 |
| AC044860.1 | 2,12 | 5,61E-03 | 7,87 | 2,27 |
| GMDS | 2,12 | 1,53E-08 | 33,28 | 9,20 |
| SYT7 | 2,13 | 5,81E-07 | 2,19 | 0,51 |
| CASC1 | 2,13 | 2,26E-04 | 8,89 | 2,79 |
| CEP70 | 2,13 | 7,49E-04 | 53,54 | 17,06 |
| FXYD3 | 2,13 | 5,86E-05 | 567,70 | 157,98 |
| PORCN | 2,14 | 8,78E-05 | 20,08 | 5,35 |
| RNF183 | 2,14 | 3,87E-08 | 10,19 | 2,51 |
| MLF1 | 2,14 | 2,09E-03 | 25,24 | 8,22 |
| LINC01550 | 2,14 | 9,74E-03 | 1,59 | 0,54 |
| ZNF750 | 2,14 | 5,77E-03 | 4,64 | 0,95 |
| VTCN1 | 2,15 | 3,95E-04 | 79,01 | 23,61 |
| MUC4 | 2,15 | 5,47E-04 | 94,40 | 21,49 |
| CHST6 | 2,15 | 1,09E-03 | 15,98 | 3,80 |
| MPPED2 | 2,15 | 1,01E-08 | 29,95 | 8,30 |
| TNFRSF18 | 2,15 | 5,72E-05 | 37,16 | 9,16 |
| CREB3L4 | 2,15 | 6,03E-10 | 41,44 | 12,60 |
| SLC47A1 | 2,15 | 5,15E-03 | 13,60 | 3,03 |
| SNHG19 | 2,15 | 2,07E-04 | 141,05 | 43,74 |
| AC008771.1 | 2,15 | 4,56E-05 | 8,57 | 2,27 |
| PDE8B | 2,16 | 1,47E-04 | 5,53 | 1,41 |
| ZNF204P | 2,16 | 6,75E-07 | 28,20 | 7,78 |
| MAP2K6 | 2,16 | 6,19E-05 | 69,89 | 19,45 |
| SAXO2 | 2,17 | 1,08E-05 | 12,60 | 3,83 |
| SPAG6 | 2,17 | 5,56E-06 | 13,07 | 3,98 |
| TRIM31 | 2,17 | 2,87E-03 | 11,69 | 3,18 |
| BMP3 | 2,19 | 4,32E-04 | 1,70 | 0,47 |
| CDRT1 | 2,19 | 3,01E-03 | 1,71 | 0,54 |
| BEX2 | 2,19 | 4,18E-07 | 15,29 | 4,21 |
| GALNT12 | 2,19 | 1,55E-07 | 11,25 | 3,14 |
| SERTAD4-AS1 | 2,19 | 1,00E-07 | 11,76 | 3,40 |
| TNFSF11 | 2,20 | 2,29E-03 | 1,99 | 0,48 |
| LRRC10B | 2,20 | 7,01E-06 | 5,61 | 1,52 |
| PRR15 | 2,20 | 6,24E-06 | 59,32 | 15,20 |
| C10orf95 | 2,20 | 8,00E-05 | 1,79 | 0,48 |
| FAM81B | 2,20 | 3,03E-03 | 17,93 | 6,22 |
| VWA2 | 2,20 | 1,21E-04 | 3,57 | 0,86 |
| SLC16A12 | 2,20 | 7,16E-03 | 6,50 | 1,72 |
| APOBEC4 | 2,21 | 1,40E-03 | 6,50 | 1,77 |
| HOXB9 | 2,21 | 2,13E-05 | 11,88 | 2,92 |
| NEK10 | 2,21 | 2,56E-04 | 7,60 | 2,27 |
| STEAP2 | 2,22 | 3,18E-07 | 9,31 | 1,77 |
| CCL28 | 2,22 | 1,11E-04 | 21,19 | 5,17 |
| B3GAT1 | 2,22 | 1,70E-03 | 1,75 | 0,39 |
| PIFO | 2,23 | 1,30E-03 | 52,22 | 16,59 |
| TFCP2L1 | 2,23 | 5,35E-04 | 34,92 | 8,27 |
| LDLRAD1 | 2,24 | 8,05E-09 | 15,95 | 4,05 |
| NXF3 | 2,24 | 9,44E-09 | 4,00 | 0,90 |
| AL645608.6 | 2,24 | 1,10E-03 | 10,69 | 3,02 |
| IL17RE | 2,24 | 6,54E-06 | 3,61 | 0,94 |
| CCNA1 | 2,24 | 5,11E-04 | 15,62 | 3,84 |
| MIA | 2,25 | 2,45E-04 | 10,45 | 2,56 |
| AL109936.2 | 2,26 | 7,58E-03 | 1,79 | 0,48 |
| LRRIQ1 | 2,26 | 2,89E-03 | 12,95 | 3,04 |
| TTC29 | 2,26 | 2,24E-03 | 5,90 | 1,76 |
| AL121899.1 | 2,26 | 8,74E-04 | 8,07 | 2,23 |
| AP000688.2 | 2,26 | 2,72E-03 | 2,70 | 0,66 |
| FAM81A | 2,27 | 1,23E-04 | 6,22 | 1,74 |
| SLC4A11 | 2,27 | 6,50E-04 | 24,17 | 5,74 |
| AC106872.5 | 2,27 | 4,26E-03 | 2,25 | 0,64 |
| PDZK1IP1 | 2,27 | 3,95E-05 | 164,52 | 41,58 |
| CAPN12 | 2,28 | 3,57E-10 | 27,86 | 7,79 |
| SCOC-AS1 | 2,28 | 5,67E-04 | 4,32 | 0,84 |
| FAM189A2 | 2,28 | 5,15E-05 | 85,64 | 20,14 |
| LINC01091 | 2,28 | 8,59E-05 | 3,54 | 0,78 |
| AMN | 2,29 | 1,14E-08 | 4,33 | 1,29 |
| OMG | 2,30 | 2,74E-03 | 55,86 | 15,62 |
| MORN5 | 2,30 | 4,72E-04 | 32,72 | 9,25 |
| ANO5 | 2,30 | 9,61E-07 | 2,00 | 0,56 |
| AC113349.1 | 2,31 | 6,11E-03 | 3,82 | 0,98 |
| PLAC8 | 2,31 | 2,07E-08 | 139,59 | 32,48 |
| ATP6V1C2 | 2,31 | 3,70E-05 | 53,37 | 13,68 |
| FZD8 | 2,32 | 1,74E-08 | 5,74 | 1,35 |
| C3 | 2,32 | 8,83E-08 | 2944,64 | 487,44 |
| ANKRD35 | 2,32 | 5,17E-03 | 29,09 | 7,61 |
| BIK | 2,32 | 1,81E-06 | 10,00 | 2,22 |
| RBPMS-AS1 | 2,32 | 1,01E-03 | 3,00 | 0,78 |
| ESPN | 2,32 | 1,94E-05 | 48,96 | 11,58 |
| ZNF812P | 2,33 | 9,66E-03 | 3,59 | 0,95 |
| POF1B | 2,33 | 1,27E-03 | 3,30 | 0,75 |
| VIPR1 | 2,33 | 5,42E-07 | 3,32 | 0,96 |
| ARL4D | 2,33 | 1,84E-05 | 37,70 | 8,81 |
| KIAA2012 | 2,33 | 3,24E-04 | 3,05 | 0,77 |
| NPTX2 | 2,34 | 8,37E-05 | 11,60 | 2,63 |
| ZNF503-AS2 | 2,34 | 6,58E-07 | 3,36 | 0,79 |
| ENTPD3 | 2,34 | 2,59E-05 | 33,04 | 8,28 |
| AC021242.3 | 2,35 | 8,59E-05 | 5,85 | 1,43 |
| FUT3 | 2,35 | 1,17E-03 | 23,91 | 5,40 |
| SPTBN2 | 2,35 | 3,69E-06 | 33,91 | 7,01 |
| PHGDH | 2,35 | 1,04E-05 | 11,38 | 2,92 |
| CKMT1B | 2,36 | 8,42E-05 | 6,67 | 1,86 |
| GPX2 | 2,36 | 5,19E-03 | 4,15 | 1,09 |
| LINC00284 | 2,36 | 3,55E-07 | 67,40 | 16,80 |
| PACRG | 2,36 | 3,39E-04 | 9,68 | 2,77 |
| FLRT3 | 2,37 | 2,37E-08 | 10,96 | 2,41 |
| RNF39 | 2,37 | 7,78E-11 | 10,58 | 2,47 |
| ENPP3 | 2,37 | 8,26E-03 | 71,91 | 18,86 |
| RARRES2 | 2,38 | 3,25E-04 | 205,95 | 52,58 |
| AC011294.1 | 2,39 | 2,80E-05 | 3,60 | 0,84 |
| BMPR1B | 2,40 | 9,09E-09 | 17,21 | 4,03 |
| TMEM106C | 2,40 | 3,66E-05 | 79,15 | 20,99 |
| SDR16C5 | 2,40 | 4,27E-05 | 8,45 | 1,95 |
| C2orf40 | 2,40 | 8,74E-03 | 4,58 | 1,04 |
| VSIG2 | 2,40 | 8,38E-12 | 41,70 | 9,63 |
| SCNN1G | 2,40 | 3,53E-04 | 32,41 | 7,06 |
| ZNF208 | 2,41 | 2,00E-05 | 4,26 | 1,00 |
| CYP2B7P | 2,41 | 2,63E-03 | 24,60 | 5,04 |
| ADRB2 | 2,42 | 1,12E-04 | 19,13 | 4,15 |
| MLXIPL | 2,42 | 1,06E-05 | 3,27 | 0,65 |
| MPV17L | 2,43 | 2,02E-11 | 7,08 | 1,50 |
| TMEM116 | 2,44 | 1,37E-05 | 17,09 | 4,33 |
| AC026704.1 | 2,44 | 1,79E-03 | 2,14 | 0,48 |
| CDH26 | 2,44 | 2,34E-08 | 11,07 | 2,44 |
| DUOXA1 | 2,44 | 1,01E-03 | 23,35 | 6,04 |
| CRISP2 | 2,45 | 2,04E-03 | 9,77 | 2,61 |
| GGT6 | 2,45 | 2,52E-04 | 6,39 | 1,50 |
| METTL27 | 2,46 | 1,87E-03 | 5,70 | 1,40 |
| PNMA6A | 2,46 | 1,35E-04 | 3,81 | 0,92 |
| SLC15A2 | 2,46 | 1,51E-07 | 51,65 | 14,33 |
| FBXO16 | 2,46 | 3,82E-07 | 10,88 | 3,05 |
| SMIM6 | 2,47 | 1,63E-03 | 17,93 | 4,80 |
| FAM107A | 2,47 | 1,60E-03 | 10,80 | 2,47 |
| BCL2L14 | 2,47 | 2,64E-03 | 3,12 | 0,86 |
| CLIC5 | 2,48 | 1,57E-14 | 22,07 | 4,58 |
| MISP | 2,48 | 1,37E-05 | 17,20 | 3,55 |
| AC019117.2 | 2,48 | 1,59E-03 | 31,14 | 6,95 |
| HRASLS2 | 2,49 | 4,38E-04 | 8,77 | 2,20 |
| BCL2L15 | 2,49 | 2,02E-11 | 35,42 | 6,92 |
| ENKUR | 2,49 | 6,66E-04 | 19,10 | 4,78 |
| CCDC181 | 2,50 | 4,06E-04 | 4,34 | 1,07 |
| TCEA3 | 2,50 | 2,45E-10 | 50,31 | 11,62 |
| FABP6 | 2,50 | 9,80E-06 | 18,78 | 4,37 |
| PRSS50 | 2,51 | 4,69E-04 | 2,34 | 0,50 |
| PLXNA4 | 2,52 | 6,69E-04 | 2,13 | 0,44 |
| AC004816.1 | 2,52 | 5,48E-04 | 6,37 | 1,04 |
| LINC01770 | 2,52 | 4,58E-03 | 3,61 | 0,73 |
| MIR200CHG | 2,54 | 1,00E-06 | 64,25 | 14,90 |
| SHISA2 | 2,54 | 8,82E-03 | 2,20 | 0,39 |
| AP002358.1 | 2,55 | 4,30E-06 | 4,38 | 0,84 |
| C9orf135 | 2,56 | 2,02E-03 | 18,80 | 4,52 |
| FGFBP1 | 2,56 | 3,12E-03 | 42,37 | 8,55 |
| AC026304.1 | 2,56 | 1,39E-03 | 4,36 | 1,01 |
| TNNI3 | 2,57 | 1,43E-03 | 3,41 | 0,81 |
| DDIT4L | 2,57 | 6,43E-04 | 20,39 | 4,13 |
| CHAC1 | 2,57 | 5,84E-04 | 5,14 | 1,16 |
| FUT6 | 2,58 | 5,75E-11 | 12,26 | 2,72 |
| AKAP14 | 2,58 | 2,48E-04 | 19,07 | 4,85 |
| ECEL1P2 | 2,58 | 1,69E-05 | 8,93 | 1,67 |
| C16orf89 | 2,58 | 3,43E-04 | 12,09 | 2,29 |
| ACE2 | 2,59 | 3,34E-03 | 4,46 | 0,83 |
| CATSPERD | 2,59 | 1,14E-03 | 8,29 | 1,97 |
| IRX6 | 2,59 | 3,05E-04 | 3,90 | 0,69 |
| CLIC6 | 2,60 | 4,23E-11 | 20,42 | 4,10 |
| CAPN6 | 2,60 | 4,22E-03 | 3,77 | 0,82 |
| PLG | 2,60 | 4,86E-04 | 4,89 | 1,16 |
| PRSS27 | 2,60 | 8,62E-03 | 19,90 | 4,33 |
| MB | 2,62 | 1,11E-04 | 4,24 | 0,84 |
| C4BPB | 2,62 | 1,90E-06 | 12,28 | 1,99 |
| SIX1 | 2,62 | 2,03E-11 | 19,94 | 3,64 |
| AC243964.2 | 2,63 | 7,38E-03 | 1,84 | 0,32 |
| AC084866.1 | 2,63 | 3,57E-03 | 55,48 | 10,69 |
| AC013264.1 | 2,64 | 9,45E-04 | 17,70 | 4,18 |
| GALNT6 | 2,64 | 4,83E-08 | 37,43 | 8,26 |
| AL136088.1 | 2,64 | 1,45E-03 | 4,62 | 0,93 |
| RGS7BP | 2,65 | 2,10E-05 | 1,97 | 0,40 |
| DMBT1 | 2,65 | 5,18E-04 | 21,84 | 3,76 |
| STEAP1 | 2,66 | 1,45E-10 | 14,40 | 2,89 |
| TMPRSS11D | 2,67 | 9,40E-03 | 28,97 | 6,31 |
| GLDN | 2,67 | 6,00E-07 | 2,88 | 0,49 |
| CAPSL | 2,67 | 4,19E-05 | 39,48 | 8,59 |
| CAPN13 | 2,67 | 2,35E-05 | 66,32 | 13,14 |
| LCN12 | 2,68 | 8,00E-05 | 51,40 | 9,44 |
| SPATA4 | 2,68 | 1,37E-03 | 4,04 | 0,95 |
| AL390778.2 | 2,68 | 3,28E-04 | 1,94 | 0,36 |
| GCHFR | 2,69 | 7,03E-10 | 75,57 | 14,43 |
| TMEM212 | 2,70 | 1,41E-04 | 5,08 | 1,15 |
| REN | 2,70 | 6,69E-04 | 3,46 | 0,65 |
| FAM183A | 2,71 | 1,23E-04 | 94,68 | 21,16 |
| CCDC60 | 2,71 | 5,95E-08 | 4,72 | 0,94 |
| LRRC18 | 2,71 | 6,00E-05 | 3,07 | 0,62 |
| SPINT1-AS1 | 2,71 | 4,91E-10 | 59,05 | 11,97 |
| KCNJ12 | 2,72 | 9,29E-05 | 2,21 | 0,39 |
| C19orf33 | 2,72 | 6,70E-07 | 260,59 | 52,66 |
| FOLR1 | 2,72 | 1,29E-09 | 118,07 | 21,76 |
| CYP4X1 | 2,72 | 3,02E-05 | 35,67 | 6,41 |
| ABCC6P1 | 2,73 | 9,84E-07 | 1,81 | 0,50 |
| FOXI2 | 2,73 | 4,89E-07 | 1,82 | 0,32 |
| ERBB4 | 2,73 | 3,03E-06 | 3,98 | 0,83 |
| C1orf189 | 2,73 | 1,41E-03 | 19,55 | 4,36 |
| LMO7-AS1 | 2,74 | 3,22E-03 | 2,77 | 0,50 |
| CDH12 | 2,74 | 1,27E-06 | 7,32 | 1,27 |
| SEMA3B-AS1 | 2,74 | 1,31E-03 | 11,83 | 2,37 |
| HOXB-AS3 | 2,75 | 4,22E-05 | 55,24 | 10,78 |
| RMST | 2,77 | 7,60E-07 | 19,83 | 3,72 |
| TNNC1 | 2,77 | 7,88E-05 | 35,29 | 6,74 |
| PPP1R1A | 2,77 | 5,50E-05 | 2,41 | 0,55 |
| PCP2 | 2,77 | 3,53E-05 | 4,22 | 0,79 |
| DUOX1 | 2,78 | 5,72E-05 | 47,67 | 7,71 |
| SORD2P | 2,80 | 4,29E-04 | 80,75 | 14,18 |
| CYSRT1 | 2,80 | 6,05E-03 | 8,91 | 1,76 |
| SULT2B1 | 2,80 | 4,62E-07 | 18,08 | 3,16 |
| ALG1L | 2,80 | 9,26E-04 | 4,93 | 0,96 |
| MMEL1 | 2,80 | 1,83E-09 | 31,36 | 5,92 |
| HHLA2 | 2,81 | 7,04E-03 | 1,67 | 0,40 |
| FOXA3 | 2,82 | 5,96E-11 | 5,17 | 0,87 |
| PRR15L | 2,83 | 3,56E-09 | 23,78 | 4,37 |
| SLC5A5 | 2,83 | 2,46E-03 | 14,97 | 2,64 |
| COL9A2 | 2,83 | 6,31E-21 | 35,75 | 5,98 |
| PIP | 2,83 | 6,39E-05 | 13,53 | 2,70 |
| S100A2 | 2,84 | 3,72E-04 | 317,07 | 47,85 |
| CD8B | 2,85 | 2,03E-11 | 60,48 | 10,39 |
| CKMT1A | 2,87 | 2,24E-08 | 7,10 | 1,32 |
| AL357093.2 | 2,87 | 1,62E-07 | 52,03 | 9,43 |
| UBXN10-AS1 | 2,88 | 1,85E-04 | 10,75 | 1,94 |
| ATP6V0A4 | 2,89 | 3,00E-03 | 2,63 | 0,55 |
| AC130456.2 | 2,89 | 7,86E-04 | 7,28 | 0,61 |
| TMEM40 | 2,90 | 4,59E-03 | 15,52 | 2,44 |
| RARRES1 | 2,90 | 2,05E-06 | 359,75 | 56,35 |
| SLC4A4 | 2,90 | 2,34E-08 | 14,38 | 2,32 |
| MAL | 2,91 | 8,78E-03 | 73,86 | 12,66 |
| JPH3 | 2,92 | 1,00E-03 | 2,84 | 0,36 |
| CFB | 2,92 | 6,03E-10 | 842,90 | 104,76 |
| LINC00261 | 2,92 | 3,23E-07 | 8,83 | 1,31 |
| IL19 | 2,93 | 6,96E-04 | 48,30 | 8,99 |
| TMPRSS11E | 2,93 | 6,17E-03 | 28,70 | 5,48 |
| RAB26 | 2,94 | 2,91E-09 | 8,46 | 1,08 |
| SNHG25 | 2,95 | 2,26E-03 | 359,51 | 23,38 |
| CCNO | 2,95 | 6,19E-14 | 15,57 | 2,49 |
| TACR1 | 2,95 | 1,05E-03 | 6,56 | 0,83 |
| C2orf54 | 2,96 | 7,38E-05 | 8,75 | 1,27 |
| SRGAP3-AS2 | 2,97 | 1,84E-05 | 10,86 | 1,81 |
| WFDC2 | 2,97 | 1,07E-11 | 8028,97 | 1220,37 |
| SCGB2A1 | 2,98 | 2,80E-06 | 2133,85 | 364,11 |
| SLC25A47P1 | 2,98 | 7,90E-04 | 4,78 | 0,73 |
| ACY3 | 2,98 | 1,16E-11 | 18,08 | 2,83 |
| IGHA2 | 2,98 | 5,83E-03 | 31,90 | 4,70 |
| AQP5 | 2,98 | 3,39E-06 | 15,50 | 2,55 |
| AP002373.1 | 2,99 | 2,91E-03 | 2,11 | 0,37 |
| FAM3B | 2,99 | 4,46E-08 | 7,89 | 1,22 |
| LINC01133 | 3,00 | 8,95E-04 | 7,47 | 1,26 |
| MZB1 | 3,00 | 3,28E-04 | 34,94 | 5,59 |
| ITGA10 | 3,00 | 2,20E-04 | 10,10 | 1,72 |
| LINC01571 | 3,01 | 1,42E-03 | 2,99 | 0,52 |
| BMPR1B-AS1 | 3,02 | 7,18E-04 | 4,39 | 0,68 |
| KAZALD1 | 3,02 | 3,40E-10 | 19,28 | 2,89 |
| LYPD6B | 3,03 | 9,90E-07 | 18,03 | 2,91 |
| SMIM1 | 3,03 | 1,12E-06 | 8,07 | 1,28 |
| TMEM213 | 3,03 | 1,99E-03 | 1,92 | 0,27 |
| CXCL17 | 3,03 | 3,24E-05 | 100,16 | 17,02 |
| SLC5A8 | 3,04 | 2,03E-11 | 24,18 | 3,46 |
| PRRX2 | 3,05 | 8,42E-13 | 10,88 | 1,63 |
| LINC01508 | 3,05 | 8,13E-07 | 7,98 | 1,15 |
| MUC1 | 3,05 | 3,05E-09 | 1301,31 | 188,84 |
| CEACAM7 | 3,06 | 4,45E-03 | 123,73 | 20,27 |
| ESM1 | 3,07 | 3,00E-03 | 9,71 | 1,32 |
| PIGR | 3,09 | 4,09E-30 | 977,89 | 139,98 |
| CPB1 | 3,11 | 4,71E-04 | 1,84 | 0,70 |
| ZNF876P | 3,11 | 1,25E-08 | 6,91 | 0,95 |
| SCN1A | 3,11 | 4,82E-07 | 1,87 | 0,34 |
| ZG16B | 3,11 | 1,66E-03 | 112,43 | 16,75 |
| SPDEF | 3,15 | 1,91E-10 | 54,43 | 6,93 |
| PCP4L1 | 3,15 | 1,70E-04 | 9,37 | 1,12 |
| EPPIN | 3,16 | 3,12E-06 | 3,53 | 0,46 |
| FUT2 | 3,17 | 9,48E-15 | 62,98 | 6,94 |
| AC010280.1 | 3,18 | 8,73E-03 | 5,07 | 0,70 |
| SCNN1B | 3,18 | 2,13E-15 | 91,69 | 12,49 |
| CFH | 3,18 | 4,36E-10 | 182,02 | 27,23 |
| GOLT1A | 3,18 | 2,95E-08 | 7,45 | 1,09 |
| AL355353.1 | 3,19 | 8,32E-04 | 5,84 | 0,76 |
| AL355601.1 | 3,21 | 6,53E-04 | 5,20 | 0,80 |
| SHH | 3,21 | 3,77E-11 | 12,28 | 1,58 |
| AC025154.2 | 3,21 | 3,10E-07 | 4,19 | 0,57 |
| SPRR2D | 3,21 | 3,04E-03 | 195,54 | 30,38 |
| CHGA | 3,21 | 1,09E-05 | 3,75 | 0,59 |
| SPRR3 | 3,21 | 2,13E-03 | 749,41 | 117,18 |
| LINC01829 | 3,22 | 5,94E-03 | 5,41 | 0,72 |
| ERN2 | 3,23 | 8,94E-25 | 68,22 | 7,99 |
| SPRR2A | 3,23 | 3,00E-03 | 338,97 | 51,71 |
| KRT13 | 3,24 | 4,85E-03 | 552,81 | 46,00 |
| AC080013.4 | 3,25 | 2,08E-06 | 17,99 | 2,37 |
| AC008035.1 | 3,30 | 6,79E-03 | 2,41 | 0,33 |
| SERPINB3 | 3,30 | 2,50E-03 | 652,34 | 90,10 |
| CHAD | 3,30 | 5,16E-09 | 14,42 | 1,60 |
| TMPRSS11B | 3,34 | 2,46E-04 | 14,88 | 2,13 |
| BPIFA1 | 3,37 | 1,89E-03 | 49,75 | 6,84 |
| SCGB1D4 | 3,38 | 1,78E-03 | 216,80 | 31,65 |
| RNF175 | 3,38 | 7,82E-15 | 7,96 | 0,85 |
| SORD | 3,38 | 1,87E-08 | 215,85 | 32,28 |
| MUC5B | 3,39 | 1,87E-13 | 579,23 | 67,74 |
| FAM69C | 3,40 | 1,63E-08 | 1,99 | 0,19 |
| FAM3D | 3,40 | 7,71E-12 | 42,03 | 6,06 |
| PON3 | 3,44 | 3,28E-05 | 9,90 | 1,18 |
| SPINK5 | 3,45 | 1,54E-03 | 31,45 | 3,73 |
| SLC46A2 | 3,45 | 8,01E-05 | 14,60 | 1,53 |
| TCN1 | 3,45 | 1,26E-07 | 493,07 | 59,20 |
| OR7E47P | 3,46 | 1,73E-05 | 4,52 | 0,57 |
| FGA | 3,46 | 2,44E-03 | 17,07 | 1,62 |
| PTPRT | 3,47 | 3,20E-19 | 2,14 | 0,24 |
| SERPINA3 | 3,49 | 1,77E-05 | 1022,07 | 112,48 |
| VSTM2L | 3,49 | 7,41E-10 | 25,96 | 2,90 |
| AL390198.1 | 3,50 | 2,48E-03 | 6,15 | 0,75 |
| SPRR1B | 3,53 | 1,47E-03 | 159,69 | 18,57 |
| AC008105.2 | 3,55 | 6,80E-03 | 2,43 | 0,23 |
| TMEM211 | 3,55 | 1,93E-04 | 8,30 | 0,99 |
| SCGB1D2 | 3,55 | 2,23E-04 | 3176,21 | 374,53 |
| GLYATL2 | 3,57 | 2,02E-20 | 41,99 | 3,86 |
| AC109462.1 | 3,58 | 9,27E-03 | 4,05 | 0,43 |
| AC012236.1 | 3,59 | 7,21E-05 | 1,93 | 0,32 |
| MESP1 | 3,64 | 1,50E-06 | 18,92 | 2,15 |
| LCN2 | 3,65 | 3,09E-15 | 5942,99 | 592,54 |
| ZNF98 | 3,65 | 3,51E-04 | 1,77 | 0,26 |
| CNFN | 3,66 | 2,22E-04 | 133,37 | 14,10 |
| ENTPD8 | 3,66 | 3,90E-08 | 5,13 | 0,58 |
| BPIFB1 | 3,67 | 1,46E-12 | 2263,85 | 223,51 |
| SLPI | 3,72 | 1,52E-14 | 41508,99 | 4007,59 |
| C10orf99 | 3,74 | 2,08E-03 | 9,81 | 0,92 |
| CHST4 | 3,74 | 4,27E-07 | 5,64 | 0,52 |
| LTF | 3,76 | 4,28E-05 | 918,37 | 68,92 |
| TEX26 | 3,80 | 1,03E-08 | 5,81 | 0,79 |
| JCHAIN | 3,81 | 1,54E-03 | 98,00 | 7,64 |
| TFF3 | 3,82 | 1,85E-13 | 2334,12 | 206,31 |
| NUPR2 | 3,82 | 1,48E-04 | 3,64 | 0,30 |
| AL035701.1 | 3,84 | 5,84E-04 | 3,65 | 0,33 |
| AGR2 | 3,87 | 3,55E-11 | 4842,66 | 473,05 |
| MUC5AC | 3,92 | 9,87E-12 | 71,77 | 5,33 |
| MUC6 | 3,95 | 8,05E-20 | 8,72 | 1,02 |
| CST2 | 3,96 | 1,41E-03 | 2,41 | 0,23 |
| AMY1C | 3,96 | 6,76E-06 | 63,11 | 5,55 |
| TFF1 | 3,97 | 1,59E-09 | 36,62 | 3,12 |
| TSPAN19 | 3,99 | 8,59E-05 | 4,59 | 0,55 |
| SAA4 | 4,13 | 2,76E-17 | 46,70 | 3,29 |
| SAA2 | 4,16 | 1,47E-08 | 1357,23 | 88,25 |
| SFRP2 | 4,16 | 1,81E-08 | 6,16 | 0,41 |
| SLC51B | 4,17 | 2,00E-06 | 17,23 | 1,27 |
| SAA2-SAA4 | 4,17 | 3,57E-14 | 68,50 | 4,72 |
| SBSN | 4,24 | 3,95E-04 | 9,77 | 0,54 |
| CHP2 | 4,24 | 1,20E-05 | 11,69 | 0,86 |
| S100A7 | 4,27 | 5,72E-04 | 850,51 | 64,20 |
| SPRR2E | 4,30 | 1,84E-03 | 146,62 | 10,95 |
| DEFB4A | 4,33 | 1,32E-04 | 77,10 | 6,16 |
| GSTA1 | 4,41 | 6,24E-06 | 11,91 | 0,78 |
| CA10 | 4,42 | 1,27E-11 | 12,30 | 0,75 |
| WFDC6 | 4,44 | 1,05E-07 | 9,50 | 0,53 |
| SAA1 | 4,47 | 2,64E-17 | 2045,18 | 115,77 |
| DAW1 | 4,49 | 9,74E-09 | 8,39 | 0,53 |
| SPRR1A | 4,49 | 1,25E-03 | 24,83 | 1,59 |
| HP | 4,66 | 5,73E-06 | 348,82 | 21,35 |
| CD8B2 | 4,75 | 1,40E-04 | 3,66 | 0,23 |
| SPINK7 | 4,84 | 2,02E-04 | 38,55 | 1,85 |
| LINC02300 | 4,91 | 2,44E-03 | 1,94 | 0,07 |
| KRTDAP | 5,17 | 3,13E-04 | 13,18 | 0,48 |
| AC108752.1 | 5,49 | 3,40E-03 | 6,09 | 0,15 |
| AC007849.1 | 5,60 | 5,06E-04 | 2,59 | 0,05 |
| TFF2 | 6,18 | 5,12E-05 | 4,63 | 0,10 |
| AC008870.3 | 6,91 | 4,33E-06 | 3,98 | 0,00 |
