# Supplementary material for: The cervical transcriptome changes during the menstrual cycle but does not predict the window of implantation
Source: Front Reprod Health. 2023 Jul 14;5:1224919. doi: 10.3389/frph.2023.1224919 (PMC10375708; doi:10.3389/frph.2023.1224919)
Supplement: Supplementary file 8 [file Table5.docx]

| **Supplementary table 5.** Differentially expressed genes (Log_2_Fold change > 1, FDR<0.01 and average values of transcript per million (TPM) per group) between LH+7 and HRC: P+5 groups. | | | | |
| --- | --- | --- | --- | --- |
| **Gene_symbol** | **log2FC** | **FDR** | **TPM_P+5** | **TPM_LH+7** |
| B4GALNT4 | -3,92 | 1,23E-03 | 0,32 | 2,76 |
| RNA5-8SP6 | -3,82 | 2,67E-06 | 39,97 | 382,72 |
| AC013268.1 | -3,51 | 8,04E-03 | 0,85 | 4,43 |
| SFRP4 | -3,51 | 1,27E-04 | 1,13 | 10,76 |
| HTD2 | -3,42 | 1,37E-03 | 0,29 | 2,69 |
| DIO2 | -3,38 | 9,98E-04 | 1,32 | 7,27 |
| AL031710.1 | -3,09 | 4,65E-03 | 0,73 | 3,91 |
| SNHG25 | -3,08 | 2,90E-04 | 1,79 | 359,51 |
| AL590560.2 | -3,01 | 6,20E-05 | 1,02 | 6,94 |
| AC008870.3 | -2,99 | 1,97E-04 | 0,66 | 3,98 |
| COX7A1 | -2,91 | 1,69E-03 | 1,07 | 5,38 |
| ZNF321P | -2,90 | 7,55E-05 | 2,38 | 8,68 |
| TMEM211 | -2,88 | 3,54E-03 | 1,60 | 8,30 |
| CXCL14 | -2,83 | 7,25E-03 | 3,10 | 16,30 |
| LINC01139 | -2,82 | 4,65E-06 | 1,03 | 6,04 |
| MXRA8 | -2,77 | 9,50E-03 | 1,55 | 7,37 |
| AEBP1 | -2,76 | 1,95E-03 | 0,91 | 4,78 |
| SHISA2 | -2,76 | 4,35E-03 | 0,36 | 2,20 |
| MT1F | -2,75 | 8,66E-03 | 9,78 | 46,04 |
|  |  |  |  |  |
| CRISP3 | -2,71 | 2,27E-03 | 32,63 | 164,62 |
| GSTA1 | -2,66 | 6,90E-03 | 3,01 | 11,91 |
| TNNI3 | -2,66 | 5,11E-05 | 0,60 | 3,41 |
| AC073896.4 | -2,64 | 7,90E-04 | 0,86 | 4,33 |
| PTGDS | -2,64 | 5,10E-03 | 8,61 | 43,13 |
| SLITRK6 | -2,62 | 6,04E-03 | 0,46 | 2,06 |
| MT1E | -2,60 | 8,41E-04 | 12,25 | 59,18 |
| MFAP2 | -2,57 | 8,46E-03 | 0,42 | 1,82 |
| BGN | -2,55 | 5,10E-03 | 0,54 | 2,60 |
| WISP2 | -2,54 | 7,52E-04 | 0,44 | 2,08 |
| TNNT1 | -2,53 | 1,40E-03 | 0,78 | 2,84 |
| AC025154.2 | -2,44 | 8,55E-05 | 1,14 | 4,19 |
| DPY19L2 | -2,43 | 1,33E-03 | 0,94 | 3,49 |
| GRIA3 | -2,40 | 9,38E-04 | 0,55 | 1,96 |
| RNU1-27P | -2,39 | 2,49E-03 | 40,15 | 120,44 |
| RNU1-2 | -2,39 | 2,49E-03 | 40,15 | 120,44 |
| RNU1-4 | -2,39 | 2,49E-03 | 40,15 | 120,44 |
| AC090772.3 | -2,39 | 9,91E-04 | 0,72 | 2,99 |
| LINC01315 | -2,37 | 2,28E-03 | 0,50 | 2,00 |
| AP000254.1 | -2,36 | 1,99E-03 | 0,67 | 2,68 |
| OR2A9P | -2,35 | 7,28E-03 | 0,70 | 2,16 |
| DIO3OS | -2,34 | 2,60E-03 | 0,33 | 2,88 |
| COL16A1 | -2,32 | 1,62E-04 | 1,26 | 4,78 |
| FGF9 | -2,30 | 4,61E-03 | 1,13 | 3,93 |
| AC239800.2 | -2,27 | 7,26E-03 | 0,48 | 1,68 |
| SNORA73B | -2,27 | 9,76E-03 | 5,51 | 19,62 |
| AC067838.1 | -2,25 | 6,96E-05 | 1,27 | 4,96 |
| SCGB1D4 | -2,24 | 5,64E-03 | 68,38 | 216,80 |
| RNU4-2 | -2,23 | 7,25E-03 | 39,62 | 127,68 |
| AC007228.2 | -2,23 | 4,22E-03 | 0,53 | 1,62 |
| FLRT2 | -2,17 | 2,90E-06 | 1,41 | 4,49 |
| WFDC1 | -2,17 | 4,79E-03 | 0,50 | 1,78 |
| SULT1A3 | -2,15 | 1,25E-08 | 8,33 | 28,85 |
| AL357093.2 | -2,14 | 1,56E-04 | 14,11 | 52,03 |
| TCIM | -2,13 | 5,70E-03 | 24,18 | 76,29 |
| MMP11 | -2,13 | 4,75E-03 | 0,59 | 2,25 |
| AC026304.1 | -2,12 | 2,61E-03 | 1,29 | 4,36 |
| KRBOX1 | -2,11 | 3,24E-03 | 1,30 | 4,41 |
| NTRK3 | -2,11 | 9,41E-03 | 0,46 | 2,76 |
| AL354836.1 | -2,10 | 4,50E-03 | 1,41 | 4,86 |
| MGP | -2,07 | 3,47E-03 | 15,75 | 53,39 |
| UBXN10-AS1 | -2,07 | 4,88E-03 | 3,57 | 10,75 |
| C1orf189 | -2,04 | 3,86E-03 | 6,57 | 19,55 |
| AC245884.8 | -2,04 | 7,30E-03 | 0,68 | 2,28 |
| WFDC6 | -2,04 | 2,12E-03 | 2,90 | 9,50 |
| RMRP | -2,04 | 2,42E-03 | 8,99 | 26,34 |
| DEFB1 | -2,00 | 6,91E-03 | 321,57 | 950,88 |
| SMIM1 | -2,00 | 3,68E-04 | 2,68 | 8,07 |
| LINC02482 | -1,99 | 3,65E-03 | 3,82 | 10,72 |
| FAM229B | -1,99 | 5,81E-04 | 15,60 | 48,59 |
| SNHG19 | -1,99 | 1,80E-04 | 50,28 | 141,05 |
| RF00100 | -1,99 | 3,26E-03 | 40,60 | 134,82 |
| AL355353.1 | -1,97 | 8,98E-03 | 1,78 | 5,84 |
| AGR3 | -1,96 | 3,42E-03 | 73,71 | 199,63 |
| PRSS50 | -1,95 | 8,49E-03 | 0,80 | 2,34 |
| AL355075.4 | -1,94 | 6,69E-03 | 10,21 | 30,24 |
| AC055822.1 | -1,94 | 3,49E-04 | 1,98 | 6,79 |
| AC073896.2 | -1,93 | 8,68E-03 | 2,78 | 7,71 |
| BDH2 | -1,92 | 1,43E-03 | 26,54 | 76,98 |
| AQP5 | -1,91 | 2,04E-04 | 5,41 | 15,50 |
| RARRES2 | -1,91 | 6,83E-03 | 73,58 | 205,95 |
| RPL9P7 | -1,91 | 1,56E-03 | 11,19 | 30,84 |
| SNORD3A | -1,91 | 6,05E-03 | 18,53 | 46,77 |
| CCDC181 | -1,89 | 3,09E-03 | 1,59 | 4,34 |
| PLA2G10 | -1,88 | 2,78E-03 | 1,69 | 4,42 |
| SPATA4 | -1,88 | 4,13E-03 | 1,61 | 4,04 |
| AC080013.4 | -1,87 | 5,94E-03 | 6,24 | 17,99 |
| AKR1C3 | -1,86 | 3,44E-04 | 14,19 | 40,92 |
| TRO | -1,85 | 3,28E-03 | 1,27 | 3,38 |
| AL163636.2 | -1,84 | 2,24E-03 | 4,30 | 11,59 |
| PDE4C | -1,84 | 3,50E-03 | 7,93 | 21,59 |
| AKAP14 | -1,84 | 2,06E-03 | 7,78 | 19,07 |
| SCOC-AS1 | -1,83 | 3,02E-03 | 1,36 | 4,32 |
| DYNLRB2 | -1,82 | 9,35E-03 | 11,85 | 29,94 |
| PPARGC1A | -1,82 | 9,27E-04 | 1,30 | 3,61 |
| GSTA4 | -1,81 | 2,21E-03 | 5,37 | 14,40 |
| AC239809.3 | -1,80 | 6,42E-03 | 1,39 | 3,06 |
| PPP1R1A | -1,80 | 1,12E-03 | 0,97 | 2,41 |
| FAM183A | -1,79 | 2,45E-03 | 37,88 | 94,68 |
| AL645608.6 | -1,79 | 5,54E-03 | 4,04 | 10,69 |
| SYNJ2BP-COX16 | -1,78 | 6,89E-03 | 6,29 | 15,60 |
| C19orf33 | -1,78 | 6,06E-03 | 100,96 | 260,59 |
| DAW1 | -1,77 | 2,48E-03 | 3,24 | 8,39 |
| RPL32P29 | -1,77 | 2,89E-03 | 6,63 | 16,63 |
| EFEMP1 | -1,76 | 4,45E-03 | 4,05 | 10,94 |
| CYGB | -1,75 | 2,73E-03 | 1,05 | 2,70 |
| CAPSL | -1,74 | 3,78E-03 | 16,01 | 39,48 |
| ADIRF | -1,74 | 5,00E-03 | 13,34 | 41,45 |
| SGCE | -1,73 | 5,82E-03 | 6,51 | 14,20 |
| KLHL41 | -1,73 | 4,13E-03 | 0,65 | 1,82 |
| GPM6B | -1,72 | 6,21E-03 | 1,15 | 2,90 |
| CFI | -1,72 | 6,06E-03 | 45,81 | 110,00 |
| SERTM1 | -1,72 | 2,00E-03 | 0,64 | 1,76 |
| DYDC2 | -1,71 | 5,26E-03 | 11,47 | 29,62 |
| BEX2 | -1,71 | 2,86E-04 | 6,08 | 15,29 |
| NME5 | -1,70 | 5,06E-03 | 9,89 | 22,85 |
| AL590235.1 | -1,69 | 3,07E-03 | 0,67 | 1,84 |
| WDR63 | -1,69 | 4,67E-03 | 2,98 | 7,35 |
| CEP70 | -1,69 | 6,70E-03 | 22,55 | 53,54 |
| CASC1 | -1,69 | 2,19E-03 | 4,02 | 8,89 |
| C11orf74 | -1,68 | 2,34E-03 | 11,55 | 27,09 |
| MROH9 | -1,66 | 4,56E-03 | 0,98 | 2,32 |
| DDIT4L | -1,66 | 8,35E-03 | 8,19 | 20,39 |
| H1FX-AS1 | -1,65 | 7,72E-03 | 0,81 | 2,53 |
| ERBB4 | -1,65 | 6,54E-03 | 1,70 | 3,98 |
| AAMDC | -1,64 | 1,87E-03 | 16,00 | 37,52 |
| AL121899.1 | -1,64 | 4,78E-03 | 3,41 | 8,07 |
| AP002360.1 | -1,64 | 9,25E-03 | 4,07 | 9,52 |
| RBP1 | -1,64 | 6,23E-03 | 27,67 | 66,00 |
| LINC00240 | -1,63 | 2,58E-03 | 10,92 | 25,17 |
| TNXB | -1,63 | 8,60E-03 | 0,76 | 1,90 |
| AP001372.2 | -1,63 | 7,97E-03 | 1,40 | 3,15 |
| TMEM232 | -1,63 | 1,82E-03 | 3,05 | 7,00 |
| RPL9 | -1,63 | 3,54E-03 | 770,33 | 1811,34 |
| TMEM99 | -1,62 | 3,42E-03 | 12,09 | 26,34 |
| RAB26 | -1,62 | 7,96E-03 | 3,05 | 8,46 |
| PINLYP | -1,62 | 9,76E-03 | 5,17 | 12,35 |
| RNF175 | -1,62 | 2,17E-03 | 3,28 | 7,96 |
| ENKUR | -1,61 | 6,25E-03 | 7,99 | 19,10 |
| ADGB | -1,61 | 5,94E-03 | 3,34 | 7,43 |
| RIMKLB | -1,61 | 5,94E-03 | 42,63 | 126,07 |
| NME7 | -1,61 | 4,35E-03 | 9,83 | 19,87 |
| CABCOCO1 | -1,61 | 3,79E-03 | 2,94 | 7,31 |
| FLRT3 | -1,61 | 6,11E-03 | 4,77 | 10,96 |
| ARMCX7P | -1,59 | 8,20E-03 | 5,74 | 13,65 |
| ZBBX | -1,58 | 7,81E-03 | 13,07 | 27,67 |
| S100A13 | -1,58 | 8,79E-03 | 111,68 | 253,65 |
| FBXO17 | -1,58 | 8,09E-03 | 0,86 | 1,90 |
| PPP1R42 | -1,58 | 6,57E-03 | 3,90 | 8,26 |
| FKBP10 | -1,58 | 6,06E-03 | 3,06 | 6,56 |
| DYNC2LI1 | -1,57 | 5,61E-03 | 15,20 | 32,07 |
| GPRC5D-AS1 | -1,57 | 1,62E-03 | 3,55 | 7,39 |
| AC015813.2 | -1,57 | 1,18E-03 | 8,28 | 19,70 |
| CETN2 | -1,56 | 3,53E-03 | 60,12 | 131,19 |
| DMAC1 | -1,56 | 3,19E-03 | 28,71 | 82,55 |
| NEK11 | -1,55 | 1,66E-03 | 6,48 | 13,89 |
| FAM3B | -1,53 | 6,61E-03 | 3,31 | 7,89 |
| ERP27 | -1,52 | 8,47E-03 | 18,43 | 37,72 |
| AL591895.1 | -1,51 | 7,71E-03 | 7,84 | 16,20 |
| PPIL3 | -1,51 | 8,77E-05 | 15,85 | 35,84 |
| PIFO | -1,51 | 4,06E-03 | 23,14 | 52,22 |
| MGST2 | -1,51 | 1,28E-03 | 53,74 | 118,47 |
| ZNF667-AS1 | -1,50 | 4,96E-03 | 16,95 | 44,90 |
| MORN5 | -1,50 | 6,29E-03 | 15,58 | 32,72 |
| CHST9 | -1,50 | 8,33E-03 | 1,95 | 4,40 |
| NDN | -1,50 | 2,04E-04 | 2,81 | 6,43 |
| ZNF415 | -1,50 | 7,93E-03 | 4,45 | 8,42 |
| MDH1B | -1,49 | 3,24E-03 | 4,23 | 8,41 |
| AC108062.1 | -1,49 | 8,60E-03 | 0,98 | 2,24 |
| AC016876.1 | -1,49 | 4,47E-03 | 4,38 | 8,79 |
| ME3 | -1,48 | 1,06E-03 | 3,71 | 7,95 |
| IMMP1L | -1,48 | 4,05E-03 | 13,42 | 35,84 |
| APOBEC4 | -1,48 | 6,11E-03 | 3,10 | 6,50 |
| DPCD | -1,48 | 4,95E-03 | 23,61 | 46,28 |
| C11orf1 | -1,47 | 7,13E-03 | 17,98 | 36,91 |
| C19orf18 | -1,47 | 5,19E-03 | 1,73 | 3,71 |
| ZNF771 | -1,47 | 4,20E-03 | 0,97 | 2,26 |
| MT-CO3 | -1,46 | 3,83E-04 | 20053,72 | 42823,68 |
| LRRC49 | -1,45 | 6,90E-03 | 3,98 | 7,51 |
| SPEF2 | -1,45 | 9,33E-04 | 8,88 | 16,79 |
| IFT81 | -1,45 | 2,76E-03 | 4,88 | 9,86 |
| CFAP53 | -1,45 | 1,66E-03 | 5,49 | 11,34 |
| HIST1H4J | -1,45 | 5,35E-03 | 6,88 | 14,12 |
| TMEM116 | -1,44 | 5,38E-03 | 8,30 | 17,09 |
| MCEE | -1,44 | 8,26E-03 | 11,46 | 22,82 |
| TEKT1 | -1,44 | 5,19E-03 | 12,35 | 25,07 |
| RIDA | -1,43 | 4,23E-03 | 6,96 | 14,43 |
| TDRP | -1,43 | 4,82E-03 | 4,92 | 11,06 |
| ERICH6-AS1 | -1,43 | 6,64E-03 | 1,97 | 4,49 |
| TMEM14A | -1,43 | 5,50E-03 | 7,31 | 14,97 |
| TP53TG1 | -1,43 | 2,63E-03 | 11,22 | 22,23 |
| NUDT12 | -1,43 | 9,00E-03 | 5,15 | 10,50 |
| DNAH12 | -1,42 | 4,45E-03 | 10,00 | 22,19 |
| MT-ATP8 | -1,42 | 2,19E-03 | 23613,56 | 44947,10 |
| BAG2 | -1,42 | 5,07E-03 | 2,49 | 5,70 |
| NAA38 | -1,42 | 5,50E-03 | 63,71 | 144,04 |
| ZNF177 | -1,42 | 5,82E-03 | 3,01 | 6,39 |
| UQCRQ | -1,41 | 3,03E-03 | 201,79 | 423,38 |
| ZNF32 | -1,41 | 2,12E-03 | 21,71 | 44,49 |
| TCTEX1D2 | -1,41 | 5,06E-03 | 9,79 | 19,64 |
| ZNF30 | -1,41 | 5,67E-03 | 1,74 | 3,64 |
| C4orf47 | -1,41 | 3,72E-04 | 6,60 | 13,51 |
| COX7C | -1,41 | 4,50E-03 | 326,15 | 702,48 |
| MRPL40 | -1,41 | 3,79E-03 | 26,77 | 54,91 |
| MT-ND3 | -1,41 | 2,85E-03 | 11378,19 | 22418,64 |
| ZNF165 | -1,40 | 1,51E-04 | 1,75 | 3,68 |
| ENTPD3-AS1 | -1,40 | 8,98E-03 | 3,67 | 7,73 |
| MRPL48 | -1,40 | 6,71E-03 | 17,72 | 37,22 |
| IFT22 | -1,39 | 7,65E-03 | 25,33 | 51,19 |
| MORN2 | -1,39 | 2,01E-03 | 36,22 | 72,04 |
| MIPOL1 | -1,39 | 3,37E-03 | 0,96 | 2,09 |
| ATP5ME | -1,39 | 2,43E-03 | 288,49 | 564,35 |
| MT-CO2 | -1,39 | 1,98E-03 | 13590,57 | 27480,44 |
| CETN3 | -1,39 | 6,73E-04 | 14,43 | 29,51 |
| KCTD6 | -1,39 | 2,35E-04 | 6,94 | 13,92 |
| COX16 | -1,38 | 7,28E-03 | 30,53 | 65,77 |
| KIF9 | -1,38 | 1,96E-03 | 15,39 | 35,12 |
| TSGA10 | -1,38 | 4,60E-03 | 5,81 | 12,53 |
| NUDT7 | -1,37 | 3,82E-03 | 2,55 | 4,20 |
| TCEAL9 | -1,37 | 2,97E-03 | 28,24 | 56,76 |
| MT-ATP6 | -1,37 | 3,19E-03 | 15225,47 | 30534,42 |
| SPA17 | -1,37 | 9,30E-03 | 18,14 | 38,97 |
| EEF1AKMT1 | -1,36 | 1,53E-03 | 4,12 | 8,40 |
| DPH5 | -1,36 | 3,26E-03 | 12,82 | 28,59 |
| ECT2L | -1,35 | 8,24E-03 | 1,70 | 3,15 |
| NDUFA4 | -1,35 | 5,10E-03 | 63,46 | 169,70 |
| NPHP1 | -1,34 | 4,23E-03 | 6,07 | 12,88 |
| MTATP6P1 | -1,34 | 4,67E-03 | 2202,94 | 4330,67 |
| SNTN | -1,34 | 8,77E-03 | 17,86 | 34,59 |
| ARL6 | -1,33 | 2,76E-03 | 3,14 | 7,14 |
| HMGN5 | -1,33 | 4,76E-03 | 5,75 | 12,35 |
| SPAG16 | -1,33 | 7,91E-03 | 12,33 | 21,88 |
| CCDC102A | -1,32 | 2,32E-03 | 0,90 | 1,98 |
| IQCK | -1,32 | 8,48E-03 | 14,41 | 25,59 |
| HYLS1 | -1,32 | 8,90E-03 | 1,96 | 4,04 |
| EFCAB7 | -1,32 | 8,11E-03 | 3,10 | 6,15 |
| LXN | -1,31 | 7,56E-03 | 19,70 | 37,20 |
| MT-ND4L | -1,31 | 3,78E-04 | 13660,71 | 25512,01 |
| UQCC3 | -1,31 | 5,51E-03 | 4,15 | 8,34 |
| MNAT1 | -1,31 | 2,20E-03 | 13,88 | 25,47 |
| CCNB1IP1 | -1,30 | 1,48E-03 | 12,63 | 24,26 |
| YAE1D1 | -1,30 | 3,19E-03 | 8,31 | 15,87 |
| KLHDC9 | -1,30 | 8,24E-03 | 7,08 | 15,04 |
| C11orf88 | -1,30 | 7,62E-03 | 32,32 | 59,83 |
| GSTO2 | -1,30 | 7,61E-03 | 9,09 | 25,06 |
| AC084036.1 | -1,30 | 3,53E-03 | 4,19 | 8,43 |
| CCDC148 | -1,30 | 3,94E-03 | 1,30 | 2,42 |
| TMEM14C | -1,30 | 5,25E-04 | 58,58 | 115,69 |
| CCDC58 | -1,30 | 5,38E-03 | 13,46 | 26,12 |
| ARL3 | -1,30 | 6,31E-03 | 6,37 | 11,95 |
| AC008771.1 | -1,29 | 9,53E-03 | 4,71 | 8,57 |
| TMEM256 | -1,29 | 3,56E-03 | 47,97 | 88,47 |
| POP5 | -1,29 | 3,71E-03 | 30,82 | 56,48 |
| ZNF443 | -1,29 | 5,83E-03 | 3,08 | 5,74 |
| BEX3 | -1,29 | 6,90E-03 | 34,60 | 67,59 |
| TEX9 | -1,29 | 9,61E-03 | 6,15 | 11,43 |
| GKAP1 | -1,29 | 1,86E-05 | 2,58 | 5,14 |
| LCA5L | -1,29 | 1,52E-03 | 3,26 | 5,51 |
| EXTL2 | -1,28 | 3,64E-03 | 4,62 | 8,38 |
| PCAT19 | -1,28 | 6,44E-03 | 8,81 | 15,97 |
| UQCRH | -1,28 | 6,19E-03 | 143,12 | 265,68 |
| AMY2B | -1,28 | 2,43E-03 | 33,62 | 64,56 |
| IFT74 | -1,28 | 9,55E-03 | 7,89 | 14,12 |
| MAP9 | -1,27 | 7,42E-03 | 2,55 | 4,80 |
| SRR | -1,27 | 4,08E-04 | 3,33 | 6,90 |
| CEP112 | -1,27 | 3,97E-03 | 4,52 | 7,05 |
| FBXO15 | -1,27 | 8,49E-04 | 3,99 | 7,72 |
| PRR34-AS1 | -1,27 | 1,69E-03 | 19,60 | 32,11 |
| TNFRSF19 | -1,26 | 6,07E-03 | 3,34 | 6,13 |
| MTND2P28 | -1,26 | 6,57E-04 | 191,58 | 373,78 |
| ZNF540 | -1,26 | 7,16E-03 | 2,05 | 3,90 |
| BBOF1 | -1,26 | 9,41E-03 | 12,66 | 20,91 |
| MRPS33 | -1,26 | 4,95E-03 | 39,46 | 73,10 |
| TMEM38A | -1,25 | 1,87E-03 | 1,01 | 1,95 |
| SPATA17 | -1,25 | 5,20E-03 | 7,33 | 13,06 |
| NDUFS5 | -1,24 | 7,94E-03 | 195,16 | 348,50 |
| SEM1 | -1,24 | 2,89E-03 | 102,52 | 174,03 |
| PDZD11 | -1,23 | 4,21E-03 | 20,17 | 36,84 |
| AL450405.1 | -1,23 | 5,57E-03 | 19,18 | 37,68 |
| CLHC1 | -1,23 | 8,80E-03 | 4,32 | 8,05 |
| CXXC5 | -1,23 | 7,16E-03 | 17,74 | 28,74 |
| MEST | -1,23 | 3,42E-03 | 2,19 | 3,32 |
| SPATA7 | -1,22 | 9,68E-03 | 3,66 | 6,38 |
| COX6C | -1,22 | 8,93E-03 | 183,27 | 338,08 |
| ENDOG | -1,22 | 2,90E-03 | 6,55 | 12,02 |
| MT-CYB | -1,22 | 2,34E-03 | 7679,19 | 14050,53 |
| MT-ND4 | -1,21 | 1,20E-03 | 12924,16 | 23887,80 |
| RPL22P1 | -1,21 | 4,70E-03 | 113,95 | 205,70 |
| FMC1 | -1,21 | 1,64E-03 | 16,62 | 28,85 |
| IQCG | -1,20 | 9,41E-03 | 17,51 | 30,67 |
| MRPL36 | -1,20 | 1,02E-03 | 24,64 | 44,87 |
| ZNF702P | -1,20 | 2,67E-03 | 5,05 | 7,86 |
| GTF2H5 | -1,20 | 9,74E-03 | 2,39 | 4,24 |
| NDUFB2 | -1,20 | 7,25E-03 | 135,73 | 243,94 |
| NME3 | -1,20 | 2,05E-03 | 24,73 | 47,66 |
| MRPL13 | -1,19 | 5,66E-03 | 21,88 | 39,21 |
| CYB5A | -1,19 | 8,04E-03 | 74,76 | 140,16 |
| DHRS3 | -1,19 | 2,65E-03 | 66,38 | 119,18 |
| RPL35A | -1,19 | 8,99E-03 | 820,27 | 1479,25 |
| TMEM258 | -1,18 | 6,12E-03 | 145,23 | 257,81 |
| ABHD10 | -1,18 | 5,09E-03 | 8,12 | 13,57 |
| C1orf194 | -1,18 | 2,43E-03 | 28,49 | 55,95 |
| AC093323.1 | -1,18 | 5,22E-03 | 3,19 | 5,73 |
| PRDX5 | -1,18 | 4,92E-03 | 276,52 | 485,95 |
| CD320 | -1,18 | 3,26E-03 | 6,46 | 11,88 |
| POLR2H | -1,18 | 1,28E-03 | 55,82 | 104,96 |
| C12orf57 | -1,17 | 3,02E-03 | 123,52 | 217,05 |
| DYNC2H1 | -1,17 | 3,56E-03 | 8,00 | 17,25 |
| CRISPLD1 | -1,17 | 9,87E-03 | 3,99 | 6,11 |
| STK33 | -1,17 | 3,49E-03 | 9,54 | 16,67 |
| MRPL17 | -1,16 | 2,79E-03 | 21,38 | 40,24 |
| MT-ND6 | -1,16 | 2,54E-03 | 135,47 | 244,12 |
| CHN1 | -1,15 | 4,56E-03 | 2,32 | 3,84 |
| NDUFS4 | -1,15 | 8,04E-03 | 39,64 | 68,91 |
| CCDC34 | -1,15 | 4,23E-03 | 2,04 | 4,44 |
| UQCRB | -1,15 | 8,98E-03 | 186,45 | 337,92 |
| KLHDC1 | -1,15 | 9,02E-03 | 1,25 | 2,01 |
| SOD1 | -1,15 | 6,21E-03 | 92,37 | 159,37 |
| NDUFA7 | -1,15 | 5,30E-03 | 41,52 | 70,65 |
| LINC01355 | -1,14 | 3,35E-03 | 1,82 | 3,31 |
| FABP6 | -1,14 | 7,56E-03 | 11,06 | 18,78 |
| COX14 | -1,14 | 4,06E-03 | 46,41 | 77,98 |
| PIGP | -1,13 | 1,53E-03 | 12,53 | 22,87 |
| FUNDC1 | -1,13 | 4,29E-03 | 9,24 | 16,61 |
| CRYZ | -1,13 | 6,85E-03 | 24,50 | 46,21 |
| EFHC1 | -1,13 | 2,87E-03 | 25,56 | 47,96 |
| AC018638.6 | -1,13 | 7,70E-03 | 1,28 | 2,37 |
| C12orf73 | -1,12 | 1,13E-03 | 3,76 | 6,25 |
| DPY30 | -1,12 | 5,21E-03 | 46,06 | 77,50 |
| AC004918.1 | -1,12 | 5,25E-04 | 4,54 | 8,15 |
| C17orf97 | -1,12 | 2,41E-03 | 3,20 | 6,22 |
| CHCHD1 | -1,12 | 3,26E-03 | 36,97 | 65,48 |
| ARSK | -1,12 | 7,24E-03 | 1,46 | 2,46 |
| MT-ND2 | -1,11 | 2,12E-03 | 7240,50 | 12729,98 |
| PPA2 | -1,11 | 5,25E-03 | 46,94 | 80,78 |
| IFT43 | -1,10 | 4,82E-03 | 23,55 | 39,23 |
| C6orf203 | -1,10 | 4,40E-03 | 11,27 | 19,16 |
| TMEM17 | -1,10 | 7,70E-03 | 0,97 | 1,67 |
| SPAG17 | -1,10 | 9,90E-03 | 19,91 | 43,08 |
| NEK3 | -1,10 | 1,98E-03 | 5,80 | 9,94 |
| LMAN2L | -1,10 | 5,58E-03 | 8,79 | 14,12 |
| ZDHHC4 | -1,09 | 6,29E-03 | 34,79 | 62,30 |
| PIN4 | -1,09 | 3,38E-03 | 11,45 | 19,43 |
| PIGF | -1,09 | 5,98E-03 | 16,12 | 33,10 |
| ARV1 | -1,08 | 7,99E-03 | 10,60 | 17,65 |
| ZGLP1 | -1,08 | 1,93E-03 | 4,50 | 7,87 |
| KLHDC2 | -1,08 | 9,61E-03 | 28,57 | 47,74 |
| ZNF248 | -1,08 | 4,23E-03 | 4,74 | 7,37 |
| TRIM16L | -1,08 | 5,71E-03 | 2,58 | 3,98 |
| ELP6 | -1,08 | 5,60E-03 | 13,03 | 20,57 |
| FAM104B | -1,08 | 5,94E-03 | 11,79 | 20,21 |
| CNPY2 | -1,08 | 4,35E-03 | 87,64 | 142,46 |
| ATP5IF1 | -1,08 | 3,79E-03 | 156,16 | 269,70 |
| HIBADH | -1,07 | 6,98E-03 | 14,67 | 24,60 |
| CISD1 | -1,07 | 9,87E-03 | 29,34 | 50,89 |
| TRIAP1 | -1,07 | 6,45E-03 | 14,60 | 24,29 |
| HSPE1 | -1,05 | 4,00E-03 | 90,63 | 147,90 |
| SMIM19 | -1,04 | 5,10E-03 | 24,84 | 43,14 |
| RP9 | -1,04 | 1,62E-04 | 7,09 | 12,32 |
| HSPB11 | -1,04 | 6,90E-03 | 22,58 | 36,13 |
| ALG8 | -1,04 | 8,99E-03 | 18,71 | 33,05 |
| THYN1 | -1,04 | 7,67E-03 | 24,15 | 38,56 |
| WDR19 | -1,04 | 3,97E-03 | 24,50 | 42,97 |
| TTC25 | -1,04 | 9,00E-03 | 8,24 | 13,03 |
| MRPL27 | -1,03 | 5,98E-03 | 47,50 | 80,89 |
| MRPL39 | -1,03 | 5,09E-03 | 11,71 | 20,01 |
| ALG5 | -1,03 | 9,63E-03 | 30,52 | 49,74 |
| ATRAID | -1,03 | 9,02E-03 | 83,85 | 135,96 |
| TMEM205 | -1,03 | 2,33E-05 | 114,53 | 196,02 |
| ZNF593 | -1,03 | 1,12E-03 | 4,86 | 8,24 |
| IFT46 | -1,03 | 3,91E-03 | 13,84 | 22,55 |
| ZNF799 | -1,02 | 4,28E-03 | 3,26 | 5,43 |
| ZNF547 | -1,02 | 2,90E-04 | 3,80 | 6,00 |
| FKBP3 | -1,02 | 5,02E-03 | 29,13 | 47,15 |
| NDUFAF8 | -1,01 | 9,41E-03 | 36,54 | 53,66 |
| MRPS17 | -1,00 | 1,99E-03 | 5,79 | 10,22 |
| HPRT1 | 1,00 | 1,58E-03 | 23,79 | 11,44 |
| FCHSD1 | 1,00 | 7,02E-03 | 13,08 | 4,74 |
| LGALS9 | 1,00 | 6,64E-03 | 138,43 | 54,55 |
| TANGO2 | 1,01 | 2,77E-03 | 17,89 | 7,77 |
| MIIP | 1,01 | 4,32E-03 | 17,10 | 8,68 |
| ZNF217 | 1,01 | 4,51E-03 | 17,88 | 8,10 |
| PRDM2 | 1,01 | 4,35E-03 | 18,17 | 8,39 |
| PKN1 | 1,01 | 2,54E-03 | 24,48 | 14,97 |
| LPAR5 | 1,02 | 9,60E-03 | 1,71 | 0,79 |
| CRKL | 1,03 | 2,83E-03 | 16,03 | 7,50 |
| GOLIM4 | 1,03 | 9,74E-03 | 6,13 | 2,49 |
| AKAP2 | 1,03 | 3,55E-03 | 8,92 | 3,70 |
| EWSR1 | 1,03 | 6,31E-03 | 135,87 | 66,52 |
| NAGA | 1,03 | 1,61E-04 | 30,09 | 13,23 |
| RNF169 | 1,04 | 8,77E-03 | 7,44 | 3,31 |
| SBF2 | 1,04 | 3,78E-03 | 23,40 | 12,24 |
| AVL9 | 1,04 | 1,20E-03 | 22,54 | 9,84 |
| ENO1 | 1,04 | 2,83E-03 | 781,26 | 341,22 |
| DNM2 | 1,04 | 6,02E-03 | 88,48 | 37,77 |
| VTI1A | 1,04 | 7,64E-03 | 11,74 | 4,91 |
| LIN37 | 1,05 | 8,54E-03 | 9,03 | 4,07 |
| ZBTB49 | 1,05 | 7,71E-03 | 3,64 | 1,45 |
| CTSC | 1,05 | 4,31E-04 | 146,73 | 66,61 |
| TOP3A | 1,06 | 4,70E-03 | 12,12 | 6,32 |
| CCM2 | 1,06 | 4,70E-03 | 27,04 | 11,61 |
| SIRT7 | 1,06 | 7,70E-03 | 23,43 | 10,58 |
| MED28 | 1,06 | 8,01E-03 | 57,38 | 15,43 |
| ZBTB2 | 1,06 | 4,32E-03 | 6,79 | 3,05 |
| ZBTB17 | 1,07 | 4,00E-03 | 22,73 | 12,64 |
| UTRN | 1,07 | 5,02E-03 | 17,61 | 7,10 |
| WDR81 | 1,07 | 9,38E-04 | 11,09 | 5,51 |
| MARS | 1,07 | 4,84E-03 | 82,82 | 48,61 |
| MGA | 1,07 | 5,51E-03 | 7,49 | 3,22 |
| NCOA3 | 1,07 | 2,90E-03 | 12,62 | 5,38 |
| CNTRL | 1,07 | 1,16E-03 | 9,93 | 4,18 |
| VAC14 | 1,07 | 3,60E-03 | 21,97 | 13,93 |
| STAT6 | 1,08 | 8,26E-03 | 129,05 | 55,33 |
| APLP2 | 1,08 | 5,24E-03 | 234,02 | 98,72 |
| TRAPPC10 | 1,08 | 3,90E-03 | 22,24 | 8,96 |
| SERINC5 | 1,08 | 1,50E-03 | 13,15 | 5,39 |
| LIN54 | 1,08 | 3,78E-03 | 5,33 | 1,84 |
| MAD1L1 | 1,08 | 5,09E-03 | 18,19 | 7,58 |
| ATP9B | 1,08 | 8,67E-03 | 13,21 | 6,85 |
| PIKFYVE | 1,09 | 1,43E-03 | 7,57 | 3,21 |
| PDK1 | 1,09 | 4,22E-03 | 11,63 | 5,06 |
| SUSD1 | 1,09 | 2,79E-03 | 9,73 | 4,99 |
| MAP3K14 | 1,09 | 3,26E-03 | 4,57 | 2,23 |
| DEF6 | 1,09 | 4,76E-03 | 31,22 | 12,58 |
| KIDINS220 | 1,09 | 6,89E-03 | 21,71 | 9,39 |
| CARS | 1,10 | 5,00E-03 | 35,34 | 16,36 |
| CDKN1B | 1,10 | 1,48E-03 | 22,00 | 9,49 |
| SEC23A | 1,10 | 3,53E-03 | 16,42 | 7,50 |
| TUBA1B | 1,10 | 8,41E-04 | 404,33 | 163,77 |
| TGOLN2 | 1,10 | 7,33E-04 | 70,99 | 30,52 |
| TNPO3 | 1,10 | 2,58E-03 | 14,60 | 6,30 |
| C6orf62 | 1,11 | 2,54E-03 | 98,75 | 48,67 |
| CBLL1 | 1,11 | 1,47E-03 | 11,81 | 3,86 |
| TAB3 | 1,11 | 4,10E-03 | 10,67 | 4,45 |
| IREB2 | 1,11 | 3,72E-03 | 15,88 | 5,63 |
| RAP2B | 1,12 | 1,97E-03 | 6,92 | 2,94 |
| MLX | 1,12 | 1,10E-03 | 40,54 | 16,33 |
| PARP11 | 1,12 | 1,64E-03 | 5,36 | 1,98 |
| GATAD2B | 1,12 | 8,44E-03 | 5,59 | 2,42 |
| WIPF2 | 1,12 | 5,78E-03 | 13,31 | 5,57 |
| WASHC2C | 1,12 | 8,07E-03 | 35,93 | 18,16 |
| TMBIM1 | 1,12 | 8,37E-03 | 137,09 | 59,02 |
| ZNF81 | 1,13 | 5,49E-03 | 2,79 | 1,24 |
| PLEKHA2 | 1,13 | 9,78E-03 | 27,70 | 10,73 |
| ZNF407 | 1,13 | 6,21E-03 | 2,62 | 1,35 |
| HERPUD2 | 1,13 | 2,41E-03 | 16,56 | 6,35 |
| SP140L | 1,13 | 4,27E-04 | 19,10 | 7,08 |
| MARCH2 | 1,13 | 2,64E-03 | 31,01 | 12,42 |
| ZSWIM8 | 1,13 | 7,59E-03 | 69,98 | 43,23 |
| NFYC | 1,14 | 3,80E-03 | 42,81 | 17,27 |
| MED1 | 1,14 | 1,49E-03 | 13,13 | 6,56 |
| GRIPAP1 | 1,14 | 2,61E-03 | 28,71 | 11,29 |
| ERICH1 | 1,14 | 6,04E-03 | 15,49 | 6,83 |
| NUSAP1 | 1,14 | 5,30E-03 | 5,16 | 2,61 |
| MCM5 | 1,14 | 3,52E-03 | 8,80 | 4,09 |
| CLIP2 | 1,14 | 6,02E-04 | 2,68 | 1,17 |
| ANKHD1 | 1,15 | 1,61E-03 | 78,35 | 30,75 |
| RAP2A | 1,15 | 5,14E-03 | 6,34 | 2,69 |
| PSD4 | 1,15 | 8,83E-03 | 22,74 | 10,71 |
| TMEM86A | 1,15 | 7,27E-04 | 3,70 | 1,49 |
| DIAPH1 | 1,15 | 4,00E-03 | 57,67 | 27,33 |
| FHOD1 | 1,16 | 2,44E-04 | 16,47 | 8,24 |
| SMIM29 | 1,16 | 9,44E-03 | 24,14 | 10,12 |
| MYLIP | 1,16 | 2,86E-03 | 39,10 | 15,20 |
| SPNS1 | 1,17 | 5,09E-03 | 16,35 | 8,18 |
| TECPR1 | 1,17 | 6,76E-04 | 14,19 | 4,20 |
| ASH1L | 1,17 | 8,55E-03 | 10,99 | 4,74 |
| PPM1M | 1,17 | 1,99E-03 | 31,73 | 11,85 |
| SREBF1 | 1,17 | 6,20E-03 | 44,36 | 15,13 |
| GBA | 1,17 | 7,96E-03 | 56,74 | 31,92 |
| KIAA0513 | 1,18 | 6,56E-03 | 15,10 | 5,55 |
| STARD3 | 1,18 | 1,03E-03 | 40,80 | 14,25 |
| RINL | 1,19 | 8,97E-03 | 9,70 | 2,59 |
| NCSTN | 1,19 | 7,61E-03 | 86,14 | 35,66 |
| SLC25A13 | 1,19 | 9,79E-03 | 26,95 | 8,48 |
| ZNF687 | 1,19 | 3,01E-03 | 10,09 | 4,17 |
| ASXL2 | 1,19 | 3,61E-04 | 7,84 | 3,16 |
| CTSA | 1,19 | 1,52E-03 | 102,01 | 40,34 |
| OTULIN | 1,19 | 3,79E-03 | 10,23 | 3,91 |
| SBNO1 | 1,20 | 8,24E-03 | 14,71 | 5,48 |
| GTF2IP4 | 1,20 | 5,84E-05 | 20,68 | 7,73 |
| PEAK1 | 1,20 | 2,34E-03 | 4,50 | 1,93 |
| SLC23A2 | 1,20 | 2,95E-03 | 6,89 | 2,59 |
| TMEM127 | 1,20 | 4,20E-03 | 47,92 | 16,56 |
| PLIN3 | 1,20 | 7,71E-03 | 58,24 | 24,73 |
| INPP4A | 1,20 | 3,92E-03 | 7,86 | 2,76 |
| RNF44 | 1,20 | 8,77E-03 | 20,17 | 7,61 |
| BCKDK | 1,21 | 2,74E-03 | 30,55 | 13,20 |
| CCDC18 | 1,21 | 2,98E-03 | 3,72 | 1,39 |
| ZNF106 | 1,21 | 1,80E-03 | 18,06 | 6,55 |
| ADA2 | 1,21 | 9,55E-03 | 34,56 | 14,10 |
| GPAT4 | 1,21 | 5,10E-03 | 109,45 | 34,18 |
| GGA3 | 1,22 | 3,85E-04 | 25,73 | 8,81 |
| VAMP1 | 1,22 | 1,14E-05 | 4,89 | 2,41 |
| FAM219A | 1,22 | 3,90E-03 | 2,95 | 1,21 |
| MGAT4A | 1,22 | 8,01E-03 | 27,23 | 10,45 |
| SLC29A3 | 1,22 | 5,25E-03 | 10,11 | 3,97 |
| RAB11FIP1 | 1,22 | 9,07E-03 | 40,81 | 15,48 |
| CDK11A | 1,22 | 7,22E-04 | 35,27 | 13,95 |
| TEP1 | 1,22 | 6,19E-04 | 25,79 | 10,90 |
| COLGALT1 | 1,22 | 5,48E-05 | 40,95 | 16,54 |
| NAPA | 1,23 | 7,26E-03 | 92,09 | 40,71 |
| PML | 1,23 | 9,41E-03 | 31,57 | 13,56 |
| CCSAP | 1,23 | 7,94E-03 | 3,30 | 1,28 |
| SNX27 | 1,23 | 3,51E-03 | 12,54 | 4,58 |
| NUFIP2 | 1,24 | 2,02E-03 | 21,99 | 8,62 |
| VPS13B | 1,24 | 5,50E-03 | 9,53 | 4,66 |
| PNPLA6 | 1,24 | 1,24E-03 | 28,69 | 15,22 |
| RGL1 | 1,24 | 2,26E-05 | 14,74 | 5,65 |
| PARP10 | 1,24 | 2,26E-03 | 41,68 | 12,93 |
| KIF2C | 1,24 | 1,49E-03 | 1,89 | 0,59 |
| TMEM43 | 1,24 | 6,43E-03 | 35,96 | 14,74 |
| DCLRE1B | 1,25 | 1,81E-03 | 3,75 | 1,47 |
| ARL4C | 1,25 | 2,67E-03 | 13,84 | 5,54 |
| SMG1P2 | 1,26 | 6,25E-03 | 4,43 | 1,29 |
| WDFY1 | 1,26 | 7,81E-03 | 31,73 | 9,39 |
| FRAT1 | 1,26 | 7,22E-03 | 7,45 | 2,93 |
| SEL1L | 1,26 | 2,68E-03 | 32,31 | 11,49 |
| IFI35 | 1,26 | 6,44E-03 | 36,82 | 13,31 |
| POLR2A | 1,26 | 9,03E-03 | 28,99 | 10,47 |
| CTSB | 1,26 | 2,27E-03 | 2593,20 | 852,12 |
| PRR14L | 1,27 | 1,57E-03 | 11,25 | 5,27 |
| FUT4 | 1,27 | 5,02E-03 | 4,70 | 1,91 |
| ABAT | 1,27 | 2,96E-04 | 5,44 | 1,94 |
| AC125232.1 | 1,27 | 4,01E-03 | 5,93 | 2,22 |
| AC099343.3 | 1,27 | 7,70E-03 | 2,81 | 1,08 |
| ACER3 | 1,27 | 2,71E-03 | 13,82 | 4,58 |
| CNEP1R1 | 1,27 | 6,91E-04 | 23,91 | 8,69 |
| SULF2 | 1,27 | 1,95E-03 | 66,10 | 27,49 |
| LPAR2 | 1,27 | 8,08E-03 | 54,98 | 16,70 |
| HERC1 | 1,27 | 7,77E-04 | 38,17 | 17,45 |
| KPNA4 | 1,27 | 3,19E-03 | 20,97 | 7,10 |
| USP3 | 1,27 | 4,88E-03 | 48,22 | 18,52 |
| MEF2D | 1,27 | 6,90E-03 | 17,49 | 5,70 |
| ME2 | 1,27 | 7,64E-03 | 27,29 | 11,85 |
| ITSN2 | 1,28 | 3,26E-03 | 39,26 | 15,62 |
| SPEN | 1,28 | 1,78E-03 | 18,04 | 7,65 |
| GNA12 | 1,28 | 2,86E-03 | 12,75 | 4,83 |
| HK2 | 1,28 | 5,55E-03 | 39,03 | 15,97 |
| SFXN3 | 1,28 | 2,09E-04 | 25,08 | 9,11 |
| DTNBP1 | 1,28 | 8,50E-03 | 18,75 | 7,12 |
| NR1H3 | 1,28 | 6,44E-03 | 44,30 | 20,29 |
| IKBKB | 1,28 | 9,61E-04 | 39,47 | 17,32 |
| PIP4K2A | 1,28 | 6,98E-05 | 21,99 | 6,57 |
| KDM2A | 1,29 | 3,24E-03 | 45,90 | 17,03 |
| NECAP2 | 1,29 | 5,28E-03 | 57,95 | 21,92 |
| MED15 | 1,29 | 7,20E-03 | 36,59 | 14,63 |
| PRKCA | 1,29 | 5,10E-03 | 2,27 | 0,78 |
| DENND1A | 1,29 | 8,45E-03 | 9,83 | 2,91 |
| DICER1 | 1,29 | 6,02E-03 | 25,44 | 10,51 |
| CLCN6 | 1,29 | 2,19E-03 | 8,07 | 2,67 |
| PI4K2A | 1,29 | 8,39E-04 | 12,10 | 4,76 |
| TNFRSF10A | 1,29 | 3,61E-03 | 12,04 | 4,74 |
| LIMK1 | 1,29 | 3,03E-03 | 12,85 | 4,87 |
| GNB1 | 1,29 | 4,81E-03 | 196,87 | 62,45 |
| EIF3A | 1,29 | 4,15E-03 | 48,16 | 19,34 |
| MVP | 1,30 | 6,31E-03 | 165,40 | 56,87 |
| TMEM170B | 1,30 | 7,56E-03 | 2,40 | 0,77 |
| NDST2 | 1,30 | 2,11E-03 | 17,78 | 6,85 |
| TRIM22 | 1,30 | 8,43E-03 | 130,10 | 48,29 |
| SP2 | 1,30 | 3,56E-03 | 9,73 | 3,72 |
| VPS26C | 1,30 | 1,03E-04 | 35,54 | 13,96 |
| GRK2 | 1,30 | 7,34E-03 | 62,05 | 22,49 |
| ZBTB34 | 1,30 | 6,40E-04 | 6,92 | 2,57 |
| TFIP11 | 1,31 | 2,54E-03 | 27,77 | 12,59 |
| DPYSL2 | 1,31 | 3,91E-05 | 7,35 | 2,66 |
| KDM5A | 1,31 | 4,17E-03 | 20,93 | 6,75 |
| ZBTB37 | 1,31 | 4,87E-03 | 3,07 | 1,23 |
| ARL8A | 1,31 | 4,83E-03 | 36,19 | 14,49 |
| GLUL | 1,31 | 7,16E-03 | 1761,08 | 481,74 |
| C1QA | 1,31 | 7,58E-03 | 78,79 | 27,20 |
| EHD4 | 1,31 | 1,08E-03 | 20,22 | 7,64 |
| AC009506.1 | 1,32 | 2,49E-03 | 8,87 | 3,28 |
| PHF20 | 1,32 | 6,16E-04 | 15,76 | 4,63 |
| CAPZB | 1,32 | 3,26E-03 | 171,73 | 61,89 |
| P2RX4 | 1,32 | 5,87E-03 | 55,57 | 23,27 |
| ADAM17 | 1,33 | 9,50E-03 | 26,31 | 10,27 |
| AC087190.3 | 1,33 | 3,61E-03 | 14,67 | 5,36 |
| ATP6AP1 | 1,33 | 5,85E-03 | 118,18 | 46,30 |
| RNF166 | 1,33 | 5,48E-03 | 26,41 | 8,99 |
| ACRBP | 1,33 | 5,68E-03 | 3,58 | 1,26 |
| UBQLN2 | 1,33 | 3,03E-03 | 15,63 | 6,03 |
| VPS4B | 1,34 | 3,40E-03 | 40,56 | 13,51 |
| GMEB1 | 1,34 | 3,83E-03 | 8,17 | 2,22 |
| LTA4H | 1,34 | 3,26E-03 | 151,04 | 57,88 |
| ARHGEF1 | 1,34 | 2,35E-03 | 83,84 | 33,54 |
| SH3GLB1 | 1,34 | 9,27E-03 | 125,17 | 38,72 |
| FNBP1 | 1,34 | 9,86E-03 | 39,25 | 13,80 |
| ATF7IP | 1,35 | 2,28E-03 | 12,92 | 4,77 |
| GINS4 | 1,35 | 4,13E-03 | 1,60 | 0,70 |
| ZNF319 | 1,35 | 3,24E-03 | 7,31 | 2,92 |
| WDR1 | 1,35 | 7,70E-03 | 171,77 | 56,98 |
| ATP7A | 1,35 | 9,55E-03 | 4,79 | 1,91 |
| RBMS1 | 1,35 | 6,86E-03 | 49,60 | 16,31 |
| STAT2 | 1,35 | 1,54E-03 | 114,34 | 36,99 |
| PARP15 | 1,35 | 4,51E-03 | 5,65 | 2,26 |
| LRP1 | 1,36 | 8,27E-04 | 15,40 | 6,86 |
| DEDD2 | 1,36 | 8,47E-03 | 53,58 | 18,77 |
| SGSH | 1,36 | 4,71E-04 | 19,60 | 8,62 |
| ERV3-1 | 1,36 | 1,54E-03 | 34,35 | 13,05 |
| GNPDA1 | 1,36 | 1,65E-04 | 33,34 | 11,32 |
| MTPN | 1,36 | 8,97E-03 | 68,49 | 24,35 |
| PALD1 | 1,36 | 1,57E-03 | 2,26 | 0,81 |
| RAF1 | 1,36 | 6,06E-03 | 88,98 | 30,48 |
| TMEM206 | 1,36 | 1,71E-04 | 6,17 | 2,12 |
| DHX8 | 1,36 | 2,19E-03 | 25,53 | 13,51 |
| C1RL-AS1 | 1,37 | 2,19E-03 | 4,63 | 1,78 |
| TPD52L2 | 1,37 | 3,51E-03 | 119,51 | 42,43 |
| MAP4K2 | 1,38 | 1,57E-03 | 8,78 | 3,46 |
| NEMP1 | 1,38 | 4,63E-03 | 6,75 | 2,50 |
| GNA15 | 1,38 | 8,66E-03 | 35,04 | 13,26 |
| DBNL | 1,38 | 5,36E-04 | 99,60 | 35,39 |
| NCOA2 | 1,38 | 5,27E-03 | 12,23 | 4,19 |
| FOXN3 | 1,38 | 2,41E-03 | 17,30 | 5,19 |
| MSL3 | 1,38 | 5,92E-03 | 48,68 | 15,34 |
| CFL1 | 1,38 | 4,92E-03 | 644,90 | 218,09 |
| BCL2L11 | 1,38 | 7,60E-03 | 20,11 | 7,38 |
| BICRAL | 1,38 | 2,88E-04 | 7,57 | 2,77 |
| BTN3A1 | 1,39 | 9,98E-04 | 51,23 | 16,65 |
| SLC25A43 | 1,39 | 1,06E-03 | 4,30 | 1,60 |
| MARK2 | 1,39 | 7,99E-03 | 20,87 | 6,87 |
| MFSD13A | 1,39 | 5,55E-04 | 3,50 | 1,17 |
| SNRK | 1,39 | 5,61E-03 | 17,50 | 6,24 |
| PSMB8 | 1,39 | 7,12E-03 | 147,85 | 52,79 |
| VPS37C | 1,39 | 1,76E-03 | 25,93 | 7,44 |
| HNRNPF | 1,39 | 4,20E-03 | 174,41 | 58,50 |
| ZZEF1 | 1,39 | 4,95E-03 | 13,65 | 4,72 |
| APBA3 | 1,39 | 3,19E-03 | 20,95 | 7,36 |
| SOAT1 | 1,40 | 1,17E-03 | 48,13 | 12,47 |
| PLXNA1 | 1,40 | 7,64E-03 | 12,68 | 4,44 |
| ABI1 | 1,40 | 2,34E-03 | 46,81 | 15,65 |
| EML4 | 1,40 | 1,12E-03 | 57,89 | 20,74 |
| RNF111 | 1,41 | 9,68E-04 | 15,35 | 5,86 |
| LPIN2 | 1,41 | 1,52E-03 | 24,88 | 7,63 |
| FMNL2 | 1,41 | 4,50E-03 | 16,04 | 6,11 |
| LRCH4 | 1,41 | 3,64E-03 | 65,84 | 21,33 |
| MAPKAPK3 | 1,41 | 7,71E-04 | 36,52 | 12,26 |
| TYK2 | 1,41 | 7,27E-04 | 119,01 | 60,19 |
| FBXO6 | 1,41 | 3,71E-03 | 19,94 | 6,85 |
| ATP6V1A | 1,42 | 3,57E-03 | 55,31 | 18,74 |
| GPATCH2L | 1,42 | 2,02E-03 | 16,35 | 4,21 |
| STXBP2 | 1,42 | 3,02E-03 | 130,91 | 42,80 |
| CEP135 | 1,42 | 5,83E-03 | 5,26 | 1,87 |
| TMOD3 | 1,42 | 1,21E-03 | 26,36 | 9,71 |
| RTN1 | 1,42 | 8,72E-03 | 10,27 | 4,25 |
| RAB35 | 1,42 | 3,50E-03 | 37,38 | 13,21 |
| CDK14 | 1,42 | 7,79E-03 | 8,50 | 2,55 |
| DOPEY2 | 1,42 | 3,20E-08 | 6,26 | 1,88 |
| FOLR2 | 1,42 | 2,05E-03 | 6,34 | 2,14 |
| TNFRSF14 | 1,43 | 3,64E-03 | 193,77 | 73,85 |
| MAP1S | 1,43 | 3,02E-03 | 15,23 | 7,97 |
| TMEM2 | 1,43 | 5,57E-03 | 23,15 | 7,94 |
| MRAS | 1,43 | 2,95E-03 | 6,82 | 2,21 |
| SLC3A2 | 1,43 | 2,41E-03 | 108,48 | 37,60 |
| VHL | 1,43 | 3,79E-03 | 27,52 | 9,72 |
| PHC1P1 | 1,43 | 4,75E-03 | 3,24 | 1,28 |
| WWP2 | 1,43 | 2,39E-03 | 28,00 | 9,64 |
| MAP4K1 | 1,43 | 6,03E-03 | 5,85 | 2,70 |
| DCK | 1,44 | 1,70E-03 | 8,82 | 3,07 |
| KBTBD2 | 1,44 | 9,61E-03 | 39,86 | 12,25 |
| PCBP1 | 1,44 | 8,65E-03 | 298,45 | 101,95 |
| PLEKHM1 | 1,45 | 4,55E-03 | 21,42 | 7,44 |
| PDGFB | 1,45 | 7,76E-04 | 3,64 | 1,27 |
| RPS6KA3 | 1,45 | 5,24E-03 | 27,39 | 8,36 |
| PGD | 1,45 | 2,05E-03 | 147,67 | 49,03 |
| SPATA13 | 1,45 | 2,85E-03 | 29,02 | 9,55 |
| ACOT9 | 1,45 | 2,85E-03 | 64,66 | 22,03 |
| NFATC1 | 1,45 | 4,56E-03 | 5,44 | 1,91 |
| MAML1 | 1,45 | 2,89E-03 | 12,95 | 4,59 |
| TBC1D2B | 1,46 | 2,65E-04 | 21,42 | 7,39 |
| C5orf56 | 1,46 | 1,34E-03 | 22,86 | 8,17 |
| MTMR10 | 1,46 | 1,66E-05 | 16,91 | 5,53 |
| MNT | 1,46 | 5,11E-04 | 14,50 | 4,36 |
| PXK | 1,46 | 6,67E-04 | 19,49 | 6,38 |
| USP49 | 1,46 | 2,89E-03 | 3,13 | 1,00 |
| LIPA | 1,46 | 8,20E-03 | 88,63 | 27,00 |
| ARHGAP27 | 1,46 | 5,21E-03 | 65,93 | 19,20 |
| AL049840.3 | 1,47 | 4,02E-03 | 8,60 | 2,91 |
| HHEX | 1,47 | 1,12E-03 | 2,92 | 0,87 |
| TPM3 | 1,47 | 4,62E-03 | 385,52 | 111,12 |
| S100B | 1,47 | 5,66E-05 | 14,11 | 4,17 |
| MSL2 | 1,47 | 7,94E-04 | 19,52 | 5,60 |
| XIAP | 1,47 | 5,22E-03 | 42,49 | 14,86 |
| YWHAG | 1,47 | 7,83E-04 | 38,49 | 13,60 |
| CAMKK1 | 1,47 | 1,08E-03 | 6,32 | 2,19 |
| ZFYVE1 | 1,47 | 1,36E-04 | 10,70 | 3,56 |
| RAB5C | 1,47 | 6,23E-03 | 167,58 | 55,10 |
| CD6 | 1,48 | 2,17E-03 | 6,24 | 1,95 |
| DIAPH2 | 1,48 | 1,40E-03 | 3,59 | 1,15 |
| LASP1 | 1,48 | 2,45E-03 | 88,86 | 30,27 |
| NUB1 | 1,48 | 4,70E-03 | 85,72 | 23,27 |
| YPEL3 | 1,48 | 7,04E-03 | 157,91 | 57,02 |
| MAF | 1,48 | 6,55E-04 | 5,46 | 1,71 |
| PFKFB4 | 1,48 | 6,27E-03 | 11,90 | 3,79 |
| JUN | 1,48 | 7,22E-04 | 22,23 | 6,99 |
| MCUB | 1,48 | 4,61E-03 | 10,28 | 3,42 |
| CXorf38 | 1,48 | 2,50E-05 | 14,16 | 5,25 |
| TBL1X | 1,48 | 4,47E-03 | 15,52 | 5,23 |
| FAM214B | 1,49 | 3,68E-03 | 24,74 | 8,03 |
| GRK5 | 1,49 | 5,44E-03 | 3,90 | 1,27 |
| FBRS | 1,49 | 8,35E-03 | 65,26 | 22,11 |
| TTL | 1,49 | 6,95E-03 | 12,78 | 3,93 |
| TBC1D14 | 1,49 | 1,49E-03 | 41,41 | 14,14 |
| IL13RA1 | 1,49 | 5,83E-03 | 68,88 | 21,68 |
| USF3 | 1,50 | 1,76E-03 | 9,55 | 4,65 |
| EIF4HP1 | 1,50 | 8,66E-03 | 6,96 | 2,21 |
| BBC3 | 1,50 | 3,65E-03 | 12,45 | 4,01 |
| ATP6V0D1 | 1,50 | 4,90E-03 | 125,49 | 40,59 |
| HPCAL1 | 1,50 | 2,93E-03 | 23,69 | 8,25 |
| RASSF4 | 1,50 | 6,35E-05 | 42,88 | 15,36 |
| sept.06 | 1,50 | 1,87E-05 | 20,56 | 5,90 |
| OPLAH | 1,50 | 8,90E-05 | 6,16 | 1,76 |
| TIMP2 | 1,51 | 7,83E-04 | 33,74 | 11,16 |
| SLC25A19 | 1,51 | 1,09E-03 | 15,90 | 5,29 |
| SCAF11 | 1,51 | 5,57E-04 | 64,17 | 26,00 |
| SPRED1 | 1,51 | 9,95E-04 | 8,68 | 2,14 |
| IDS | 1,51 | 4,94E-03 | 82,70 | 24,48 |
| CTC1 | 1,51 | 6,46E-04 | 23,83 | 9,46 |
| TMEM268 | 1,51 | 2,16E-03 | 11,45 | 3,14 |
| ARID3B | 1,52 | 3,36E-04 | 6,25 | 1,91 |
| FCMR | 1,52 | 2,80E-03 | 8,64 | 2,61 |
| BAZ2A | 1,52 | 1,49E-03 | 39,36 | 10,33 |
| IRF9 | 1,52 | 3,33E-03 | 209,43 | 76,19 |
| ZAP70 | 1,52 | 5,91E-03 | 5,60 | 2,16 |
| SAMD8 | 1,52 | 4,20E-03 | 13,36 | 4,04 |
| TKT | 1,53 | 7,34E-03 | 116,55 | 38,38 |
| IMPDH1 | 1,53 | 5,94E-03 | 64,99 | 20,28 |
| FHDC1 | 1,53 | 6,70E-03 | 3,70 | 1,25 |
| CDCA5 | 1,53 | 3,59E-03 | 1,78 | 0,52 |
| TOP2A | 1,53 | 9,25E-05 | 5,00 | 2,22 |
| WNK1 | 1,53 | 1,12E-03 | 33,17 | 11,81 |
| WSB2 | 1,53 | 2,97E-04 | 42,83 | 14,80 |
| ZFYVE26 | 1,54 | 1,97E-04 | 9,74 | 3,00 |
| APOL2 | 1,54 | 1,86E-04 | 104,10 | 27,12 |
| OGFR | 1,54 | 3,85E-03 | 39,94 | 12,72 |
| AKIRIN2 | 1,54 | 2,88E-03 | 46,34 | 14,44 |
| PCBP1-AS1 | 1,54 | 8,24E-03 | 38,34 | 12,62 |
| PCYT1A | 1,54 | 7,71E-04 | 46,19 | 13,12 |
| FLOT1 | 1,55 | 8,77E-03 | 365,49 | 109,29 |
| CYB561A3 | 1,55 | 6,90E-03 | 46,85 | 16,77 |
| MOB1A | 1,55 | 3,42E-03 | 114,67 | 29,79 |
| CSK | 1,55 | 1,30E-03 | 59,48 | 18,09 |
| SNX9 | 1,55 | 8,74E-03 | 30,37 | 10,37 |
| ZNF641 | 1,55 | 5,88E-04 | 29,88 | 7,96 |
| SNAI3 | 1,55 | 5,92E-03 | 1,82 | 0,59 |
| C1orf162 | 1,55 | 3,05E-03 | 32,45 | 9,11 |
| ABR | 1,55 | 5,17E-04 | 53,59 | 17,48 |
| FGD2 | 1,55 | 2,68E-03 | 10,37 | 3,01 |
| KMT2E | 1,55 | 1,29E-03 | 34,94 | 10,78 |
| GNL3L | 1,56 | 9,20E-04 | 5,94 | 1,93 |
| TFEB | 1,56 | 5,53E-03 | 29,44 | 9,86 |
| NIN | 1,56 | 4,42E-03 | 24,99 | 8,57 |
| SMG1 | 1,56 | 5,45E-04 | 26,17 | 8,73 |
| IFNAR2 | 1,56 | 8,10E-03 | 38,06 | 10,97 |
| KMT2B | 1,56 | 2,27E-03 | 3,51 | 1,15 |
| CCND2 | 1,56 | 2,34E-03 | 4,83 | 1,62 |
| CTSZ | 1,57 | 1,11E-04 | 350,71 | 106,12 |
| RAB29 | 1,57 | 2,86E-03 | 17,25 | 5,14 |
| GRK3 | 1,57 | 1,04E-05 | 8,78 | 2,49 |
| MR1 | 1,57 | 5,26E-04 | 43,07 | 11,71 |
| TAOK3 | 1,57 | 4,84E-03 | 28,19 | 8,13 |
| XRN2 | 1,57 | 1,70E-03 | 60,76 | 19,79 |
| ZHX2 | 1,57 | 7,19E-03 | 10,81 | 3,54 |
| HMOX1 | 1,57 | 3,67E-03 | 50,79 | 14,80 |
| MAPK14 | 1,57 | 1,97E-03 | 52,37 | 14,53 |
| AC012085.1 | 1,57 | 1,40E-03 | 10,13 | 4,23 |
| CARD6 | 1,57 | 9,08E-04 | 7,69 | 2,46 |
| ATF3 | 1,58 | 7,64E-03 | 21,58 | 6,86 |
| CSGALNACT1 | 1,58 | 4,44E-03 | 29,18 | 8,51 |
| KMT2D | 1,58 | 1,97E-03 | 26,50 | 8,33 |
| ZSWIM4 | 1,58 | 7,76E-03 | 6,85 | 2,26 |
| UBR4 | 1,58 | 1,69E-03 | 76,41 | 38,00 |
| UBE2L6 | 1,59 | 5,65E-03 | 160,69 | 49,75 |
| CDC42SE1 | 1,59 | 8,87E-03 | 196,21 | 57,07 |
| KRAS | 1,59 | 2,34E-03 | 21,41 | 6,17 |
| PAPSS2 | 1,59 | 7,61E-03 | 11,27 | 3,76 |
| TCIRG1 | 1,59 | 5,99E-04 | 201,22 | 58,76 |
| ZCCHC2 | 1,59 | 1,31E-03 | 13,44 | 4,40 |
| WWC3 | 1,59 | 5,50E-03 | 12,09 | 3,62 |
| MLLT1 | 1,59 | 5,48E-03 | 10,91 | 3,62 |
| KMT2C | 1,59 | 1,40E-03 | 22,34 | 8,05 |
| GIT2 | 1,60 | 2,56E-04 | 36,12 | 9,55 |
| SGK3 | 1,60 | 3,26E-03 | 16,59 | 5,14 |
| PARP12 | 1,60 | 1,83E-04 | 40,38 | 11,10 |
| ZNF333 | 1,60 | 2,04E-04 | 13,45 | 3,50 |
| KCNN1 | 1,60 | 5,01E-03 | 2,36 | 0,28 |
| GRN | 1,60 | 4,80E-05 | 330,35 | 131,96 |
| AGTRAP | 1,60 | 1,53E-03 | 116,54 | 36,77 |
| PTPN1 | 1,60 | 2,01E-03 | 30,72 | 9,97 |
| UVRAG | 1,60 | 5,26E-04 | 10,72 | 2,97 |
| ABCD1 | 1,60 | 6,79E-06 | 4,87 | 1,65 |
| PACSIN2 | 1,60 | 2,05E-03 | 73,76 | 21,07 |
| NFKBIB | 1,61 | 7,41E-03 | 24,97 | 7,26 |
| RGS16 | 1,61 | 5,55E-03 | 3,53 | 1,14 |
| CHD2 | 1,61 | 4,00E-03 | 59,06 | 17,03 |
| NXPE3 | 1,61 | 7,39E-04 | 4,39 | 1,05 |
| ADPGK | 1,62 | 5,48E-05 | 93,48 | 30,07 |
| OSBPL11 | 1,62 | 1,62E-03 | 14,90 | 4,24 |
| ELMSAN1 | 1,62 | 4,96E-05 | 12,61 | 3,77 |
| PARP9 | 1,62 | 6,25E-03 | 97,70 | 33,79 |
| EDEM1 | 1,62 | 3,58E-04 | 25,59 | 7,98 |
| RXRA | 1,63 | 4,82E-03 | 44,86 | 11,98 |
| ARHGEF11 | 1,63 | 2,07E-04 | 23,62 | 5,70 |
| SIK3 | 1,63 | 2,60E-03 | 23,38 | 7,06 |
| BTG1 | 1,63 | 7,83E-03 | 129,52 | 39,31 |
| AL662844.4 | 1,63 | 7,33E-04 | 2,33 | 0,68 |
| GM2A | 1,63 | 2,78E-03 | 65,89 | 20,16 |
| TGFBR1 | 1,64 | 2,40E-05 | 24,68 | 6,39 |
| SH3BGRL3 | 1,64 | 1,41E-03 | 211,57 | 56,87 |
| CD7 | 1,64 | 8,29E-03 | 7,12 | 2,25 |
| PLEKHM2 | 1,64 | 1,26E-03 | 31,39 | 9,74 |
| HSD3B7 | 1,64 | 9,03E-09 | 14,40 | 4,06 |
| DLL1 | 1,64 | 5,26E-03 | 3,16 | 1,01 |
| EXOC8 | 1,65 | 4,13E-05 | 8,82 | 2,67 |
| ARHGAP10 | 1,65 | 2,28E-03 | 5,56 | 1,85 |
| ARAP2 | 1,65 | 4,62E-03 | 18,13 | 6,39 |
| MAP7D1 | 1,65 | 5,11E-04 | 50,64 | 15,76 |
| NLN | 1,65 | 5,48E-05 | 14,01 | 2,83 |
| TESK2 | 1,66 | 4,81E-03 | 5,92 | 1,87 |
| NCEH1 | 1,66 | 2,08E-04 | 10,00 | 2,89 |
| CARMIL2 | 1,66 | 2,01E-03 | 1,82 | 0,65 |
| TRERF1 | 1,66 | 3,50E-03 | 3,42 | 0,97 |
| PPARD | 1,66 | 1,54E-03 | 24,06 | 7,38 |
| AC110079.1 | 1,66 | 5,35E-03 | 5,45 | 1,67 |
| SLC25A40 | 1,66 | 3,79E-03 | 20,82 | 5,16 |
| CYTH1 | 1,66 | 6,48E-05 | 85,93 | 25,16 |
| G6PD | 1,66 | 3,39E-03 | 24,21 | 6,95 |
| CXCR6 | 1,66 | 6,61E-03 | 12,17 | 3,34 |
| CD1E | 1,66 | 2,01E-03 | 5,79 | 1,74 |
| LSS | 1,67 | 8,48E-03 | 24,15 | 7,87 |
| FAM102B | 1,67 | 9,91E-04 | 7,27 | 2,19 |
| SH3D21 | 1,67 | 9,67E-03 | 9,18 | 2,27 |
| BRI3 | 1,67 | 5,36E-03 | 294,22 | 85,60 |
| SUN2 | 1,67 | 1,50E-04 | 85,79 | 21,66 |
| FES | 1,68 | 8,99E-03 | 24,17 | 5,75 |
| RAPGEF6 | 1,68 | 1,66E-03 | 9,31 | 2,54 |
| FLOT2 | 1,68 | 7,03E-03 | 159,44 | 57,71 |
| TRIM38 | 1,68 | 2,53E-03 | 38,68 | 10,97 |
| AGO4 | 1,68 | 4,14E-04 | 29,93 | 9,86 |
| ANKH | 1,68 | 8,70E-09 | 12,63 | 3,04 |
| UPK3B | 1,68 | 3,50E-03 | 2,92 | 0,93 |
| ATXN1 | 1,68 | 1,64E-03 | 32,58 | 8,47 |
| PPP1R9B | 1,68 | 8,39E-04 | 24,59 | 7,56 |
| TSC22D4 | 1,68 | 1,72E-03 | 53,93 | 16,15 |
| NFAT5 | 1,68 | 6,32E-04 | 22,98 | 7,03 |
| MANBA | 1,68 | 2,92E-05 | 27,37 | 7,13 |
| TBC1D2 | 1,69 | 1,10E-04 | 39,93 | 12,31 |
| LAP3 | 1,69 | 3,54E-03 | 104,49 | 29,16 |
| AMDHD2 | 1,69 | 4,50E-04 | 29,67 | 7,11 |
| KIFC3 | 1,69 | 6,02E-04 | 70,28 | 17,11 |
| C1QB | 1,69 | 5,39E-04 | 111,00 | 29,28 |
| CHFR | 1,69 | 1,12E-04 | 29,71 | 9,49 |
| SQSTM1 | 1,69 | 7,46E-03 | 477,36 | 139,03 |
| CMTM6 | 1,70 | 8,37E-03 | 158,25 | 44,11 |
| METRNL | 1,70 | 6,41E-03 | 116,68 | 30,92 |
| SLCO4A1 | 1,70 | 8,98E-03 | 14,41 | 4,94 |
| PXN | 1,70 | 3,15E-03 | 95,72 | 26,74 |
| NUP62 | 1,70 | 1,62E-03 | 40,91 | 11,76 |
| SQOR | 1,70 | 3,79E-03 | 118,00 | 33,79 |
| MELK | 1,71 | 1,36E-04 | 1,56 | 0,45 |
| MTMR3 | 1,71 | 3,46E-04 | 36,03 | 9,37 |
| SP4 | 1,71 | 1,20E-03 | 1,97 | 0,66 |
| FHL3 | 1,71 | 1,12E-03 | 7,97 | 2,15 |
| PRR11 | 1,71 | 3,78E-04 | 1,74 | 0,50 |
| ATG16L2 | 1,71 | 4,10E-03 | 117,26 | 32,10 |
| RNASEL | 1,71 | 8,77E-04 | 17,07 | 4,68 |
| SH3BP1 | 1,72 | 4,65E-06 | 13,88 | 3,67 |
| BUB1 | 1,72 | 2,66E-05 | 1,74 | 0,38 |
| GNAI2 | 1,72 | 5,27E-03 | 222,15 | 62,37 |
| VCPIP1 | 1,72 | 2,69E-04 | 7,83 | 2,20 |
| AL512306.2 | 1,72 | 7,65E-03 | 1,78 | 0,49 |
| SRC | 1,73 | 2,71E-03 | 28,91 | 8,38 |
| ALDOC | 1,73 | 1,07E-05 | 14,81 | 4,37 |
| PATL1 | 1,73 | 1,61E-03 | 37,10 | 10,77 |
| UBE2C | 1,73 | 1,98E-03 | 4,83 | 1,51 |
| RBPJ | 1,74 | 1,50E-03 | 77,54 | 24,96 |
| SLC22A15 | 1,74 | 2,48E-03 | 4,46 | 1,16 |
| ZFAND3 | 1,74 | 3,34E-03 | 80,47 | 18,37 |
| NOTCH2 | 1,74 | 1,61E-04 | 40,50 | 11,41 |
| DISC1 | 1,74 | 1,14E-03 | 3,23 | 0,88 |
| LFNG | 1,74 | 4,66E-04 | 20,03 | 5,41 |
| LCK | 1,75 | 3,79E-03 | 10,78 | 2,78 |
| TRIM21 | 1,75 | 5,63E-03 | 61,32 | 17,36 |
| TRAFD1 | 1,75 | 2,78E-03 | 51,46 | 13,73 |
| DSC2 | 1,75 | 6,90E-03 | 16,85 | 5,17 |
| CCDC9 | 1,75 | 5,71E-03 | 19,83 | 5,66 |
| CTSK | 1,75 | 9,70E-03 | 81,08 | 22,19 |
| SP100 | 1,75 | 5,87E-04 | 114,79 | 33,39 |
| NAGK | 1,75 | 1,44E-05 | 199,04 | 74,35 |
| CD8A | 1,75 | 3,88E-03 | 10,69 | 3,00 |
| SMCO4 | 1,76 | 4,09E-03 | 58,30 | 14,22 |
| KDM1B | 1,76 | 3,34E-05 | 10,49 | 3,10 |
| HIPK3 | 1,76 | 4,76E-03 | 41,19 | 11,18 |
| ACTR2 | 1,76 | 2,60E-03 | 178,87 | 45,87 |
| NBEAL2 | 1,76 | 1,53E-03 | 79,29 | 26,64 |
| ATP8B4 | 1,76 | 2,79E-03 | 7,48 | 2,03 |
| ATP6V0A1 | 1,76 | 5,06E-05 | 33,43 | 9,28 |
| CD247 | 1,76 | 2,38E-03 | 11,32 | 3,40 |
| CTSL | 1,76 | 7,58E-03 | 512,13 | 135,12 |
| RASAL3 | 1,76 | 1,54E-05 | 9,01 | 2,53 |
| RAB32 | 1,76 | 2,66E-04 | 30,35 | 8,18 |
| CLCN7 | 1,76 | 2,00E-05 | 46,43 | 10,68 |
| MAPRE2 | 1,76 | 1,40E-06 | 11,51 | 2,96 |
| CCDC93 | 1,77 | 7,76E-04 | 42,52 | 9,36 |
| ZC3H18 | 1,77 | 8,11E-03 | 20,80 | 6,65 |
| TLE4 | 1,77 | 3,02E-03 | 17,95 | 4,60 |
| IL10RB | 1,77 | 1,43E-03 | 96,05 | 25,16 |
| DOT1L | 1,77 | 5,41E-03 | 11,34 | 3,20 |
| SLC15A4 | 1,77 | 3,37E-03 | 46,53 | 12,05 |
| ARHGAP26 | 1,77 | 8,59E-04 | 99,95 | 17,37 |
| NUP98 | 1,77 | 6,64E-03 | 79,71 | 19,59 |
| STAT3 | 1,78 | 8,64E-03 | 173,08 | 45,09 |
| HERC3 | 1,78 | 5,20E-04 | 30,52 | 9,23 |
| P3H2 | 1,78 | 2,79E-03 | 3,33 | 0,85 |
| SMG1P3 | 1,78 | 6,14E-04 | 13,48 | 4,55 |
| PLEKHG2 | 1,78 | 1,97E-03 | 12,94 | 6,24 |
| SIPA1L1 | 1,78 | 3,38E-03 | 52,78 | 14,58 |
| INPP5D | 1,78 | 1,64E-03 | 33,26 | 7,57 |
| TFE3 | 1,78 | 6,70E-03 | 49,99 | 13,12 |
| PTPN12 | 1,78 | 4,65E-03 | 113,98 | 36,08 |
| NLRC3 | 1,79 | 1,52E-03 | 1,74 | 0,46 |
| STAT5B | 1,79 | 3,92E-03 | 65,19 | 15,61 |
| MYH9 | 1,79 | 2,60E-03 | 107,82 | 29,60 |
| CD163L1 | 1,79 | 2,07E-04 | 2,12 | 0,86 |
| TMEM158 | 1,79 | 1,76E-03 | 2,06 | 0,55 |
| IQSEC1 | 1,79 | 3,53E-03 | 49,96 | 12,50 |
| BTN2A1 | 1,79 | 9,79E-04 | 46,71 | 12,84 |
| SYNJ1 | 1,80 | 1,28E-03 | 7,84 | 2,10 |
| PICALM | 1,80 | 9,55E-03 | 267,61 | 63,70 |
| ARPC5 | 1,81 | 5,19E-03 | 212,64 | 56,07 |
| PAK2 | 1,81 | 2,20E-03 | 57,01 | 13,55 |
| MGAT1 | 1,81 | 2,88E-04 | 246,46 | 59,64 |
| LINC00649 | 1,81 | 6,29E-03 | 1,86 | 0,63 |
| TRIM56 | 1,81 | 2,79E-04 | 11,64 | 4,60 |
| SNX18 | 1,81 | 9,19E-05 | 10,68 | 2,78 |
| PRKCD | 1,81 | 1,26E-03 | 101,05 | 25,56 |
| HPS5 | 1,81 | 2,51E-03 | 21,21 | 5,22 |
| MB21D2 | 1,81 | 7,10E-03 | 5,51 | 1,66 |
| TBC1D9 | 1,81 | 4,09E-03 | 14,02 | 4,12 |
| OTUD1 | 1,82 | 3,83E-04 | 11,86 | 3,18 |
| CYP2S1 | 1,82 | 1,42E-03 | 5,87 | 1,72 |
| CD2 | 1,82 | 4,95E-03 | 23,87 | 5,82 |
| NCOA1 | 1,82 | 3,26E-03 | 25,88 | 6,86 |
| AL008729.1 | 1,82 | 2,01E-03 | 2,46 | 0,58 |
| STX16-NPEPL1 | 1,82 | 4,80E-03 | 3,61 | 1,23 |
| USB1 | 1,82 | 1,90E-03 | 68,00 | 15,97 |
| KLHL2 | 1,82 | 5,25E-04 | 40,57 | 9,56 |
| PLEKHM1P1 | 1,83 | 2,92E-05 | 12,28 | 3,16 |
| SLC9A7 | 1,83 | 3,68E-03 | 3,05 | 0,89 |
| ACAP1 | 1,83 | 3,02E-03 | 31,71 | 9,99 |
| RNF157 | 1,83 | 8,33E-03 | 1,74 | 0,41 |
| PLBD2 | 1,83 | 1,25E-04 | 11,81 | 3,14 |
| TMOD2 | 1,83 | 1,94E-03 | 4,09 | 1,14 |
| ABI3 | 1,83 | 7,21E-06 | 8,37 | 1,89 |
| PXDC1 | 1,84 | 2,19E-03 | 10,92 | 3,15 |
| TTYH3 | 1,84 | 9,28E-08 | 30,52 | 8,05 |
| EOMES | 1,84 | 8,04E-03 | 1,81 | 0,42 |
| ZNF200 | 1,84 | 6,45E-03 | 26,29 | 6,26 |
| NEU3 | 1,84 | 2,40E-05 | 5,72 | 1,31 |
| CREBBP | 1,84 | 2,25E-03 | 21,91 | 6,39 |
| SH2D2A | 1,84 | 3,37E-03 | 3,85 | 1,00 |
| RAB33B | 1,84 | 2,41E-03 | 5,49 | 1,42 |
| FTL | 1,84 | 5,35E-03 | 6033,58 | 1453,97 |
| GNS | 1,84 | 1,04E-04 | 114,88 | 31,01 |
| NR3C1 | 1,85 | 6,20E-03 | 33,17 | 8,44 |
| SLC9A9 | 1,85 | 1,97E-03 | 3,69 | 0,79 |
| FRAT2 | 1,85 | 2,10E-04 | 48,37 | 11,91 |
| CORO1C | 1,85 | 7,70E-06 | 86,09 | 19,35 |
| UAP1L1 | 1,85 | 6,48E-06 | 11,23 | 3,03 |
| CD72 | 1,85 | 2,51E-04 | 5,61 | 1,48 |
| SLAMF6 | 1,85 | 4,86E-03 | 6,79 | 1,78 |
| SLC20A1 | 1,85 | 3,71E-04 | 67,90 | 16,32 |
| SLC12A9 | 1,85 | 1,42E-03 | 46,87 | 10,97 |
| CEP55 | 1,85 | 8,14E-07 | 2,37 | 0,56 |
| C17orf62 | 1,86 | 4,08E-04 | 148,83 | 33,91 |
| ALPK1 | 1,86 | 9,86E-04 | 70,95 | 16,06 |
| LOXL3 | 1,86 | 2,87E-07 | 3,68 | 0,99 |
| HLA-A | 1,86 | 3,50E-03 | 1211,17 | 322,22 |
| LBR | 1,86 | 5,60E-03 | 50,85 | 12,05 |
| ARPC1B | 1,86 | 1,82E-04 | 298,41 | 79,43 |
| LTB4R | 1,86 | 1,32E-03 | 17,34 | 4,45 |
| MED13L | 1,86 | 1,19E-03 | 26,57 | 6,99 |
| PDE2A | 1,87 | 6,11E-03 | 2,16 | 0,48 |
| CEP170 | 1,87 | 2,88E-04 | 35,24 | 9,47 |
| MREG | 1,87 | 1,42E-03 | 27,96 | 7,98 |
| RELL1 | 1,87 | 8,20E-03 | 3,06 | 0,79 |
| KAT6A | 1,87 | 2,93E-04 | 23,82 | 5,87 |
| SLA2 | 1,87 | 2,98E-03 | 3,10 | 0,82 |
| ARHGEF6 | 1,87 | 2,72E-04 | 13,02 | 3,05 |
| LONRF3 | 1,88 | 7,45E-03 | 4,49 | 1,13 |
| VASH1 | 1,88 | 3,10E-06 | 14,09 | 3,29 |
| RAC1P2 | 1,88 | 7,54E-03 | 25,41 | 6,14 |
| ADAMTSL4 | 1,88 | 9,37E-07 | 12,33 | 2,90 |
| RFFL | 1,88 | 2,32E-03 | 24,68 | 6,21 |
| PLXND1 | 1,88 | 1,37E-06 | 73,31 | 31,49 |
| TMC8 | 1,89 | 3,09E-06 | 10,94 | 2,27 |
| CD1D | 1,89 | 4,13E-03 | 2,68 | 0,63 |
| EP300 | 1,89 | 4,98E-04 | 19,52 | 5,17 |
| ARHGAP4 | 1,89 | 5,17E-04 | 36,43 | 10,35 |
| RRM2 | 1,89 | 8,14E-07 | 4,73 | 1,06 |
| SPPL2A | 1,89 | 5,20E-04 | 110,22 | 25,87 |
| SNCA | 1,89 | 3,76E-05 | 10,45 | 2,77 |
| TPK1 | 1,90 | 2,86E-05 | 10,67 | 2,98 |
| RPS6KA1 | 1,90 | 5,49E-04 | 98,25 | 22,59 |
| CENPF | 1,90 | 3,20E-08 | 3,12 | 0,67 |
| RIN3 | 1,90 | 8,84E-04 | 15,51 | 4,09 |
| F2R | 1,90 | 2,60E-03 | 2,31 | 0,58 |
| SIPA1L2 | 1,90 | 3,00E-04 | 14,49 | 4,39 |
| BTN2A2 | 1,90 | 4,66E-04 | 21,65 | 5,71 |
| MDFIC | 1,90 | 1,20E-04 | 11,48 | 3,76 |
| CD58 | 1,91 | 4,13E-03 | 86,31 | 20,59 |
| WSB1 | 1,91 | 6,73E-04 | 240,95 | 58,02 |
| SYK | 1,91 | 1,24E-04 | 37,09 | 9,32 |
| NR6A1 | 1,91 | 3,42E-03 | 3,39 | 0,87 |
| MYO9B | 1,91 | 3,07E-04 | 76,53 | 19,25 |
| PCED1B-AS1 | 1,91 | 1,56E-04 | 37,45 | 9,76 |
| AC040162.1 | 1,91 | 4,88E-04 | 3,90 | 1,12 |
| GPAT3 | 1,91 | 6,90E-03 | 16,74 | 3,87 |
| APOL6 | 1,91 | 6,10E-04 | 25,07 | 6,37 |
| RHOQ | 1,92 | 1,78E-03 | 108,01 | 21,53 |
| IL32 | 1,92 | 5,24E-03 | 80,42 | 22,82 |
| MCOLN1 | 1,92 | 3,29E-04 | 30,76 | 7,57 |
| CYLD | 1,92 | 4,83E-03 | 69,03 | 14,41 |
| CEP19 | 1,92 | 7,62E-03 | 10,75 | 2,55 |
| B3GNTL1 | 1,92 | 7,21E-04 | 18,25 | 4,70 |
| MED12 | 1,93 | 8,63E-04 | 20,08 | 5,00 |
| ABCG1 | 1,93 | 6,26E-07 | 31,25 | 7,25 |
| SRGAP2B | 1,93 | 4,71E-04 | 8,98 | 2,14 |
| BOD1L1 | 1,93 | 1,30E-03 | 49,99 | 14,33 |
| TIAM1 | 1,93 | 2,01E-03 | 5,21 | 2,09 |
| RAB33A | 1,94 | 6,83E-03 | 1,76 | 0,41 |
| DENND4B | 1,94 | 6,62E-05 | 49,66 | 11,86 |
| SLC26A11 | 1,94 | 3,07E-06 | 13,74 | 2,90 |
| P2RY8 | 1,94 | 2,00E-03 | 2,15 | 0,49 |
| RFLNB | 1,94 | 6,64E-03 | 7,76 | 2,01 |
| PPP4R1L | 1,94 | 2,45E-04 | 12,28 | 2,77 |
| KLF7 | 1,94 | 5,38E-05 | 15,01 | 3,73 |
| ZMIZ1 | 1,95 | 2,18E-04 | 33,23 | 7,26 |
| EPSTI1 | 1,95 | 1,33E-03 | 31,70 | 6,37 |
| TET2 | 1,95 | 4,05E-03 | 35,90 | 8,17 |
| AGO2 | 1,95 | 7,44E-03 | 14,71 | 3,24 |
| MTFR2 | 1,95 | 7,81E-04 | 2,14 | 0,49 |
| JARID2 | 1,96 | 5,49E-04 | 12,73 | 2,88 |
| HBEGF | 1,96 | 9,02E-03 | 13,04 | 3,58 |
| CALHM2 | 1,96 | 1,81E-06 | 8,70 | 1,88 |
| SH3KBP1 | 1,96 | 2,19E-03 | 31,24 | 6,95 |
| RTN2 | 1,96 | 1,33E-03 | 15,04 | 3,87 |
| SLCO3A1 | 1,96 | 7,94E-03 | 26,15 | 6,08 |
| PSMB8-AS1 | 1,96 | 6,99E-03 | 62,18 | 13,43 |
| HDAC4 | 1,96 | 6,37E-04 | 11,15 | 2,38 |
| PRF1 | 1,96 | 4,32E-03 | 9,30 | 2,27 |
| AC099548.2 | 1,96 | 8,35E-08 | 12,29 | 2,89 |
| LINC00426 | 1,96 | 2,11E-03 | 2,49 | 0,44 |
| IFFO1 | 1,97 | 1,31E-06 | 8,05 | 2,09 |
| IFNAR1 | 1,97 | 1,58E-03 | 68,25 | 13,57 |
| SHKBP1 | 1,97 | 3,80E-03 | 122,93 | 31,88 |
| PLAGL1 | 1,97 | 4,31E-05 | 17,36 | 4,35 |
| STX3 | 1,97 | 5,60E-03 | 96,95 | 22,84 |
| CALHM6 | 1,97 | 8,56E-03 | 23,92 | 5,88 |
| PLD1 | 1,97 | 3,03E-03 | 18,21 | 4,39 |
| ATG7 | 1,97 | 2,60E-03 | 63,65 | 13,15 |
| SGK1 | 1,98 | 1,37E-04 | 372,76 | 83,89 |
| A2M | 1,98 | 1,17E-04 | 59,65 | 27,09 |
| VPS9D1 | 1,98 | 4,34E-04 | 42,02 | 9,58 |
| GRK6 | 1,98 | 4,60E-03 | 39,42 | 8,62 |
| FCHO1 | 1,98 | 4,50E-03 | 7,26 | 1,38 |
| ZC3H3 | 1,98 | 7,38E-04 | 11,47 | 2,62 |
| NINJ2 | 1,98 | 5,89E-05 | 21,35 | 4,88 |
| CAPG | 1,98 | 5,40E-04 | 455,36 | 121,20 |
| CORO7 | 1,99 | 5,06E-05 | 30,45 | 6,30 |
| LYL1 | 1,99 | 6,43E-04 | 8,39 | 1,73 |
| S1PR1 | 1,99 | 7,73E-03 | 2,17 | 0,66 |
| SLC6A6 | 1,99 | 6,97E-04 | 92,97 | 19,15 |
| NRP1 | 1,99 | 2,36E-03 | 18,42 | 5,02 |
| TSPOAP1-AS1 | 1,99 | 3,29E-03 | 2,18 | 0,57 |
| NETO2 | 1,99 | 1,16E-04 | 3,95 | 1,00 |
| LRRC8C | 1,99 | 1,21E-04 | 3,27 | 0,76 |
| ACTB | 1,99 | 6,12E-03 | 3123,75 | 857,57 |
| STAB1 | 2,00 | 3,82E-05 | 41,27 | 15,59 |
| NR2C2 | 2,00 | 3,46E-04 | 10,05 | 2,69 |
| AKAP13 | 2,00 | 3,56E-05 | 64,42 | 19,13 |
| FKBP15 | 2,00 | 2,19E-08 | 51,22 | 11,44 |
| HLA-E | 2,00 | 3,16E-03 | 1110,34 | 245,52 |
| TAPBP | 2,00 | 1,51E-03 | 122,87 | 28,79 |
| SHISAL2A | 2,00 | 2,87E-03 | 2,07 | 0,51 |
| AL669831.1 | 2,00 | 9,07E-03 | 3,08 | 0,74 |
| CD3E | 2,00 | 2,76E-03 | 15,65 | 3,82 |
| BMP2K | 2,00 | 5,96E-08 | 26,99 | 5,14 |
| MMP14 | 2,00 | 1,69E-04 | 100,56 | 25,71 |
| IL7R | 2,01 | 5,68E-03 | 92,90 | 20,50 |
| TRAF3 | 2,01 | 1,21E-04 | 22,65 | 4,34 |
| ADAR | 2,01 | 2,40E-04 | 152,39 | 33,02 |
| TPX2 | 2,01 | 7,22E-04 | 1,79 | 0,41 |
| COL8A2 | 2,01 | 3,71E-03 | 2,31 | 0,55 |
| PDLIM1P4 | 2,01 | 4,20E-03 | 1,93 | 0,46 |
| SEC14L1 | 2,01 | 1,23E-03 | 218,42 | 45,90 |
| ELF1 | 2,01 | 5,35E-04 | 69,97 | 16,39 |
| SELPLG | 2,01 | 9,95E-03 | 84,63 | 17,93 |
| NUMB | 2,01 | 6,47E-03 | 265,44 | 56,45 |
| SEMA4D | 2,01 | 2,07E-04 | 56,08 | 12,01 |
| SIPA1 | 2,02 | 2,57E-03 | 86,24 | 31,94 |
| LINC00900 | 2,02 | 2,00E-07 | 2,47 | 0,60 |
| MERTK | 2,02 | 1,26E-06 | 7,15 | 1,93 |
| CPM | 2,02 | 3,58E-05 | 22,44 | 4,66 |
| TYMP | 2,02 | 1,54E-03 | 192,29 | 44,35 |
| AL390719.1 | 2,02 | 4,48E-03 | 6,76 | 1,54 |
| LAG3 | 2,03 | 1,42E-03 | 3,40 | 0,80 |
| SUSD6 | 2,03 | 2,54E-03 | 104,19 | 24,34 |
| TACC3 | 2,03 | 4,63E-03 | 25,09 | 5,88 |
| SPOCK2 | 2,03 | 1,45E-03 | 10,30 | 2,12 |
| ACAP2 | 2,03 | 3,08E-03 | 33,24 | 7,32 |
| ZNF316 | 2,03 | 5,51E-04 | 20,94 | 4,75 |
| C1QC | 2,03 | 8,08E-07 | 77,06 | 16,40 |
| CTLA4 | 2,03 | 1,33E-03 | 5,74 | 1,39 |
| SNX8 | 2,03 | 5,37E-06 | 27,25 | 5,99 |
| HSPA7 | 2,04 | 4,40E-03 | 16,56 | 3,69 |
| ETS1 | 2,04 | 5,88E-04 | 23,70 | 5,45 |
| MLKL | 2,04 | 1,19E-03 | 46,86 | 10,96 |
| CCDC88B | 2,04 | 1,24E-03 | 77,79 | 24,07 |
| SHCBP1 | 2,04 | 1,82E-04 | 1,68 | 0,52 |
| HECA | 2,05 | 1,58E-03 | 23,53 | 5,26 |
| DUSP6 | 2,05 | 1,43E-05 | 127,83 | 23,88 |
| FAM78A | 2,05 | 7,70E-06 | 4,24 | 0,91 |
| MTMR14 | 2,05 | 8,55E-05 | 42,47 | 10,00 |
| ST20 | 2,05 | 2,28E-03 | 66,08 | 15,01 |
| ITPR2 | 2,05 | 1,19E-04 | 10,88 | 2,51 |
| SLC22A1 | 2,05 | 2,78E-03 | 4,14 | 1,25 |
| JAK2 | 2,05 | 1,14E-03 | 17,77 | 4,56 |
| PDLIM7 | 2,05 | 1,94E-03 | 66,14 | 13,13 |
| ARAP1 | 2,05 | 7,71E-04 | 161,04 | 36,89 |
| RASA3 | 2,05 | 7,80E-08 | 9,63 | 2,16 |
| PLCB2 | 2,05 | 7,63E-04 | 44,23 | 10,06 |
| B3GNT8 | 2,05 | 2,67E-03 | 14,03 | 2,83 |
| CAMKK2 | 2,06 | 1,68E-03 | 46,06 | 10,20 |
| PTK2B | 2,06 | 3,51E-04 | 60,38 | 12,20 |
| TNFAIP2 | 2,06 | 9,75E-03 | 1285,29 | 363,73 |
| SYT11 | 2,06 | 6,63E-04 | 3,58 | 0,78 |
| CD109 | 2,06 | 2,34E-03 | 3,53 | 0,82 |
| NLRP1 | 2,06 | 5,45E-03 | 32,62 | 8,15 |
| RGS19 | 2,06 | 3,80E-03 | 64,63 | 12,76 |
| KIAA0040 | 2,06 | 7,07E-04 | 49,50 | 9,60 |
| PCNX1 | 2,06 | 7,27E-04 | 43,26 | 10,14 |
| CCDC71L | 2,06 | 3,92E-03 | 18,52 | 4,10 |
| SH3BP2 | 2,06 | 1,82E-04 | 84,78 | 14,41 |
| MAN1A1 | 2,07 | 8,39E-04 | 18,27 | 4,18 |
| FNIP1 | 2,07 | 4,44E-03 | 26,19 | 5,49 |
| HIP1 | 2,07 | 4,34E-04 | 24,84 | 6,16 |
| IRF2 | 2,07 | 1,86E-04 | 76,96 | 18,67 |
| TTLL4 | 2,07 | 8,36E-05 | 41,92 | 9,76 |
| MEF2C | 2,08 | 1,02E-04 | 9,16 | 1,79 |
| PTGES3P1 | 2,08 | 4,42E-03 | 37,36 | 8,55 |
| GPRIN3 | 2,08 | 1,70E-04 | 4,75 | 1,08 |
| TNFRSF10D | 2,09 | 5,80E-04 | 25,70 | 5,86 |
| GRAP2 | 2,09 | 2,80E-03 | 3,19 | 0,88 |
| PELI1 | 2,09 | 9,73E-03 | 83,17 | 16,87 |
| VIM | 2,09 | 3,56E-05 | 1068,17 | 242,19 |
| RARA | 2,09 | 3,18E-03 | 56,46 | 12,63 |
| TNIP1 | 2,09 | 7,66E-03 | 271,33 | 62,96 |
| SLC9A8 | 2,09 | 2,10E-04 | 32,55 | 6,68 |
| ZNF710 | 2,09 | 1,38E-04 | 35,98 | 10,15 |
| CMTM3 | 2,09 | 2,18E-08 | 33,41 | 7,30 |
| TGM3 | 2,09 | 2,45E-04 | 9,12 | 1,83 |
| ENG | 2,09 | 5,06E-05 | 18,80 | 4,30 |
| EPB41L3 | 2,10 | 1,63E-03 | 36,60 | 8,01 |
| CISH | 2,10 | 2,34E-03 | 22,85 | 5,58 |
| LPCAT2 | 2,10 | 2,45E-04 | 56,83 | 9,38 |
| CCL22 | 2,10 | 7,62E-04 | 4,86 | 1,15 |
| AKNA | 2,10 | 7,08E-04 | 39,50 | 8,58 |
| DIP2B | 2,10 | 5,20E-04 | 16,91 | 3,81 |
| MOB3C | 2,10 | 6,48E-06 | 34,65 | 7,96 |
| TNFRSF1A | 2,10 | 3,26E-03 | 243,76 | 52,53 |
| PPP1R3B | 2,10 | 9,35E-03 | 52,67 | 10,98 |
| EFHD2 | 2,11 | 6,23E-03 | 208,90 | 42,18 |
| ZNF646 | 2,11 | 1,76E-04 | 12,81 | 2,70 |
| LYSMD2 | 2,11 | 1,12E-03 | 31,44 | 6,38 |
| ELL | 2,11 | 6,40E-03 | 18,53 | 4,07 |
| CXCL16 | 2,11 | 6,16E-04 | 189,67 | 42,76 |
| MYO5A | 2,11 | 4,80E-05 | 12,74 | 3,02 |
| IGFLR1 | 2,11 | 3,20E-05 | 37,22 | 9,06 |
| FILIP1L | 2,11 | 9,00E-04 | 4,12 | 0,92 |
| MRVI1-AS1 | 2,11 | 6,52E-03 | 4,01 | 0,80 |
| VDR | 2,12 | 2,74E-03 | 13,96 | 3,09 |
| NKG7 | 2,12 | 2,71E-03 | 42,86 | 9,35 |
| ARID3A | 2,12 | 1,94E-03 | 17,05 | 3,09 |
| ASGR2 | 2,12 | 2,31E-03 | 2,70 | 0,52 |
| RIPOR1 | 2,12 | 6,22E-06 | 33,27 | 6,44 |
| TSPAN33 | 2,12 | 5,62E-04 | 12,26 | 2,78 |
| TRIM25 | 2,12 | 2,71E-03 | 162,98 | 25,31 |
| ASAP1 | 2,13 | 4,21E-03 | 24,85 | 5,34 |
| BRWD3 | 2,13 | 1,67E-03 | 15,83 | 3,53 |
| FLCN | 2,13 | 2,26E-05 | 42,34 | 8,34 |
| LPXN | 2,13 | 4,65E-03 | 61,07 | 12,29 |
| CD4 | 2,13 | 5,78E-08 | 42,18 | 9,05 |
| IL2RB | 2,13 | 1,07E-04 | 10,33 | 1,73 |
| RAPGEF1 | 2,13 | 2,58E-04 | 38,60 | 8,13 |
| MX1 | 2,13 | 8,09E-05 | 60,99 | 13,14 |
| HELZ2 | 2,14 | 4,14E-03 | 22,44 | 5,23 |
| PID1 | 2,14 | 4,61E-03 | 8,06 | 1,78 |
| SERPINA1 | 2,14 | 2,82E-03 | 1560,61 | 314,14 |
| PLXDC2 | 2,14 | 8,43E-05 | 30,24 | 6,54 |
| ACTN1 | 2,14 | 1,99E-03 | 84,06 | 17,80 |
| RASA4B | 2,14 | 2,51E-03 | 3,42 | 0,88 |
| PHF21A | 2,15 | 7,75E-05 | 46,88 | 9,18 |
| BHLHE40 | 2,15 | 3,19E-03 | 134,74 | 31,49 |
| MFNG | 2,15 | 8,60E-04 | 14,70 | 2,76 |
| STARD4 | 2,15 | 4,06E-03 | 17,98 | 4,05 |
| AC004520.1 | 2,15 | 9,92E-03 | 3,77 | 0,85 |
| GIMAP1 | 2,16 | 4,21E-05 | 3,72 | 0,81 |
| SAMHD1 | 2,16 | 4,08E-07 | 65,25 | 13,71 |
| ANKRD22 | 2,17 | 1,12E-03 | 38,70 | 8,76 |
| MARCH1 | 2,17 | 5,74E-03 | 17,97 | 3,44 |
| OGFRL1 | 2,17 | 7,71E-03 | 31,78 | 5,63 |
| RASSF3 | 2,17 | 5,10E-03 | 65,05 | 12,25 |
| ATP6V1B2 | 2,17 | 4,10E-03 | 351,23 | 68,59 |
| ANKRD44 | 2,17 | 5,00E-03 | 49,21 | 8,84 |
| MTHFD2 | 2,17 | 1,25E-03 | 100,79 | 22,10 |
| AD000671.2 | 2,17 | 4,30E-03 | 3,10 | 0,63 |
| ABL2 | 2,17 | 1,81E-03 | 8,92 | 1,67 |
| NFE2L3 | 2,17 | 7,23E-03 | 14,16 | 3,89 |
| CDKN1A | 2,17 | 1,59E-03 | 139,29 | 31,26 |
| PPARG | 2,17 | 2,59E-05 | 4,84 | 1,05 |
| CPEB4 | 2,17 | 1,88E-03 | 63,66 | 11,19 |
| ZNF385A | 2,17 | 2,22E-05 | 23,95 | 5,15 |
| ZNF697 | 2,18 | 6,25E-04 | 3,20 | 0,67 |
| BTK | 2,18 | 2,05E-03 | 30,66 | 5,23 |
| SLC19A1 | 2,18 | 8,49E-03 | 21,29 | 4,09 |
| TMCC3 | 2,18 | 6,27E-03 | 18,45 | 3,87 |
| PHF19 | 2,18 | 4,34E-05 | 9,79 | 1,85 |
| SLC43A2 | 2,18 | 6,40E-03 | 78,26 | 14,71 |
| B4GALT5 | 2,19 | 6,12E-03 | 96,89 | 21,26 |
| GGT5 | 2,19 | 5,36E-03 | 4,03 | 1,12 |
| CCDC88A | 2,19 | 3,20E-05 | 14,10 | 2,75 |
| PTPRO | 2,19 | 7,37E-05 | 4,82 | 0,85 |
| RHOG | 2,19 | 8,48E-03 | 185,41 | 35,87 |
| CD1C | 2,19 | 2,05E-07 | 9,37 | 1,69 |
| GRB2 | 2,19 | 2,01E-03 | 196,20 | 35,20 |
| RP2 | 2,19 | 5,73E-03 | 27,52 | 5,26 |
| STAT5A | 2,19 | 3,30E-03 | 48,63 | 9,03 |
| FXYD5 | 2,20 | 8,08E-07 | 70,91 | 16,09 |
| GNA13 | 2,20 | 2,81E-03 | 65,82 | 12,26 |
| CELF2 | 2,20 | 1,09E-04 | 38,53 | 7,36 |
| KLHL6 | 2,20 | 9,35E-07 | 16,92 | 3,29 |
| PLAGL2 | 2,20 | 7,25E-03 | 21,19 | 4,60 |
| CD180 | 2,20 | 3,99E-04 | 7,21 | 1,25 |
| HTATSF1P2 | 2,20 | 9,41E-03 | 8,08 | 1,35 |
| GIMAP6 | 2,20 | 1,41E-04 | 11,76 | 2,36 |
| AOAH | 2,20 | 3,19E-03 | 52,18 | 10,76 |
| TCAF2 | 2,21 | 4,35E-04 | 4,21 | 0,89 |
| TPP1 | 2,21 | 8,49E-07 | 155,80 | 28,39 |
| MBOAT7 | 2,21 | 7,32E-03 | 304,49 | 56,31 |
| EAF1 | 2,21 | 7,09E-04 | 21,53 | 4,63 |
| CYFIP2 | 2,22 | 7,22E-04 | 25,30 | 5,38 |
| AC140134.1 | 2,22 | 1,36E-03 | 18,23 | 2,88 |
| RALGDS | 2,22 | 3,20E-03 | 145,36 | 28,67 |
| TNNI2 | 2,22 | 5,41E-04 | 11,58 | 1,82 |
| ARHGEF2 | 2,23 | 5,30E-04 | 66,19 | 15,73 |
| USP32 | 2,23 | 2,98E-03 | 74,33 | 14,69 |
| CRTAM | 2,23 | 4,00E-03 | 3,74 | 0,76 |
| LINC01303 | 2,24 | 3,62E-04 | 3,31 | 0,97 |
| GSAP | 2,24 | 2,45E-04 | 67,80 | 14,82 |
| CAMK4 | 2,24 | 9,09E-03 | 2,23 | 0,32 |
| NPC1 | 2,24 | 5,99E-07 | 64,58 | 15,35 |
| SCD | 2,24 | 2,99E-05 | 31,16 | 6,19 |
| PGS1 | 2,24 | 1,37E-04 | 77,52 | 14,70 |
| TLR7 | 2,24 | 5,25E-04 | 4,00 | 0,73 |
| MYBL1 | 2,24 | 7,60E-03 | 2,17 | 0,38 |
| GLIPR2 | 2,24 | 9,55E-03 | 94,47 | 17,14 |
| WIPF1 | 2,24 | 3,53E-03 | 99,45 | 18,33 |
| XRN1 | 2,24 | 6,03E-04 | 42,07 | 6,41 |
| MAN2B1 | 2,25 | 6,80E-08 | 81,40 | 20,67 |
| MSN | 2,25 | 5,80E-04 | 223,14 | 45,07 |
| STK4 | 2,25 | 7,19E-04 | 53,43 | 10,16 |
| ZYX | 2,25 | 9,91E-04 | 177,25 | 33,14 |
| PARVG | 2,25 | 8,75E-05 | 64,57 | 12,52 |
| FAM105A | 2,25 | 1,83E-05 | 8,41 | 1,54 |
| ESPL1 | 2,26 | 1,72E-05 | 1,72 | 0,29 |
| PRKCB | 2,26 | 4,03E-03 | 29,18 | 4,74 |
| UPP1 | 2,26 | 2,89E-05 | 70,29 | 15,96 |
| PPP1R16B | 2,26 | 9,03E-04 | 4,18 | 0,92 |
| HIST2H2BF | 2,26 | 2,93E-04 | 15,44 | 2,86 |
| ITGAL | 2,27 | 7,52E-04 | 25,96 | 5,11 |
| DTX3L | 2,27 | 7,77E-05 | 41,11 | 8,41 |
| FLI1 | 2,27 | 1,52E-03 | 26,50 | 4,96 |
| TMEM106A | 2,27 | 2,41E-04 | 7,89 | 1,32 |
| PSTPIP1 | 2,27 | 6,02E-04 | 28,57 | 6,19 |
| ANTXR2 | 2,27 | 4,43E-03 | 81,67 | 13,46 |
| RN7SKP176 | 2,27 | 2,07E-04 | 3,96 | 0,79 |
| NRM | 2,27 | 3,05E-05 | 9,78 | 1,85 |
| TNFSF15 | 2,28 | 2,51E-04 | 14,96 | 2,68 |
| AL133330.1 | 2,28 | 9,30E-03 | 5,00 | 0,94 |
| DOK2 | 2,28 | 1,70E-08 | 13,25 | 2,44 |
| SKAP2 | 2,28 | 3,23E-04 | 66,05 | 11,71 |
| HCLS1 | 2,28 | 5,25E-03 | 303,15 | 51,33 |
| MSR1 | 2,28 | 3,87E-05 | 34,82 | 6,49 |
| NPL | 2,28 | 3,53E-03 | 88,65 | 15,88 |
| PSAP | 2,29 | 5,92E-06 | 1288,67 | 263,10 |
| MMP19 | 2,29 | 1,24E-03 | 18,20 | 3,78 |
| RAP2C | 2,29 | 3,35E-03 | 61,12 | 13,52 |
| SMCR8 | 2,29 | 5,42E-06 | 10,15 | 2,05 |
| EMP3 | 2,29 | 1,33E-03 | 224,10 | 41,67 |
| ADAP2 | 2,29 | 2,19E-08 | 13,15 | 2,61 |
| MATK | 2,29 | 1,81E-06 | 7,79 | 1,51 |
| EPG5 | 2,29 | 8,36E-05 | 17,78 | 4,51 |
| HIVEP1 | 2,29 | 6,77E-04 | 38,51 | 8,97 |
| RASGRP1 | 2,29 | 1,03E-03 | 5,91 | 1,36 |
| RASGRP3 | 2,29 | 4,89E-07 | 8,24 | 1,55 |
| LYST | 2,29 | 8,68E-03 | 65,55 | 11,91 |
| FCHSD2 | 2,29 | 2,78E-05 | 35,17 | 5,70 |
| ZMYND15 | 2,30 | 2,44E-05 | 5,71 | 1,01 |
| ARHGAP22 | 2,30 | 1,52E-06 | 7,74 | 1,23 |
| AL353625.1 | 2,30 | 1,25E-05 | 3,44 | 0,66 |
| IRF5 | 2,31 | 3,26E-06 | 18,48 | 3,38 |
| SSH2 | 2,31 | 1,97E-03 | 48,95 | 11,83 |
| PLCG2 | 2,31 | 2,09E-04 | 38,61 | 6,93 |
| ATF5 | 2,31 | 5,60E-05 | 48,87 | 9,18 |
| IL12RB1 | 2,31 | 1,60E-04 | 5,37 | 0,95 |
| SPN | 2,31 | 3,32E-04 | 8,45 | 1,37 |
| TET3 | 2,31 | 2,96E-04 | 12,50 | 2,36 |
| CLIC4 | 2,32 | 4,95E-03 | 79,06 | 15,35 |
| AL365361.1 | 2,32 | 6,84E-03 | 2,13 | 0,43 |
| LAIR1 | 2,32 | 5,38E-05 | 70,18 | 12,43 |
| TMEM131L | 2,32 | 1,27E-04 | 6,10 | 1,24 |
| KIF21B | 2,32 | 6,16E-04 | 5,17 | 0,96 |
| FYN | 2,32 | 9,66E-06 | 35,61 | 6,98 |
| TMPPE | 2,32 | 1,58E-04 | 2,22 | 0,47 |
| GSDME | 2,32 | 1,10E-05 | 20,39 | 4,25 |
| KLF6 | 2,33 | 3,17E-06 | 122,16 | 22,35 |
| UNC13D | 2,33 | 4,44E-04 | 38,06 | 8,74 |
| RFTN1 | 2,33 | 6,57E-04 | 17,30 | 3,30 |
| ARHGAP9 | 2,34 | 3,79E-03 | 172,75 | 31,77 |
| RNF144B | 2,34 | 4,07E-03 | 45,93 | 8,71 |
| CACNA2D4 | 2,34 | 1,52E-04 | 15,09 | 2,80 |
| ZNFX1 | 2,34 | 3,46E-04 | 37,58 | 7,16 |
| NCAPH | 2,34 | 5,94E-05 | 1,69 | 0,43 |
| ADCY3 | 2,34 | 4,98E-07 | 27,07 | 4,61 |
| BMP6 | 2,34 | 1,13E-03 | 1,98 | 0,32 |
| UBE2D1 | 2,34 | 7,15E-03 | 53,01 | 8,80 |
| CCR6 | 2,35 | 9,35E-07 | 3,99 | 0,69 |
| COTL1 | 2,35 | 5,82E-03 | 239,12 | 40,51 |
| C5AR2 | 2,35 | 2,98E-03 | 14,69 | 2,58 |
| TM6SF1 | 2,35 | 2,04E-04 | 14,35 | 2,77 |
| SLC16A6 | 2,35 | 8,28E-06 | 10,36 | 1,55 |
| FSCN1 | 2,35 | 2,11E-03 | 12,62 | 2,70 |
| FNDC3B | 2,35 | 2,96E-04 | 95,85 | 17,57 |
| CD5 | 2,35 | 4,48E-04 | 3,43 | 0,82 |
| NADK | 2,35 | 7,52E-04 | 202,47 | 33,67 |
| SLCO2B1 | 2,35 | 1,26E-06 | 14,38 | 2,64 |
| TRPV2 | 2,36 | 2,22E-05 | 25,53 | 7,43 |
| HMGN2P46 | 2,36 | 2,98E-03 | 8,44 | 1,83 |
| TLE3 | 2,36 | 2,81E-03 | 100,55 | 16,68 |
| RAB20 | 2,36 | 6,17E-04 | 88,89 | 17,15 |
| IRF7 | 2,36 | 6,28E-04 | 79,85 | 14,50 |
| IRF8 | 2,36 | 8,36E-05 | 23,67 | 3,73 |
| PARVB | 2,36 | 1,40E-06 | 21,94 | 3,27 |
| CLEC10A | 2,36 | 2,88E-03 | 24,98 | 3,92 |
| MICAL1 | 2,37 | 5,60E-10 | 41,28 | 6,69 |
| NOTCH1 | 2,37 | 4,90E-03 | 20,02 | 3,46 |
| RELT | 2,37 | 5,84E-03 | 24,66 | 4,15 |
| CD300LF | 2,37 | 5,68E-03 | 26,90 | 3,79 |
| PLSCR1 | 2,37 | 1,20E-03 | 473,83 | 79,38 |
| E2F3 | 2,37 | 1,16E-03 | 17,26 | 3,03 |
| VCAN | 2,37 | 1,91E-03 | 79,07 | 14,13 |
| CD44 | 2,38 | 1,82E-04 | 848,22 | 152,08 |
| BCL6 | 2,38 | 2,05E-03 | 309,84 | 50,73 |
| TCP11L1 | 2,38 | 2,06E-04 | 6,19 | 1,39 |
| DAB2 | 2,38 | 7,46E-10 | 36,71 | 6,07 |
| MOB3A | 2,38 | 1,49E-03 | 79,19 | 13,95 |
| LSP1 | 2,38 | 6,43E-03 | 288,95 | 46,05 |
| POU2F2 | 2,38 | 2,96E-05 | 6,90 | 1,48 |
| SH2B3 | 2,38 | 1,01E-04 | 20,02 | 3,62 |
| HCG27 | 2,39 | 4,79E-07 | 16,32 | 2,30 |
| DOCK5 | 2,39 | 3,39E-04 | 49,42 | 7,11 |
| NFKBIE | 2,39 | 8,60E-03 | 73,14 | 16,37 |
| GFI1 | 2,39 | 4,29E-04 | 2,15 | 0,36 |
| ADAM8 | 2,40 | 9,50E-03 | 173,71 | 28,03 |
| ZNF438 | 2,40 | 4,56E-03 | 41,99 | 6,99 |
| LTB | 2,40 | 5,09E-03 | 99,92 | 16,47 |
| DENND1C | 2,40 | 1,50E-05 | 12,57 | 2,06 |
| SLC22A18AS | 2,40 | 8,81E-03 | 2,01 | 0,45 |
| CSGALNACT2 | 2,40 | 5,17E-04 | 34,50 | 5,91 |
| FGD3 | 2,40 | 1,88E-03 | 49,11 | 7,28 |
| PATL2 | 2,41 | 1,82E-04 | 9,84 | 2,12 |
| CARD8 | 2,41 | 2,19E-05 | 97,22 | 17,41 |
| KREMEN1 | 2,41 | 3,05E-05 | 21,30 | 3,79 |
| PRAM1 | 2,41 | 3,31E-08 | 13,08 | 2,49 |
| TLN1 | 2,41 | 3,29E-04 | 152,81 | 29,45 |
| LINC01347 | 2,41 | 1,67E-03 | 22,40 | 3,68 |
| XPO6 | 2,41 | 2,85E-03 | 274,44 | 46,35 |
| GPSM3 | 2,41 | 8,17E-03 | 168,86 | 26,82 |
| PRDM1 | 2,41 | 6,06E-04 | 14,02 | 2,77 |
| RCSD1 | 2,42 | 9,33E-04 | 20,81 | 3,40 |
| ARSB | 2,42 | 5,43E-06 | 6,35 | 1,31 |
| ALOX5 | 2,42 | 5,46E-04 | 132,66 | 21,13 |
| TECPR2 | 2,42 | 4,44E-03 | 46,47 | 5,48 |
| ARHGAP45 | 2,42 | 9,90E-04 | 60,61 | 11,17 |
| LAT2 | 2,43 | 6,06E-03 | 100,48 | 15,46 |
| TMEM88 | 2,43 | 5,27E-03 | 9,39 | 1,70 |
| LIMK2 | 2,43 | 9,31E-03 | 313,56 | 50,53 |
| ARRB1 | 2,43 | 5,39E-05 | 21,43 | 3,98 |
| TRAF3IP3 | 2,44 | 5,39E-05 | 39,84 | 7,75 |
| AC106028.3 | 2,44 | 6,54E-04 | 2,16 | 0,38 |
| RNF149 | 2,44 | 4,28E-03 | 319,22 | 55,48 |
| ARRB2 | 2,44 | 4,44E-03 | 343,33 | 48,61 |
| IL17RA | 2,44 | 5,11E-05 | 28,67 | 4,79 |
| VSIR | 2,44 | 1,94E-03 | 152,57 | 24,59 |
| CYP27A1 | 2,44 | 2,26E-04 | 75,57 | 13,88 |
| ELMO1 | 2,44 | 3,26E-03 | 14,59 | 3,13 |
| FANCA | 2,44 | 3,20E-08 | 10,41 | 2,30 |
| MKI67 | 2,44 | 4,37E-09 | 2,04 | 0,47 |
| LIMS1 | 2,44 | 1,09E-03 | 100,67 | 18,51 |
| WDFY3 | 2,44 | 1,51E-04 | 58,09 | 8,62 |
| CX3CR1 | 2,44 | 1,06E-09 | 13,37 | 2,26 |
| AP1S2 | 2,45 | 4,29E-04 | 60,86 | 10,19 |
| FLNA | 2,46 | 3,04E-05 | 184,59 | 44,27 |
| EMILIN2 | 2,46 | 1,12E-04 | 25,41 | 6,94 |
| PPM1N | 2,46 | 1,41E-04 | 3,61 | 0,78 |
| VENTX | 2,46 | 3,51E-04 | 2,91 | 0,49 |
| DAPP1 | 2,46 | 1,08E-03 | 120,55 | 20,22 |
| GAB2 | 2,47 | 2,94E-03 | 31,85 | 5,04 |
| TNFAIP8L2 | 2,47 | 1,97E-03 | 36,65 | 5,61 |
| ERMN | 2,47 | 1,80E-04 | 3,43 | 0,65 |
| CKLF-CMTM1 | 2,47 | 2,05E-03 | 7,19 | 1,19 |
| AP5B1 | 2,47 | 5,50E-05 | 18,65 | 3,13 |
| HLA-F | 2,47 | 1,17E-03 | 238,86 | 43,29 |
| KLHL5 | 2,47 | 3,32E-05 | 27,90 | 4,95 |
| IL6R | 2,47 | 2,05E-03 | 57,98 | 9,02 |
| IL21R | 2,48 | 4,16E-05 | 3,03 | 0,57 |
| SLC25A37 | 2,48 | 2,54E-03 | 337,17 | 73,47 |
| IGF2R | 2,48 | 8,88E-04 | 78,26 | 13,53 |
| RASSF2 | 2,48 | 8,68E-03 | 101,00 | 14,97 |
| MTF1 | 2,48 | 5,92E-03 | 24,92 | 4,61 |
| RHBDF2 | 2,48 | 4,80E-05 | 69,35 | 11,33 |
| FCGR2C | 2,48 | 3,33E-03 | 48,50 | 8,18 |
| ITGB2-AS1 | 2,49 | 2,88E-04 | 28,08 | 4,59 |
| AC011899.2 | 2,49 | 2,82E-08 | 2,02 | 0,28 |
| GIMAP5 | 2,49 | 2,09E-04 | 37,26 | 6,95 |
| AL121603.2 | 2,50 | 4,44E-04 | 3,94 | 0,67 |
| PLEKHO2 | 2,50 | 1,62E-03 | 101,29 | 15,50 |
| GNB4 | 2,50 | 5,60E-05 | 18,70 | 3,22 |
| CD28 | 2,50 | 6,57E-04 | 4,54 | 1,11 |
| TTYH2 | 2,50 | 3,83E-04 | 6,43 | 1,08 |
| GRAMD1A | 2,50 | 2,53E-03 | 199,95 | 30,62 |
| PREX1 | 2,51 | 2,44E-03 | 96,78 | 15,04 |
| LYZ | 2,51 | 4,44E-03 | 1123,31 | 170,13 |
| CHST11 | 2,51 | 9,70E-04 | 29,40 | 4,99 |
| RENBP | 2,51 | 4,22E-04 | 21,35 | 3,40 |
| RNF213 | 2,51 | 8,66E-06 | 147,39 | 28,53 |
| SP110 | 2,52 | 6,72E-04 | 98,44 | 15,53 |
| MYO1F | 2,52 | 6,78E-03 | 197,92 | 26,14 |
| GRINA | 2,52 | 4,80E-05 | 351,25 | 56,48 |
| DENND5A | 2,52 | 3,59E-03 | 185,13 | 31,03 |
| PTPN6 | 2,52 | 5,60E-05 | 190,10 | 35,58 |
| SLC8A1 | 2,52 | 2,32E-03 | 13,71 | 2,21 |
| SYNE3 | 2,53 | 5,79E-04 | 1,72 | 0,29 |
| DSE | 2,53 | 1,46E-03 | 57,55 | 9,38 |
| CD1A | 2,53 | 1,90E-06 | 5,14 | 0,74 |
| SLC7A11 | 2,53 | 1,49E-03 | 6,96 | 1,10 |
| PIK3CD | 2,53 | 1,46E-03 | 40,90 | 6,31 |
| SPHK1 | 2,53 | 7,16E-03 | 17,66 | 2,77 |
| RAB42 | 2,54 | 1,08E-06 | 6,73 | 0,96 |
| KCTD12 | 2,54 | 3,02E-11 | 20,46 | 3,25 |
| ADCY7 | 2,54 | 1,33E-13 | 18,60 | 2,91 |
| LAPTM5 | 2,54 | 1,99E-03 | 635,44 | 95,38 |
| FAM72A | 2,54 | 3,10E-04 | 1,87 | 0,30 |
| AC073046.1 | 2,54 | 2,78E-03 | 6,58 | 1,03 |
| MAFB | 2,55 | 2,05E-07 | 29,81 | 4,87 |
| HAVCR2 | 2,55 | 8,68E-08 | 23,12 | 3,69 |
| TMEM154 | 2,55 | 8,20E-03 | 48,58 | 6,99 |
| TAP2 | 2,55 | 5,60E-05 | 53,04 | 12,46 |
| BRCA2 | 2,55 | 1,44E-04 | 1,95 | 0,23 |
| AL627309.6 | 2,55 | 8,60E-03 | 159,70 | 23,22 |
| QKI | 2,55 | 4,24E-04 | 91,07 | 15,27 |
| SPP1 | 2,55 | 3,26E-03 | 2518,33 | 364,84 |
| OLFML2B | 2,55 | 8,75E-09 | 10,10 | 2,53 |
| AC138035.1 | 2,56 | 7,84E-04 | 103,57 | 15,43 |
| CXorf21 | 2,56 | 4,43E-03 | 16,50 | 2,26 |
| LACTB | 2,56 | 2,83E-04 | 52,33 | 8,08 |
| IFIT2 | 2,56 | 6,40E-03 | 104,90 | 14,17 |
| MPEG1 | 2,56 | 3,34E-05 | 65,91 | 9,81 |
| ASGR1 | 2,57 | 1,68E-04 | 3,01 | 0,44 |
| STARD8 | 2,58 | 3,11E-04 | 3,42 | 0,55 |
| SLC36A1 | 2,58 | 3,26E-08 | 8,76 | 1,40 |
| NCF4 | 2,58 | 5,65E-03 | 158,59 | 22,32 |
| FLT1 | 2,58 | 5,00E-05 | 6,39 | 1,01 |
| SLFN12L | 2,58 | 1,57E-03 | 3,91 | 0,91 |
| ST8SIA4 | 2,59 | 9,12E-03 | 24,67 | 3,60 |
| SLC9B2 | 2,59 | 2,07E-04 | 8,88 | 1,06 |
| LINC00861 | 2,59 | 1,58E-03 | 4,97 | 0,83 |
| MYD88 | 2,60 | 2,04E-04 | 178,81 | 27,21 |
| DOCK8 | 2,60 | 1,66E-03 | 47,75 | 7,02 |
| PDSS1 | 2,60 | 2,30E-03 | 13,56 | 2,18 |
| AC135050.5 | 2,60 | 9,15E-03 | 2,63 | 0,41 |
| PLA2G4C | 2,60 | 1,48E-05 | 6,95 | 1,65 |
| PILRA | 2,60 | 8,87E-03 | 206,87 | 30,21 |
| ITGA4 | 2,61 | 2,73E-05 | 20,30 | 3,18 |
| LINC01094 | 2,61 | 7,14E-04 | 19,19 | 2,73 |
| HLX | 2,61 | 7,90E-03 | 16,86 | 2,56 |
| VSIG4 | 2,61 | 1,38E-04 | 42,70 | 5,95 |
| IL4I1 | 2,61 | 6,57E-04 | 58,69 | 9,98 |
| RELB | 2,62 | 1,63E-03 | 67,36 | 10,67 |
| MKNK1 | 2,62 | 2,92E-11 | 129,93 | 17,64 |
| ELOVL7 | 2,62 | 9,21E-03 | 6,36 | 1,06 |
| ATP13A3 | 2,62 | 7,66E-04 | 171,54 | 28,30 |
| BCL2 | 2,62 | 7,21E-06 | 6,16 | 0,94 |
| NLRC4 | 2,62 | 2,38E-03 | 20,83 | 2,85 |
| IFIH1 | 2,62 | 1,82E-04 | 54,12 | 7,93 |
| LY9 | 2,63 | 1,26E-06 | 10,19 | 1,56 |
| CGAS | 2,63 | 6,48E-06 | 5,03 | 0,75 |
| MPP1 | 2,63 | 1,28E-03 | 85,93 | 11,69 |
| CCL7 | 2,63 | 7,96E-03 | 5,92 | 0,94 |
| LYN | 2,63 | 5,83E-03 | 441,11 | 61,76 |
| CCR4 | 2,63 | 2,04E-04 | 3,86 | 0,62 |
| ARL11 | 2,64 | 8,42E-03 | 16,85 | 1,84 |
| LINC00884 | 2,64 | 5,52E-05 | 2,50 | 0,53 |
| NCKAP1L | 2,64 | 2,36E-06 | 39,26 | 5,38 |
| ITPR1 | 2,64 | 4,79E-07 | 13,62 | 3,34 |
| FPR3 | 2,64 | 2,79E-04 | 56,95 | 7,97 |
| ITGA7 | 2,64 | 2,20E-05 | 7,02 | 1,17 |
| TRPM2 | 2,64 | 1,52E-05 | 13,91 | 2,32 |
| PLEKHO1 | 2,64 | 5,92E-06 | 73,69 | 11,17 |
| HSPA6 | 2,64 | 5,80E-03 | 69,15 | 9,36 |
| CD300E | 2,65 | 9,02E-03 | 48,41 | 7,71 |
| SECTM1 | 2,65 | 5,76E-03 | 182,48 | 27,27 |
| TNFSF8 | 2,65 | 8,23E-03 | 19,60 | 2,70 |
| SLC12A6 | 2,65 | 6,74E-04 | 90,04 | 12,89 |
| CCR7 | 2,65 | 2,56E-03 | 22,89 | 3,84 |
| CBL | 2,65 | 2,44E-05 | 16,00 | 2,40 |
| FOXP3 | 2,66 | 1,18E-06 | 2,10 | 0,32 |
| LHFPL2 | 2,66 | 2,18E-08 | 29,08 | 4,51 |
| ZC3H12D | 2,66 | 9,35E-07 | 6,77 | 0,99 |
| ELF4 | 2,66 | 9,38E-05 | 27,50 | 4,18 |
| ABCA1 | 2,66 | 5,57E-07 | 21,29 | 3,16 |
| ARHGAP30 | 2,66 | 9,30E-04 | 82,97 | 11,47 |
| GIMAP8 | 2,66 | 3,93E-06 | 5,43 | 0,87 |
| AC004687.1 | 2,66 | 5,15E-05 | 19,69 | 2,67 |
| AC008105.3 | 2,66 | 7,75E-05 | 26,24 | 3,83 |
| CDH23 | 2,66 | 2,66E-05 | 4,64 | 0,72 |
| SLC11A1 | 2,66 | 7,12E-03 | 286,78 | 35,48 |
| P2RX1 | 2,66 | 2,02E-03 | 9,53 | 1,27 |
| CHST15 | 2,66 | 8,02E-05 | 73,54 | 11,05 |
| ADAM19 | 2,66 | 4,88E-04 | 12,31 | 1,86 |
| ADGRE5 | 2,67 | 5,14E-04 | 213,02 | 27,94 |
| KCNAB2 | 2,67 | 3,37E-08 | 34,26 | 4,52 |
| DOCK2 | 2,67 | 3,62E-04 | 34,68 | 5,53 |
| CCM2L | 2,67 | 8,46E-03 | 3,37 | 0,53 |
| PIM1 | 2,67 | 1,31E-06 | 88,21 | 12,13 |
| SLC37A2 | 2,67 | 3,01E-11 | 11,61 | 1,93 |
| TLR6 | 2,68 | 5,27E-03 | 25,59 | 3,18 |
| CSF1R | 2,68 | 2,19E-08 | 57,38 | 8,18 |
| TLR10 | 2,68 | 1,97E-03 | 6,18 | 0,73 |
| TESPA1 | 2,68 | 6,48E-06 | 4,91 | 1,01 |
| PTPN7 | 2,68 | 1,18E-06 | 19,14 | 2,55 |
| RIPK2 | 2,68 | 5,74E-04 | 57,24 | 8,83 |
| TRANK1 | 2,68 | 3,41E-06 | 46,59 | 6,29 |
| CTRL | 2,68 | 1,50E-04 | 4,63 | 0,95 |
| LRRC25 | 2,69 | 1,22E-03 | 51,07 | 6,60 |
| HERC5 | 2,70 | 2,26E-04 | 14,74 | 2,03 |
| FNIP2 | 2,70 | 1,30E-05 | 25,48 | 4,10 |
| SH2D3C | 2,70 | 1,14E-05 | 14,63 | 1,89 |
| ARHGAP25 | 2,70 | 1,94E-03 | 89,72 | 11,26 |
| STRIP2 | 2,70 | 2,33E-05 | 2,56 | 0,43 |
| IKZF1 | 2,71 | 1,07E-05 | 19,31 | 2,85 |
| IL10RA | 2,71 | 2,63E-04 | 56,83 | 7,67 |
| MCOLN2 | 2,71 | 1,72E-03 | 21,38 | 2,86 |
| WDFY4 | 2,71 | 2,06E-04 | 12,79 | 2,08 |
| TIGIT | 2,71 | 1,04E-04 | 7,33 | 0,99 |
| ARID5A | 2,71 | 4,33E-05 | 59,23 | 8,62 |
| CMTM1 | 2,71 | 4,06E-05 | 10,16 | 1,56 |
| SMG1P1 | 2,72 | 2,87E-07 | 16,01 | 2,57 |
| AMPD3 | 2,72 | 5,11E-06 | 68,88 | 9,40 |
| PARP14 | 2,72 | 4,76E-05 | 88,90 | 13,23 |
| IRF4 | 2,72 | 4,46E-05 | 4,08 | 0,62 |
| DRAM1 | 2,72 | 1,12E-03 | 102,41 | 12,92 |
| AC012368.1 | 2,72 | 4,56E-03 | 51,88 | 6,26 |
| AL078604.2 | 2,73 | 9,41E-03 | 4,05 | 0,54 |
| NFKB2 | 2,73 | 3,50E-03 | 141,54 | 19,85 |
| OASL | 2,73 | 5,74E-04 | 14,74 | 1,94 |
| CD37 | 2,73 | 5,36E-04 | 136,04 | 17,06 |
| ACP5 | 2,73 | 3,41E-06 | 116,80 | 16,67 |
| MX2 | 2,73 | 5,10E-03 | 113,45 | 14,22 |
| AC025580.2 | 2,73 | 4,56E-03 | 7,22 | 1,31 |
| STK10 | 2,73 | 7,97E-04 | 76,54 | 10,73 |
| SIGLEC15 | 2,74 | 8,36E-05 | 6,25 | 0,88 |
| NABP1 | 2,74 | 1,62E-03 | 348,57 | 50,99 |
| AL732372.2 | 2,74 | 3,07E-04 | 152,33 | 17,73 |
| C1QTNF1 | 2,74 | 2,52E-03 | 4,26 | 0,77 |
| WARS | 2,74 | 2,32E-05 | 332,59 | 40,58 |
| LAMP3 | 2,74 | 3,12E-04 | 21,00 | 3,29 |
| SEMA4A | 2,74 | 1,14E-05 | 64,05 | 9,15 |
| PLB1 | 2,74 | 3,34E-06 | 18,93 | 2,54 |
| VAV1 | 2,74 | 8,18E-03 | 95,52 | 12,49 |
| SIGLEC7 | 2,74 | 6,87E-05 | 15,27 | 2,04 |
| WAS | 2,75 | 2,80E-03 | 67,60 | 8,86 |
| SCIMP | 2,75 | 3,94E-07 | 11,74 | 1,64 |
| EHBP1L1 | 2,75 | 8,17E-06 | 150,61 | 22,83 |
| SLC1A3 | 2,76 | 1,23E-05 | 58,07 | 7,57 |
| GHRL | 2,76 | 1,60E-06 | 8,60 | 1,67 |
| ADM | 2,76 | 3,35E-03 | 169,03 | 23,02 |
| ST3GAL2 | 2,76 | 6,02E-04 | 23,23 | 2,79 |
| TGFBI | 2,76 | 1,02E-10 | 201,49 | 33,65 |
| TNFRSF4 | 2,77 | 3,91E-03 | 12,43 | 1,92 |
| CST7 | 2,77 | 1,89E-03 | 81,18 | 10,15 |
| SLC15A3 | 2,77 | 1,13E-03 | 201,69 | 28,79 |
| NLRC5 | 2,77 | 8,08E-07 | 43,91 | 7,09 |
| TAP1 | 2,77 | 8,52E-05 | 208,29 | 30,29 |
| ITGA5 | 2,78 | 4,30E-05 | 46,18 | 5,66 |
| DENND3 | 2,78 | 1,07E-04 | 134,36 | 14,16 |
| LILRB1 | 2,78 | 3,96E-03 | 36,01 | 5,49 |
| THEMIS2 | 2,79 | 3,57E-04 | 177,15 | 25,28 |
| CLEC4D | 2,79 | 9,15E-03 | 59,39 | 8,57 |
| GCH1 | 2,79 | 7,42E-03 | 76,40 | 10,20 |
| STAT1 | 2,79 | 2,52E-06 | 154,89 | 20,18 |
| RNF24 | 2,79 | 3,12E-04 | 42,86 | 5,71 |
| ICOS | 2,79 | 2,94E-04 | 4,76 | 0,63 |
| GBGT1 | 2,80 | 2,64E-06 | 13,76 | 1,89 |
| PDE1B | 2,80 | 2,64E-07 | 2,06 | 0,32 |
| AC011498.4 | 2,80 | 4,08E-04 | 5,14 | 0,69 |
| CD300C | 2,80 | 1,77E-04 | 7,68 | 1,11 |
| GMIP | 2,80 | 1,23E-04 | 51,87 | 6,12 |
| IL2RG | 2,80 | 9,02E-04 | 294,40 | 36,25 |
| TGFB1 | 2,81 | 2,70E-04 | 50,99 | 6,74 |
| PADI2 | 2,81 | 4,08E-03 | 34,89 | 4,30 |
| CCL24 | 2,81 | 1,31E-03 | 9,38 | 1,08 |
| NLRP3 | 2,81 | 5,10E-03 | 24,33 | 3,09 |
| FCGR2B | 2,81 | 3,26E-08 | 76,72 | 9,71 |
| PTPRE | 2,81 | 3,33E-03 | 95,54 | 12,00 |
| LRFN1 | 2,82 | 1,06E-03 | 2,63 | 0,33 |
| OSCAR | 2,82 | 1,52E-04 | 29,75 | 3,95 |
| BTNL8 | 2,82 | 3,14E-03 | 27,32 | 3,05 |
| HS3ST3B1 | 2,83 | 6,76E-04 | 8,95 | 1,35 |
| APOL3 | 2,83 | 2,18E-04 | 33,11 | 4,40 |
| AC107959.3 | 2,83 | 6,05E-03 | 5,93 | 0,71 |
| NRIP3 | 2,83 | 1,11E-04 | 5,36 | 0,97 |
| COLEC12 | 2,83 | 8,68E-08 | 3,44 | 0,40 |
| AL391825.1 | 2,83 | 3,62E-03 | 2,18 | 0,29 |
| DOCK10 | 2,84 | 1,34E-06 | 21,71 | 2,96 |
| CLEC5A | 2,84 | 2,67E-03 | 66,79 | 7,80 |
| FLVCR2 | 2,85 | 1,69E-09 | 9,03 | 1,13 |
| CHST2 | 2,85 | 6,53E-05 | 10,50 | 1,50 |
| BEST1 | 2,85 | 5,21E-03 | 27,28 | 3,08 |
| AC245297.1 | 2,85 | 4,27E-04 | 23,21 | 3,10 |
| CD80 | 2,85 | 7,32E-03 | 8,71 | 1,20 |
| DMXL2 | 2,86 | 2,96E-04 | 155,02 | 22,10 |
| AC083862.2 | 2,87 | 3,18E-03 | 16,91 | 1,89 |
| AC090559.1 | 2,87 | 4,40E-03 | 31,95 | 3,57 |
| CD209 | 2,87 | 9,60E-07 | 2,59 | 0,32 |
| FTH1P16 | 2,87 | 7,67E-03 | 3,52 | 0,48 |
| RN7SKP296 | 2,87 | 6,06E-03 | 5,73 | 0,69 |
| IL1RAP | 2,87 | 1,23E-03 | 84,85 | 10,71 |
| AL844908.1 | 2,88 | 2,63E-04 | 4,55 | 0,61 |
| CHI3L1 | 2,88 | 7,95E-03 | 283,32 | 30,05 |
| HK3 | 2,89 | 2,86E-05 | 35,70 | 4,14 |
| SLC7A7 | 2,89 | 2,09E-05 | 59,94 | 7,98 |
| IRF1 | 2,89 | 6,22E-04 | 342,33 | 37,93 |
| PKMYT1 | 2,89 | 7,06E-03 | 1,75 | 0,31 |
| CXCR4 | 2,90 | 1,98E-03 | 830,00 | 89,40 |
| P2RY13 | 2,90 | 5,02E-03 | 100,00 | 10,93 |
| CREB5 | 2,90 | 3,38E-03 | 52,28 | 6,79 |
| BACH1 | 2,90 | 6,87E-04 | 91,75 | 11,54 |
| PPP1R18 | 2,90 | 2,26E-03 | 259,00 | 32,12 |
| FMNL1 | 2,90 | 1,40E-03 | 160,09 | 17,70 |
| ITK | 2,90 | 5,34E-05 | 11,66 | 1,47 |
| CCR1 | 2,91 | 1,65E-03 | 208,41 | 23,06 |
| MIAT | 2,91 | 1,21E-06 | 6,20 | 0,79 |
| AC004921.1 | 2,91 | 6,88E-03 | 3,72 | 0,46 |
| HIVEP3 | 2,91 | 4,57E-05 | 2,61 | 0,61 |
| AIM2 | 2,91 | 2,41E-03 | 14,52 | 1,53 |
| SLC43A3 | 2,91 | 1,82E-04 | 118,38 | 14,32 |
| GPR171 | 2,91 | 2,28E-03 | 15,55 | 1,76 |
| BATF3 | 2,92 | 5,24E-03 | 9,12 | 1,31 |
| FERMT3 | 2,93 | 2,05E-03 | 144,43 | 17,21 |
| PIK3CG | 2,93 | 1,29E-04 | 14,14 | 1,63 |
| CEACAM3 | 2,93 | 6,60E-03 | 136,96 | 15,30 |
| HIVEP2 | 2,93 | 1,94E-04 | 42,44 | 5,32 |
| GPR137B | 2,94 | 1,53E-06 | 50,43 | 6,50 |
| AC079209.1 | 2,94 | 8,77E-03 | 16,38 | 1,68 |
| STEAP4 | 2,95 | 7,94E-04 | 191,47 | 21,27 |
| GAB3 | 2,95 | 2,87E-07 | 12,21 | 1,27 |
| SIRPB2 | 2,95 | 1,71E-05 | 39,06 | 4,62 |
| IFI30 | 2,95 | 5,68E-05 | 1449,47 | 166,01 |
| SLAMF1 | 2,96 | 2,15E-03 | 21,03 | 2,39 |
| SIGLEC9 | 2,96 | 2,24E-03 | 41,58 | 4,54 |
| CD68 | 2,96 | 5,49E-07 | 387,84 | 44,21 |
| DDX60L | 2,96 | 5,50E-03 | 157,63 | 15,33 |
| RASGRP4 | 2,97 | 3,61E-03 | 56,21 | 5,67 |
| PTPRJ | 2,97 | 1,36E-04 | 64,78 | 7,80 |
| CD33 | 2,97 | 2,05E-07 | 15,60 | 2,59 |
| APBB1IP | 2,97 | 3,09E-03 | 75,84 | 8,21 |
| SASH3 | 2,98 | 5,39E-06 | 73,62 | 7,77 |
| NARF-IT1 | 2,99 | 8,71E-04 | 2,11 | 0,23 |
| P2RX5 | 2,99 | 8,67E-04 | 2,43 | 0,36 |
| PLIN4 | 2,99 | 2,34E-05 | 8,24 | 0,97 |
| DGAT2 | 2,99 | 2,03E-03 | 127,27 | 13,77 |
| HORMAD1 | 2,99 | 9,31E-03 | 2,16 | 0,26 |
| AC006033.2 | 3,00 | 1,12E-03 | 2,38 | 0,30 |
| JAK3 | 3,00 | 3,91E-04 | 53,06 | 5,74 |
| CPNE5 | 3,00 | 5,11E-04 | 1,96 | 0,26 |
| C3AR1 | 3,00 | 7,85E-03 | 246,32 | 25,46 |
| GPNMB | 3,01 | 8,08E-07 | 366,97 | 43,98 |
| ZMIZ1-AS1 | 3,01 | 2,78E-03 | 7,47 | 0,94 |
| SERPINB9P1 | 3,01 | 3,35E-03 | 13,82 | 1,81 |
| SMIM25 | 3,01 | 2,12E-03 | 223,39 | 26,07 |
| CYP1B1 | 3,02 | 1,16E-04 | 4,53 | 0,53 |
| LILRB3 | 3,02 | 4,21E-03 | 185,19 | 17,13 |
| LINC01215 | 3,02 | 2,64E-03 | 3,26 | 0,34 |
| SIGLEC1 | 3,03 | 1,76E-06 | 2,56 | 0,30 |
| DYSF | 3,03 | 4,42E-03 | 51,48 | 5,14 |
| ETV7 | 3,03 | 3,12E-04 | 13,12 | 1,61 |
| ITGB2 | 3,03 | 1,71E-05 | 295,91 | 32,00 |
| SLC2A5 | 3,03 | 6,70E-06 | 10,93 | 1,07 |
| TREML2 | 3,04 | 9,74E-03 | 36,21 | 3,67 |
| SCARF1 | 3,04 | 2,98E-03 | 55,40 | 5,55 |
| NCF1 | 3,04 | 6,27E-03 | 292,07 | 29,37 |
| CSF3R | 3,04 | 4,83E-03 | 1623,40 | 151,90 |
| GAS7 | 3,05 | 5,23E-06 | 23,23 | 2,63 |
| DOK3 | 3,05 | 7,51E-04 | 151,86 | 15,09 |
| F5 | 3,05 | 1,19E-03 | 17,61 | 2,40 |
| CLEC12A | 3,06 | 7,34E-03 | 75,67 | 8,33 |
| MEI1 | 3,06 | 6,35E-05 | 26,89 | 3,52 |
| CCL2 | 3,06 | 1,66E-03 | 364,37 | 38,64 |
| NAP1L4P1 | 3,07 | 1,58E-04 | 2,25 | 0,26 |
| BATF2 | 3,07 | 4,05E-05 | 15,09 | 1,81 |
| AC136475.5 | 3,07 | 6,90E-03 | 13,68 | 1,41 |
| CYTH4 | 3,07 | 2,42E-03 | 236,33 | 22,56 |
| AC098613.1 | 3,08 | 7,93E-03 | 13,48 | 1,28 |
| NECAB2 | 3,09 | 1,22E-03 | 6,11 | 0,71 |
| SP140 | 3,09 | 6,51E-05 | 19,34 | 3,09 |
| IKZF3 | 3,09 | 2,49E-05 | 3,54 | 0,41 |
| CHI3L2 | 3,10 | 5,21E-03 | 22,48 | 3,09 |
| LINC01857 | 3,10 | 8,37E-03 | 12,13 | 1,21 |
| RAC2 | 3,11 | 1,43E-03 | 299,12 | 30,01 |
| FGR | 3,11 | 3,79E-03 | 372,75 | 35,83 |
| IL3RA | 3,12 | 6,62E-07 | 16,97 | 1,99 |
| ZBP1 | 3,12 | 2,69E-05 | 17,59 | 2,19 |
| LINC01001 | 3,12 | 5,65E-05 | 128,96 | 14,67 |
| FAM157A | 3,13 | 7,01E-03 | 39,93 | 3,78 |
| GVINP1 | 3,13 | 1,68E-04 | 7,42 | 0,85 |
| CD14 | 3,13 | 9,33E-04 | 819,90 | 80,71 |
| LINC02328 | 3,13 | 3,44E-03 | 2,23 | 0,23 |
| TIFA | 3,14 | 4,00E-04 | 105,59 | 10,36 |
| LILRB2 | 3,15 | 1,02E-03 | 104,35 | 11,23 |
| PIK3R5 | 3,15 | 1,81E-03 | 101,89 | 9,51 |
| FCGR1B | 3,16 | 5,01E-03 | 183,73 | 14,22 |
| PRDM8 | 3,16 | 1,79E-03 | 18,24 | 1,81 |
| ALPL | 3,16 | 5,50E-03 | 175,11 | 16,37 |
| HELB | 3,16 | 3,46E-04 | 6,15 | 0,74 |
| LILRA6 | 3,17 | 2,14E-03 | 178,60 | 17,06 |
| PSTPIP2 | 3,17 | 1,02E-03 | 96,70 | 9,87 |
| SEMA7A | 3,18 | 2,87E-07 | 12,68 | 1,49 |
| PLEKHM3 | 3,19 | 1,38E-04 | 10,24 | 1,11 |
| APOBR | 3,19 | 7,87E-05 | 81,42 | 7,55 |
| FCGR1A | 3,19 | 8,78E-04 | 323,24 | 34,20 |
| FYB1 | 3,20 | 6,98E-04 | 196,75 | 18,26 |
| CD163 | 3,20 | 4,65E-06 | 128,67 | 13,24 |
| AC007728.2 | 3,20 | 1,59E-03 | 3,65 | 0,36 |
| AC112496.1 | 3,20 | 4,34E-03 | 30,27 | 2,76 |
| AL390066.1 | 3,21 | 6,93E-06 | 6,27 | 0,68 |
| PNRC1 | 3,21 | 2,28E-03 | 768,50 | 79,02 |
| HTRA4 | 3,21 | 2,03E-04 | 8,18 | 0,80 |
| KCNA3 | 3,21 | 4,60E-04 | 2,49 | 0,27 |
| MARCO | 3,23 | 4,64E-04 | 78,93 | 8,58 |
| OAS3 | 3,23 | 1,47E-07 | 23,39 | 2,41 |
| LILRB4 | 3,23 | 2,94E-05 | 99,40 | 10,65 |
| MYBPC3 | 3,23 | 5,25E-04 | 2,17 | 0,18 |
| HCK | 3,23 | 2,38E-03 | 417,66 | 37,90 |
| SIGLEC10 | 3,24 | 1,33E-03 | 72,57 | 8,16 |
| IFIT3 | 3,24 | 1,07E-04 | 137,98 | 12,44 |
| ZEB2 | 3,24 | 5,62E-04 | 126,68 | 10,93 |
| FAM129A | 3,25 | 9,00E-04 | 193,61 | 18,51 |
| SIRPA | 3,25 | 7,87E-05 | 178,49 | 17,52 |
| MYO1G | 3,26 | 5,60E-05 | 32,16 | 3,40 |
| TMEM121B | 3,26 | 5,43E-06 | 4,06 | 0,39 |
| NLRP6 | 3,26 | 9,20E-03 | 6,85 | 0,60 |
| AL732372.3 | 3,27 | 1,70E-05 | 35,15 | 3,63 |
| IL18BP | 3,27 | 3,68E-10 | 40,51 | 3,81 |
| SNX20 | 3,27 | 1,90E-06 | 39,11 | 3,53 |
| GAS2L3 | 3,28 | 3,84E-07 | 6,43 | 0,69 |
| RSAD2 | 3,29 | 1,02E-04 | 21,91 | 2,26 |
| RPL7AP64 | 3,29 | 3,38E-04 | 2,32 | 0,24 |
| AC099489.1 | 3,30 | 1,40E-03 | 51,45 | 4,16 |
| ADGRE2 | 3,30 | 1,21E-03 | 116,04 | 10,56 |
| ITGAX | 3,30 | 1,03E-04 | 338,14 | 26,34 |
| ST18 | 3,32 | 3,00E-06 | 2,34 | 0,30 |
| LINC01010 | 3,33 | 2,64E-03 | 2,95 | 0,29 |
| JAKMIP2 | 3,33 | 2,07E-04 | 7,29 | 0,69 |
| AC092484.1 | 3,34 | 3,55E-03 | 2,28 | 0,20 |
| AC002091.1 | 3,36 | 3,70E-03 | 7,50 | 0,64 |
| CSF2RB | 3,37 | 6,38E-03 | 323,10 | 26,44 |
| PNPLA1 | 3,38 | 5,35E-03 | 5,49 | 0,42 |
| ITGAM | 3,38 | 1,34E-06 | 66,34 | 5,75 |
| FAM157B | 3,39 | 2,48E-04 | 40,18 | 3,46 |
| MICB | 3,40 | 2,81E-07 | 10,91 | 1,05 |
| ATP6V0D2 | 3,41 | 8,14E-07 | 4,01 | 0,37 |
| LILRB5 | 3,42 | 3,31E-09 | 2,72 | 0,26 |
| TMEM140 | 3,42 | 4,80E-05 | 124,45 | 9,90 |
| NOD2 | 3,42 | 2,21E-08 | 31,35 | 2,74 |
| IL2RA | 3,43 | 2,62E-04 | 11,50 | 1,16 |
| AC019254.1 | 3,43 | 8,54E-03 | 3,05 | 0,39 |
| CYP27B1 | 3,46 | 1,68E-04 | 6,09 | 0,66 |
| TNFRSF9 | 3,46 | 7,00E-04 | 13,49 | 1,03 |
| NEURL3 | 3,47 | 1,05E-03 | 2,40 | 0,26 |
| LINC01270 | 3,48 | 4,79E-05 | 18,33 | 1,31 |
| AC139495.3 | 3,48 | 1,67E-06 | 11,77 | 0,90 |
| FFAR3 | 3,49 | 8,39E-03 | 7,02 | 0,61 |
| TM4SF19 | 3,49 | 8,77E-05 | 10,18 | 0,89 |
| CD84 | 3,50 | 3,68E-10 | 34,11 | 2,48 |
| LINC01146 | 3,50 | 8,27E-03 | 2,78 | 0,22 |
| CD22 | 3,51 | 6,06E-07 | 15,86 | 1,88 |
| AC106865.1 | 3,51 | 2,85E-03 | 3,85 | 0,23 |
| PTGER4 | 3,51 | 1,27E-04 | 29,21 | 2,74 |
| CLEC6A | 3,51 | 1,70E-03 | 14,43 | 1,16 |
| MRVI1 | 3,52 | 3,82E-05 | 20,26 | 1,50 |
| NCF1B | 3,53 | 4,07E-04 | 137,60 | 9,78 |
| SEMA6B | 3,54 | 1,94E-03 | 31,57 | 2,37 |
| MMP2-AS1 | 3,54 | 6,23E-03 | 6,20 | 0,54 |
| PDCD1LG2 | 3,54 | 1,90E-06 | 9,94 | 0,76 |
| MMP25 | 3,55 | 2,54E-03 | 245,92 | 13,82 |
| AL627309.5 | 3,57 | 2,04E-04 | 156,17 | 13,22 |
| EGR2 | 3,58 | 4,52E-05 | 15,57 | 1,15 |
| CD40 | 3,58 | 3,04E-05 | 54,99 | 4,95 |
| AL109809.1 | 3,59 | 1,02E-03 | 3,83 | 0,30 |
| HSD11B1 | 3,59 | 1,85E-04 | 11,65 | 0,86 |
| CMKLR1 | 3,60 | 6,59E-09 | 10,91 | 0,89 |
| CYBB | 3,62 | 5,92E-06 | 368,48 | 29,17 |
| SLC26A8 | 3,62 | 3,45E-05 | 2,59 | 0,25 |
| AC012441.1 | 3,62 | 4,34E-03 | 2,92 | 0,25 |
| LINC01268 | 3,63 | 2,25E-03 | 5,13 | 0,37 |
| GBP1 | 3,65 | 1,13E-03 | 439,82 | 28,25 |
| NRROS | 3,66 | 2,92E-11 | 11,39 | 0,84 |
| CLEC12B | 3,66 | 1,04E-03 | 7,29 | 0,53 |
| AC020909.2 | 3,67 | 3,30E-03 | 2,30 | 0,16 |
| ADAMDEC1 | 3,67 | 3,38E-04 | 68,90 | 5,32 |
| SGTB | 3,67 | 2,77E-05 | 20,49 | 1,86 |
| GBP1P1 | 3,67 | 6,08E-04 | 6,97 | 0,55 |
| ARHGAP31 | 3,68 | 8,84E-07 | 10,39 | 1,15 |
| DLEU7 | 3,68 | 2,95E-07 | 5,25 | 0,39 |
| LINC01181 | 3,69 | 8,93E-03 | 3,05 | 0,23 |
| FAM157C | 3,69 | 3,13E-06 | 112,74 | 8,90 |
| FMNL3 | 3,70 | 5,37E-06 | 26,65 | 2,17 |
| AK5 | 3,70 | 5,43E-06 | 3,23 | 0,48 |
| ZDHHC19 | 3,70 | 7,26E-03 | 3,44 | 0,30 |
| DOCK4 | 3,71 | 8,77E-08 | 44,04 | 3,43 |
| GABRR2 | 3,72 | 4,89E-07 | 3,11 | 0,21 |
| SUCNR1 | 3,72 | 6,90E-07 | 2,03 | 0,13 |
| CCR5 | 3,72 | 2,58E-06 | 48,92 | 3,78 |
| PAG1 | 3,72 | 1,15E-05 | 26,74 | 1,50 |
| LTA | 3,73 | 1,10E-04 | 4,60 | 0,35 |
| TFEC | 3,73 | 1,52E-03 | 83,52 | 6,60 |
| LPL | 3,73 | 2,64E-07 | 9,88 | 0,70 |
| PLA2G7 | 3,75 | 3,51E-06 | 92,90 | 6,72 |
| CR1 | 3,76 | 2,68E-05 | 50,07 | 3,40 |
| DCSTAMP | 3,77 | 2,28E-04 | 5,87 | 0,40 |
| LINC01093 | 3,78 | 6,99E-03 | 28,81 | 1,65 |
| GPR84 | 3,81 | 4,55E-03 | 151,39 | 9,78 |
| NCF1C | 3,85 | 1,13E-05 | 105,71 | 6,97 |
| HLA-L | 3,86 | 6,38E-08 | 6,69 | 0,27 |
| SLAMF7 | 3,87 | 1,76E-03 | 351,72 | 20,99 |
| CD274 | 3,88 | 6,48E-05 | 120,04 | 7,01 |
| EBI3 | 3,93 | 2,96E-04 | 22,61 | 1,65 |
| UPB1 | 3,94 | 5,86E-04 | 7,11 | 0,38 |
| MSC | 3,95 | 4,78E-04 | 6,53 | 0,63 |
| SLC2A6 | 3,96 | 7,02E-05 | 29,17 | 1,91 |
| SLAMF8 | 3,96 | 3,96E-09 | 49,12 | 3,33 |
| AC008074.2 | 3,98 | 5,35E-03 | 2,65 | 0,12 |
| METTL7B | 4,01 | 2,78E-08 | 9,66 | 0,53 |
| AP000295.1 | 4,03 | 5,35E-03 | 4,22 | 0,27 |
| HSPE1P18 | 4,06 | 7,46E-03 | 4,18 | 0,25 |
| MMP9 | 4,07 | 5,47E-08 | 337,77 | 20,29 |
| RAB39A | 4,08 | 3,63E-06 | 3,26 | 0,19 |
| AC002511.1 | 4,08 | 7,55E-03 | 1,98 | 0,09 |
| LINC01762 | 4,11 | 3,45E-03 | 3,05 | 0,20 |
| KCNJ2 | 4,12 | 1,98E-03 | 96,88 | 4,49 |
| GBP5 | 4,17 | 1,20E-05 | 338,16 | 17,46 |
| PTGIR | 4,19 | 9,66E-06 | 10,34 | 0,57 |
| CAMK1G | 4,19 | 3,76E-04 | 9,05 | 0,35 |
| AL359532.1 | 4,25 | 3,94E-07 | 2,98 | 0,14 |
| LINC02555 | 4,26 | 1,13E-03 | 5,69 | 0,24 |
| C1orf61 | 4,26 | 1,52E-03 | 3,61 | 0,44 |
| CXCL9 | 4,28 | 1,18E-06 | 36,53 | 1,84 |
| SMPD5 | 4,28 | 5,06E-05 | 2,93 | 0,14 |
| TMEM150B | 4,30 | 1,83E-05 | 4,06 | 0,19 |
| AP001056.1 | 4,32 | 1,24E-03 | 2,39 | 0,12 |
| AC002511.2 | 4,36 | 7,75E-05 | 7,42 | 0,35 |
| GBP4 | 4,36 | 6,60E-07 | 69,60 | 4,14 |
| MIR155HG | 4,38 | 6,36E-04 | 21,83 | 0,92 |
| P2RY14 | 4,41 | 9,34E-06 | 36,17 | 1,36 |
| P2RX7 | 4,42 | 2,81E-07 | 54,03 | 2,90 |
| KCNJ2-AS1 | 4,45 | 1,33E-04 | 6,50 | 0,25 |
| COL13A1 | 4,46 | 1,11E-05 | 4,00 | 0,22 |
| TRBV3-1 | 4,54 | 5,55E-03 | 3,98 | 0,17 |
| AC007728.3 | 4,56 | 1,49E-06 | 4,78 | 0,18 |
| AC007278.1 | 4,62 | 2,47E-04 | 8,73 | 0,30 |
| AC004988.1 | 4,84 | 8,03E-04 | 1,99 | 0,08 |
| KCNH4 | 4,84 | 3,26E-08 | 2,25 | 0,08 |
| SPATC1 | 4,85 | 1,11E-04 | 6,03 | 0,20 |
| AC093583.1 | 5,07 | 2,45E-04 | 7,03 | 0,22 |
| AL591846.1 | 5,23 | 1,08E-04 | 14,92 | 0,37 |
| AL121985.1 | 5,25 | 9,88E-08 | 14,28 | 0,35 |
| AC091808.1 | 5,69 | 1,49E-03 | 2,93 | 0,06 |
| AL365357.1 | 7,26 | 3,21E-05 | 14,64 | 0,05 |
