# Supplementary material for: The cervical transcriptome changes during the menstrual cycle but does not predict the window of implantation
Source: Front Reprod Health. 2023 Jul 14;5:1224919. doi: 10.3389/frph.2023.1224919 (PMC10375708; doi:10.3389/frph.2023.1224919)
Supplement: Supplementary file 10 [file Table7.docx]

| **Supplementary table 7.** Cellular distribution of cervical cell samples. Average enrichments scores calculated by xCell. | | | | | |
| --- | --- | --- | --- | --- | --- |
|  | **P** | **LH+2** | **LH+7** | **LH+11** | **HRC: P+5** |
| Smooth muscle | 0.501 | 0.538 | 0.552 | 0.516 | 0.263 |
| Epithelial cells | 0.446 | 0.421 | 0.452 | 0.345 | 0.362 |
| iDC | 0.527 | 0.152 | 0.137 | 0.179 | 0.537 |
| Monocytes | 0.333 | 0.389 | 0.276 | 0.461 | 0.605 |
| Basophils | 0.271 | 0.259 | 0.296 | 0.247 | 0.299 |
| cDC | 0.240 | 0.123 | 0.128 | 0.102 | 0.472 |
| Neutrophils | 0.227 | 0.290 | 0.179 | 0.320 | 0.357 |
| aDC | 0.188 | 0.188 | 0.214 | 0.216 | 0.437 |
| NKT | 0.140 | 0.145 | 0.095 | 0.199 | 0.366 |
| CLP | 0.089 | 0.122 | 0.127 | 0.105 | 0.055 |
| Keratinocytes | 0.080 | 0.094 | 0.099 | 0.030 | 0.065 |
| Osteoblast | 0.073 | 0.037 | 0.068 | 0.032 | 0.037 |
| DC | 0.070 | 0.040 | 0.036 | 0.028 | 0.125 |
| Mesangial cells | 0.068 | 0.037 | 0.035 | 0.056 | 0.015 |
| Macrophages | 0.056 | 0.000 | 0.041 | 0.008 | 0.121 |
| Macrophages M1 | 0.054 | 0.007 | 0.048 | 0.027 | 0.114 |
| Sebocytes | 0.052 | 0.054 | 0.062 | 0.021 | 0.045 |
| CD8+ naive T-cells | 0.041 | 0.056 | 0.076 | 0.053 | 0.059 |
| Th2 cells | 0.033 | 0.087 | 0.026 | 0.035 | 0.047 |
| CD4+ Tem | 0.029 | 0.043 | 0.041 | 0.058 | 0.113 |
| Plasma cells | 0.028 | 0.033 | 0.032 | 0.025 | 0.031 |
| HSC | 0.025 | 0.073 | 0.054 | 0.096 | 0.007 |
| Pericytes | 0.023 | 0.004 | 0.025 | 0.144 | 0.017 |
| Mast cells | 0.021 | 0.020 | 0.023 | 0.025 | 0.038 |
| MEP | 0.021 | 0.020 | 0.018 | 0.013 | 0.003 |
| B-cells | 0.018 | 0.040 | 0.025 | 0.040 | 0.108 |
| pDC | 0.016 | 0.016 | 0.011 | 0.031 | 0.073 |
| Class-switched memory B-cells | 0.011 | 0.019 | 0.015 | 0.021 | 0.060 |
| Tregs | 0.009 | 0.002 | 0.004 | 0.008 | 0.079 |
| Fibroblasts | 0.005 | 0.000 | 0.012 | 0.085 | 0.000 |
| Endothelial cells | 0.004 | 0.000 | 0.005 | 0.029 | 0.000 |
| CD8+ Tcm | 0.003 | 0.031 | 0.020 | 0.019 | 0.084 |
| naive B-cells | 0.001 | 0.003 | 0.002 | 0.002 | 0.011 |
| Memory B-cells | 0.001 | 0.001 | 0.001 | 0.001 | 0.014 |
| CD8+ T-cells | 0.000 | 0.015 | 0.018 | 0.005 | 0.039 |

iDC- interstitial dendritic cells, cDC-classic dendritic cells, aDC- activated dendritic cells, NKT- natural killer T cells, CLP- common lymphoid progenitor cells, DC-dendritic cells, HSC-hematopoietic stem cells, MEP- megakaryocyte-erythroid progenitor cells, pDC- plasmacytoid dendritic cells
